# Supplementary material for: PIWI proteins tether the piRNA biogenesis machinery to mitochondria during mammalian spermatogenesis
Source: EMBO J. 2025 Sep 29;44(22):6397–424. doi: 10.1038/s44318-025-00579-x (PMC12624062; doi:10.1038/s44318-025-00579-x)
Supplement: Supplementary file 1 — Appendix [file 44318_2025_579_MOESM1_ESM.pdf]

Appendix for:

PIWI proteins tether piRNA biogenesis machinery to mitochondria in mice

Jie Gao, Canmei Chen, Guanyi Shang, Wenyang Yu, Ting Zhao, Yunfang Zhang, Chen Chen and Deqiang Ding

* Correspondence: dingdeqiang@tongji.edu.cn

Table of Contents:

Appendix Figure S1. GPAT2 and mitoPLD could not recruit piRNA pathway proteins to mitochondria..2

Appendix Figure S2. TDRD1 is required for IMC assembly and pachytene piRNA biogenesis in adult testes...3-4

Appendix Table S1. Constructs used in this study...5-7

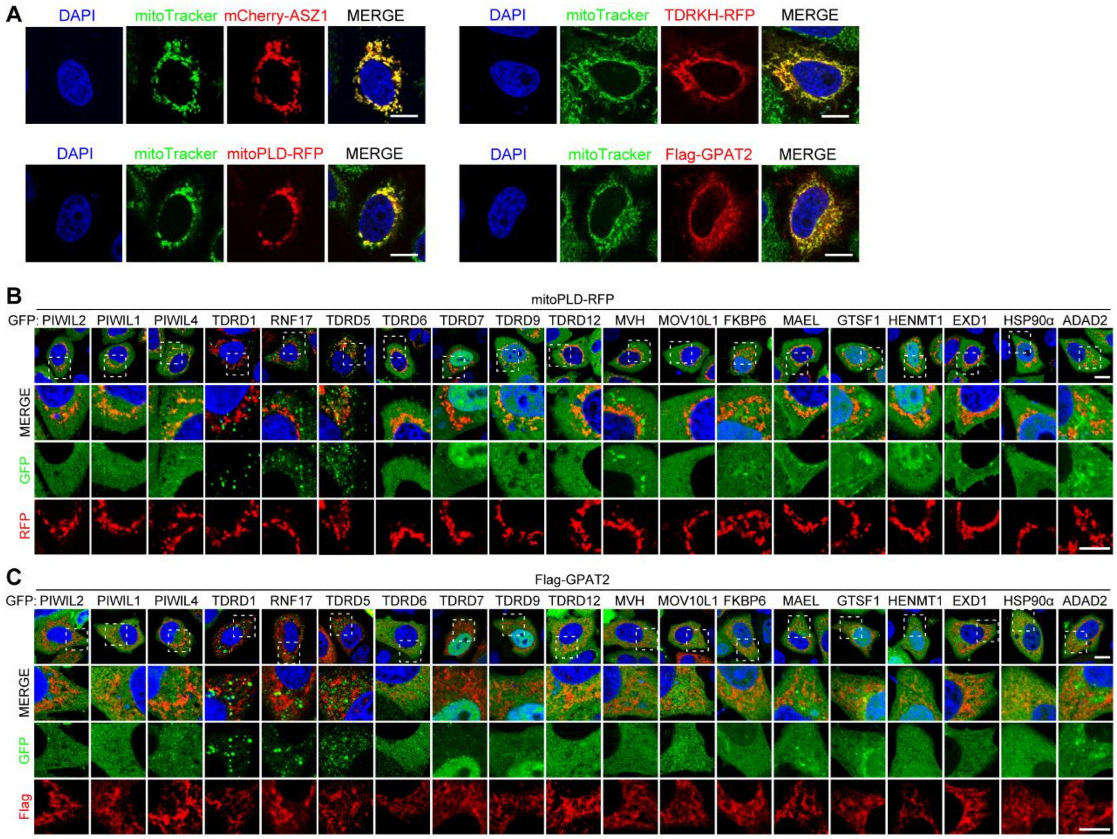

Appendix Figure S1. GPAT2 and mitoPLD could not recruit piRNA pathway proteins to mitochondria.

A HeLa cells were transfected with indicated constructs. Transfected cells were stained with Mitotracker to label mitochondria. Immunostaining was performed using Flag antibody. Scale bars, 10 µm.

B,C Images of HeLa cells transfected with indicated plasmids. Dotted boxes indicate the zoomed-in areas showing in below. Scale bars, 10 µm.

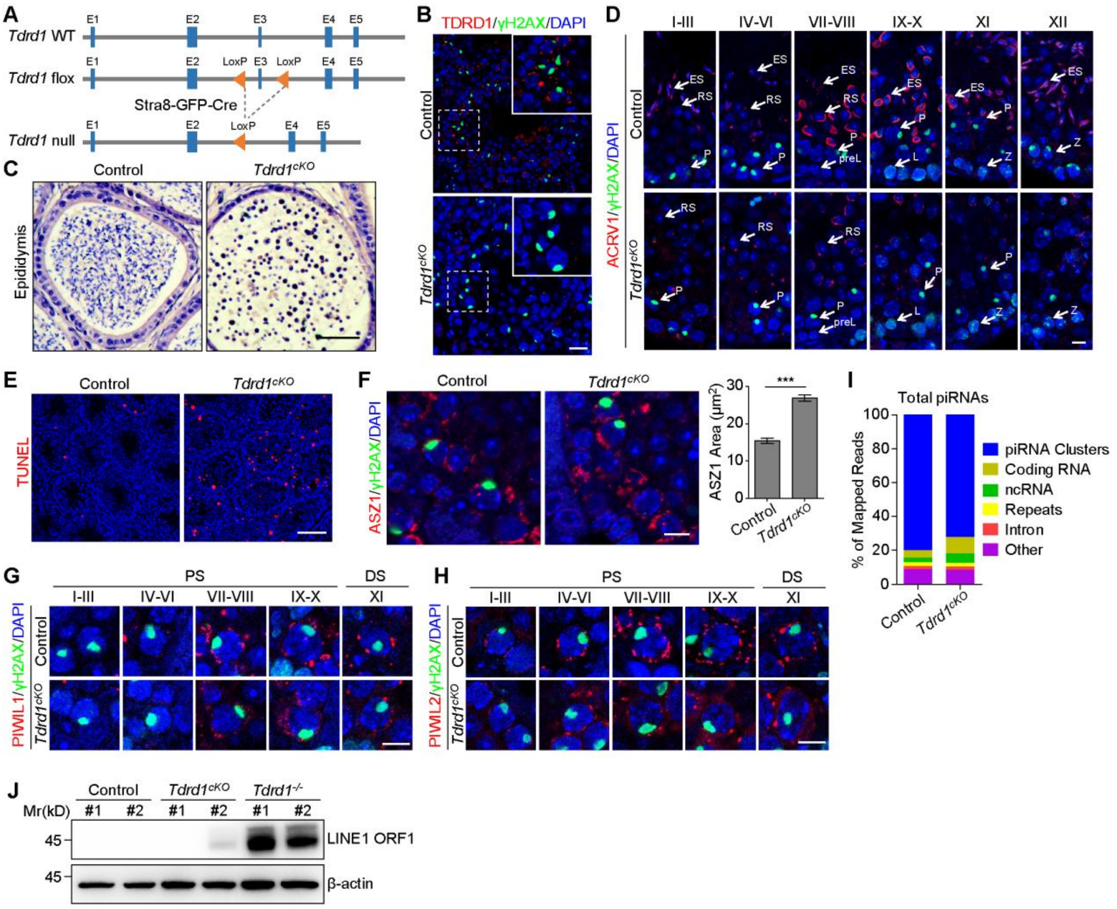

Appendix Figure S2. TDRD1 is required for IMC assembly and pachytene piRNA biogenesis in adult testes.

A A schematic diagram showing the gene targeting strategy for the generation of a *Tdrd1* conditional allele. Cre-mediated deletion removed the exon 3 of *Tdrd1* and generated a protein null allele.

B Co-immunostaining of TDRD1 and γH2AX on control and *Tdrd1^{cKO}* adult testes. Scale bars, 20 µm.

C H&E staining on epididymis from adult mice. Scale bars, 50 µm.

D Co-immunostaining of ACRV1 and γH2AX on adult mouse testes. preL, prelepotene; L, lepotene; Z, zygotene; P, pachytene; D, diplotene; M, metaphase; RS, round spermatid; ES, elongated spermatid; Scale bars, 10 µm.

E TUNEL assays on adult testes. Scale bars, 100 µm.

F Co-immunostaining of ASZ1 and γH2AX on adult testes. Scale bars, 10 µm. Mitochondrial areas corresponding to ASZ1 immunostaining signal in each pachytene spermatocytes (VII-VIII) are shown on the right. ($n = 50$; ***$p < 0.001$).

G,H Co-immunostaining of PIWIL1 and γH2AX (G) or PIWIL2 and γH2AX (H) on adult testes. PS, pachytene spermatocytes; DS, diplotene spermatocytes; Scale

bars, 10 μm. The developmental stages of germ cells were distinguished according to γH2AX and DAPI staining.

I Genomic annotation of piRNAs from control and *Tdrd1*^{cKO} adult testes. Data are representative of 3 biological replicates.

J WB of LINE1 ORF1 in adult testes. β-actin served as a control.

Data information: In (F), data are presented as mean ± s.e.m and *p* values were calculated using Student's t-test (***$p < 0.001$). $p = 8.6541E-13$ (F).

Appendix Table S1. Constructs used in this study.

Construct	Source	Uniprot ID	Insert Sequence Region	Backbone
Flag	(Gao *et al*, 2024)	N/A	N/A	pcDNA3-Flag
Flag-TDRD1	(Gao *et al.*, 2024)	Q99MV1	1-1172	pcDNA3-Flag
Flag-TDRD1-3GS	(Gao *et al.*, 2024)	Q99MV1	1149-1154: ILLFLL→GSGSGS	pcDNA3-Flag
Flag-TDRKH	(Ding *et al*, 2019)	Q80VL1	1-560	pcDNA3-Flag
Flag-mitoPLD	This paper	Q5SWZ9	1-221	pcDNA3-Flag
Flag-GPAT2	This paper	Q14DK4	1-801	pcDNA3-Flag
Flag-PNLDC1	(Ding *et al.*, 2019)	B2RXZ1	1-531	pcDNA3-Flag
Flag-ASZ1	(Ding *et al.*, 2019)	Q8VD46	1-475	pcDNA3-Flag
Flag-PIWIL2	This paper	Q8CDG1	1-971	pcDNA3-Flag
Flag-PIWIL2-RK	This paper	Q8CDG1	9/39/45/74/95/100 /144/156/163 R→K	pcDNA3-Flag
Flag-PIWIL2-Δ1-200	This paper	Q8CDG1	201-971	pcDNA3-Flag
Flag-PIWIL2-Δ201-600	This paper	Q8CDG1	1-200; 601-971	pcDNA3-Flag
Flag-PIWIL2-Δ601-971	This paper	Q8CDG1	1-600	pcDNA3-Flag
Flag-PIWIL2-(1-200)	This paper	Q8CDG1	1-200	pcDNA3-Flag
Flag-PIWIL2-(201-600)	This paper	Q8CDG1	201-600	pcDNA3-Flag
Flag-PIWIL2-(601-971)	This paper	Q8CDG1	601-971	pcDNA3-Flag
Flag-ASZ1	This paper	Q8VD46	1-475	pcDNA3-Flag
Flag-ASZ1-Δ1-32	This paper	Q8VD46	33-475	pcDNA3-Flag
Flag-MOV10L1	This paper	Q99MV5	1-1187	pcDNA3-Flag
Flag-TDRD5	This paper	Q5VCS6	1-1040	pcDNA3-Flag
Flag-PIWIL1	(Wei *et al*, 2023)	Q9JMB7	1-862	pcDNA3-Flag
Flag-PIWIL1-RK	(Wei *et al.*, 2023)	Q9JMB7	4/6/8/10/12/14/49/51/53/80/83/84/85 R→K	pcDNA3-Flag
Flag-PIWIL1-Δ1-278	This paper	Q9JMB7	279-862	pcDNA3-Flag
Flag-PIWIL1-Δ279-555	This paper	Q9JMB7	1-278; 556-862	pcDNA3-Flag
Flag-PIWIL1-Δ556-862	This paper	Q9JMB7	1-555	pcDNA3-Flag
HA	This paper	N/A	N/A	pcDNA3-HA
HA-TDRD1	(Gao *et al.*, 2024)	Q99MV1	1-1172	pcDNA3-HA
HA-PIWIL2	(Gao *et al.*, 2024)	Q8CDG1	1-971	pcDNA3-HA
HA-PIWIL2-RK	This paper	Q8CDG1	9/39/45/74/95/100	pcDNA3-HA

			/144/156/163 R→K	
HA-PIWIL1	This paper	Q9JMB7	1-862	pcDNA3-HA
HA-PIWIL1-(1-278)	This paper	Q9JMB7	1-278	pcDNA3-HA
HA-PIWIL1-(279-555)	This paper	Q9JMB7	279-555	pcDNA3-HA
HA-PIWIL1-(556-862)	This paper	Q9JMB7	556-862	pcDNA3-HA
mCherry-ASZ1	This paper	Q8VD46	1-475	pcDNA3-mCherry
mCherry-TDRD1	This paper	Q99MV1	1-1172	pcDNA3-mCherry
RFP-ASZ1	This paper	Q8VD46	1-475	pcDNA3-RFP
TDRKH-RFP	(Ding *et al.*, 2019)	Q80VL1	1-560	pRFP-N1
mitoPLD-RFP	This paper	Q5SWZ9	1-221	pRFP-N1
TDRKH-GFP	(Gao *et al.*, 2024)	Q80VL1	1-560	pEGFP-N1
mitoPLD-GFP	(Gao *et al.*, 2024)	Q5SWZ9	1-221	pEGFP-N1
GPAT2-GFP	(Gao *et al.*, 2024)	Q14DK4	1-801	pEGFP-N1
GFP-ASZ1	(Gao *et al.*, 2024)	Q8VD46	1-475	pEGFP-C1
GFP-ASZ1-Δ1-32	This paper	Q8VD46	33-475	pEGFP-C1
GFP-PNLDC1	(Gao *et al.*, 2024)	B2RXZ1	1-531	pEGFP-C1
GFP-PIWIL2	(Gao *et al.*, 2024)	Q8CDG1	1-971	pEGFP-C1
GFP-PIWIL2-TM	(Gao *et al.*, 2024)	Q8CDG1 Q8VD46	1-971; 426-475	pEGFP-C1
GFP-PIWIL1	(Ding *et al.*, 2019)	Q9JMB7	1-862	pEGFP-C1
GFP-PIWIL1-TM	This paper	Q9JMB7 Q8VD46	1-862; 426-475	pEGFP-C1
GFP-PIWIL4	(Ding *et al.*, 2019)	Q8CGT6	1-848	pEGFP-C1
GFP-TDRD1	(Gao *et al.*, 2024)	Q99MV1	1-1172	pEGFP-C1
GFP-TDRD1-3GS	(Gao *et al.*, 2024)	Q99MV1	1149-1154: ILLFLL→GSGSGS	pEGFP-C1
GFP-RNF17	(Wei *et al.*, 2023)	Q99MV7	1-1640	pEGFP-C1
GFP-TDRD5	(Ding *et al*, 2020)	Q5VCS6	1-1040	pEGFP-C1
GFP-TDRD6	(Wei *et al.*, 2023)	P61407	1-2134	pEGFP-C1
GFP-TDRD7	This paper	Q8K1H1	1-1086	pEGFP-C1

GFP-TDRD9	This paper	Q14BI7	1-1383	pEGFP-C1
GFP-TDRD12	This paper	Q9CWU0	1-1277	pEGFP-C1
GFP-MVH	This paper	Q61496	1-702	pEGFP-C1
GFP-MOV10L1	(Wei *et al.*, 2023)	Q99MV5	1-1187	pEGFP-C1
GFP-FKBP6	This paper	Q91XW8	1-327	pEGFP-C1
GFP-MAEL	This paper	Q8BVN9	1-434	pEGFP-C1
GFP-GTSF1	This paper	Q9DAN6	1-167	pEGFP-C1
GFP-HENMT1	This paper	Q8CAE2	1-395	pEGFP-C1
GFP-EXD1	This paper	Q8CDF7	1-570	pEGFP-C1
GFP-HSP90α	This paper	P07901	1-733	pEGFP-C1
GFP-ADAD2	This paper	Q9D5P4	1-561	pEGFP-C1

Reference

Ding D, Liu J, Dong K, Melnick AF, Latham KE, Chen C (2019) Mitochondrial membrane-based initial separation of MIWI and MILI functions during pachytene piRNA biogenesis. *Nucleic Acids Res* 47: 2594-2608

Ding D, Wei C, Dong K, Liu J, Stanton A, Xu C, Min J, Hu J, Chen C (2020) LOTUS domain is a novel class of G-rich and G-quadruplex RNA binding domain. *Nucleic Acids Res* 48: 9262-9272

Gao J, Jing J, Shang G, Chen C, Duan M, Yu W, Wang K, Luo J, Song M, Chen K et al (2024) TDRD1 phase separation drives intermitochondrial cement assembly to promote piRNA biogenesis and fertility. *Dev Cell* 59: 2704-2718.e2706

Wei C, Jing J, Yan X, Mann JM, Geng R, Xie H, Demireva EY, Hess RA, Ding D, Chen C (2023) MIWI N-terminal RG motif promotes efficient pachytene piRNA production and spermatogenesis independent of LINE1 transposon silencing. *PLoS Genet* 19: e1011031
