# Supplementary material for: PIWI proteins tether the piRNA biogenesis machinery to mitochondria during mammalian spermatogenesis
Source: EMBO J. 2025 Sep 29;44(22):6397–424. doi: 10.1038/s44318-025-00579-x (PMC12624062; doi:10.1038/s44318-025-00579-x)
Supplement: Supplementary file 6 — Source data Fig. 1 [file 44318_2025_579_MOESM6_ESM.zip › Figure 1/1B/Figure 1B.pdf]

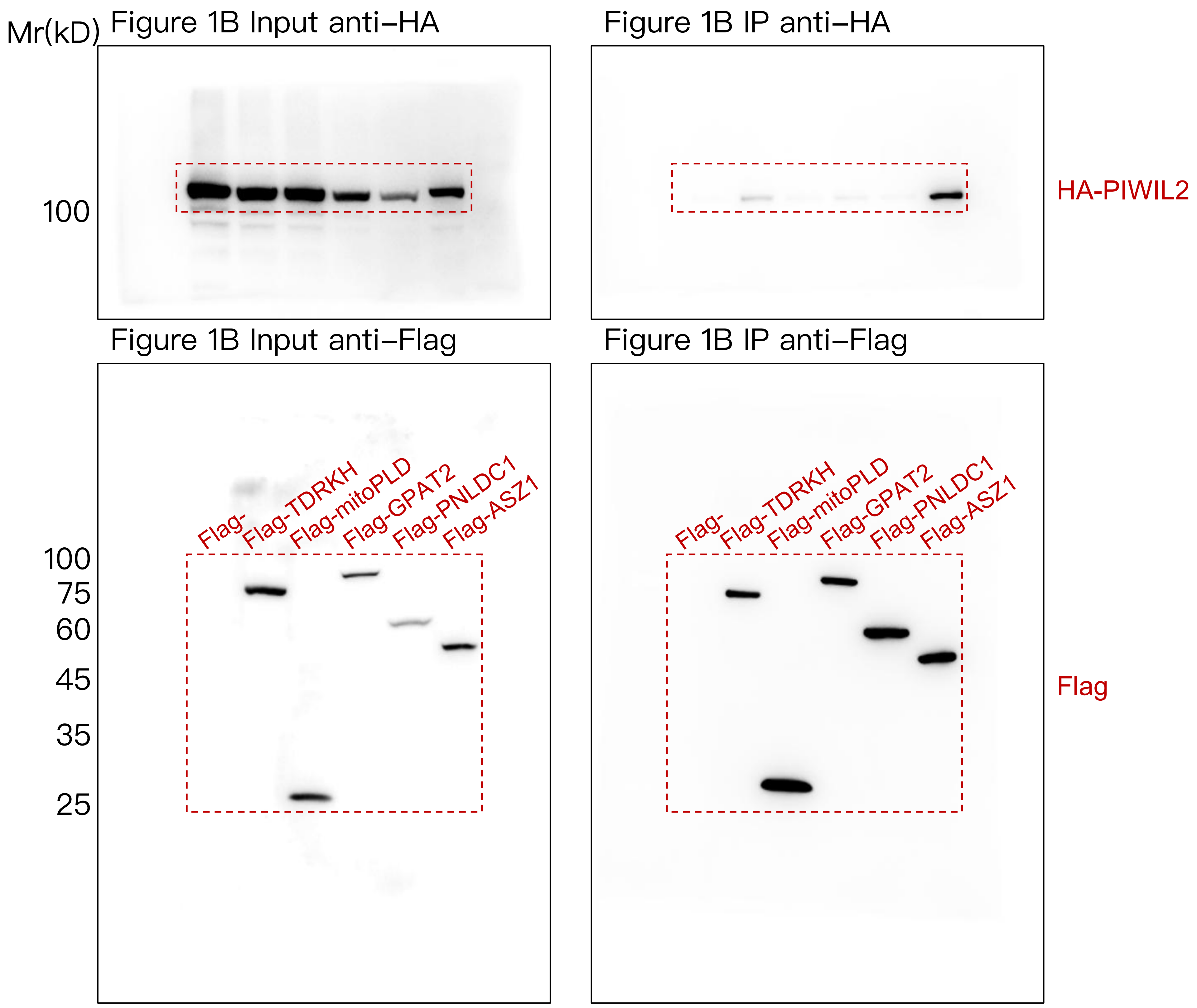
Mr(kD)
Figure 1B Input anti–HA
Figure 1B IP anti–HA
100
HA-PIWIL2
Figure 1B Input anti–Flag
Figure 1B IP anti–Flag
Flag-
Flag-TDRKH
Flag-mitoPLD
Flag-GPAT2
Flag-PNLDC1
Flag-ASZ1
100
75
60
45
35
25
Flag-
Flag-TDRKH
Flag-mitoPLD
Flag-GPAT2
Flag-PNLDC1
Flag-ASZ1
Flag
