# Supplementary material for: PIWI proteins tether the piRNA biogenesis machinery to mitochondria during mammalian spermatogenesis
Source: EMBO J. 2025 Sep 29;44(22):6397–424. doi: 10.1038/s44318-025-00579-x (PMC12624062; doi:10.1038/s44318-025-00579-x)
Supplement: Supplementary file 6 — Source data Fig. 1 [file 44318_2025_579_MOESM6_ESM.zip › Figure 1/1G/Figure 1G.pdf]

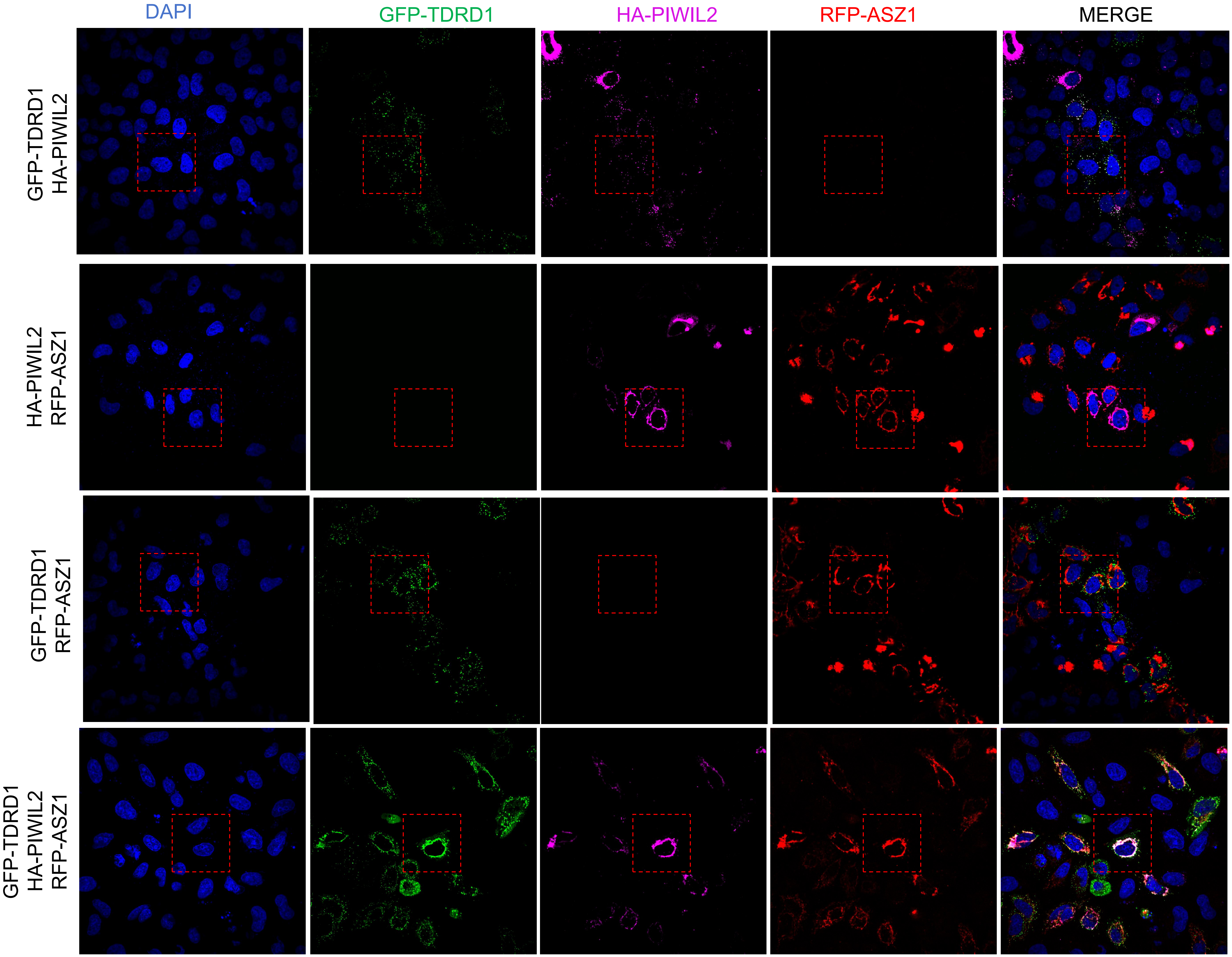
DAPI
GFP-TDRD1
HA-PIWIL2
RFP-ASZ1
MERGE
GFP-TDRD1
HA-PIWIL2
HA-PIWIL2
RFP-ASZ1
GFP-TDRD1
RFP-ASZ1
GFP-TDRD1
HA-PIWIL2
RFP-ASZ1
