# Supplementary figures and images for: PIWI proteins tether the piRNA biogenesis machinery to mitochondria during mammalian spermatogenesis
Source: EMBO J. 2025 Sep 29;44(22):6397–424. doi: 10.1038/s44318-025-00579-x (PMC12624062; doi:10.1038/s44318-025-00579-x)

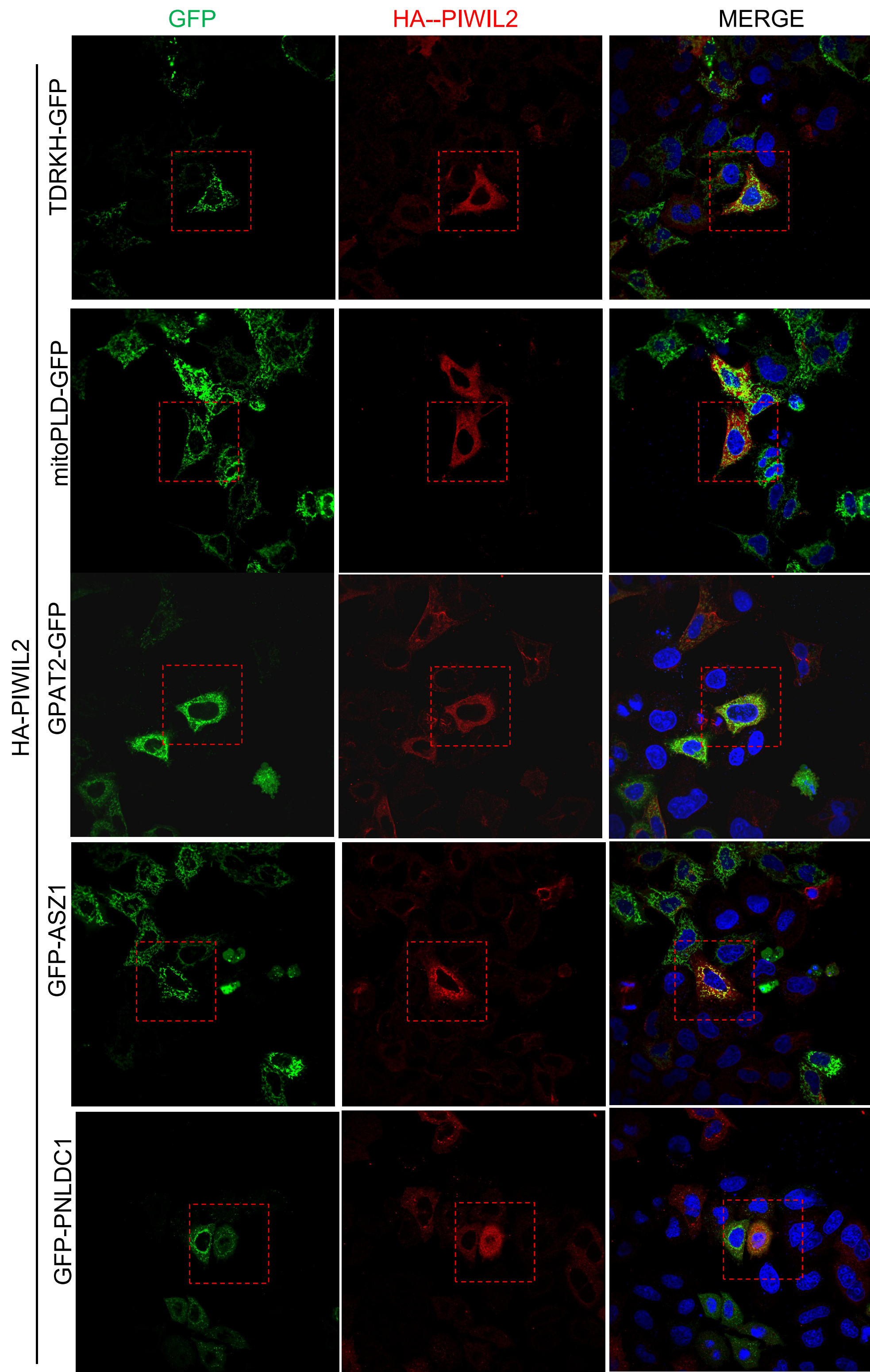

Supplement: Supplementary file 6 — Source data Fig. 1 [file 44318_2025_579_MOESM6_ESM.zip › Figure 1/1A/Figure 1A.pdf]

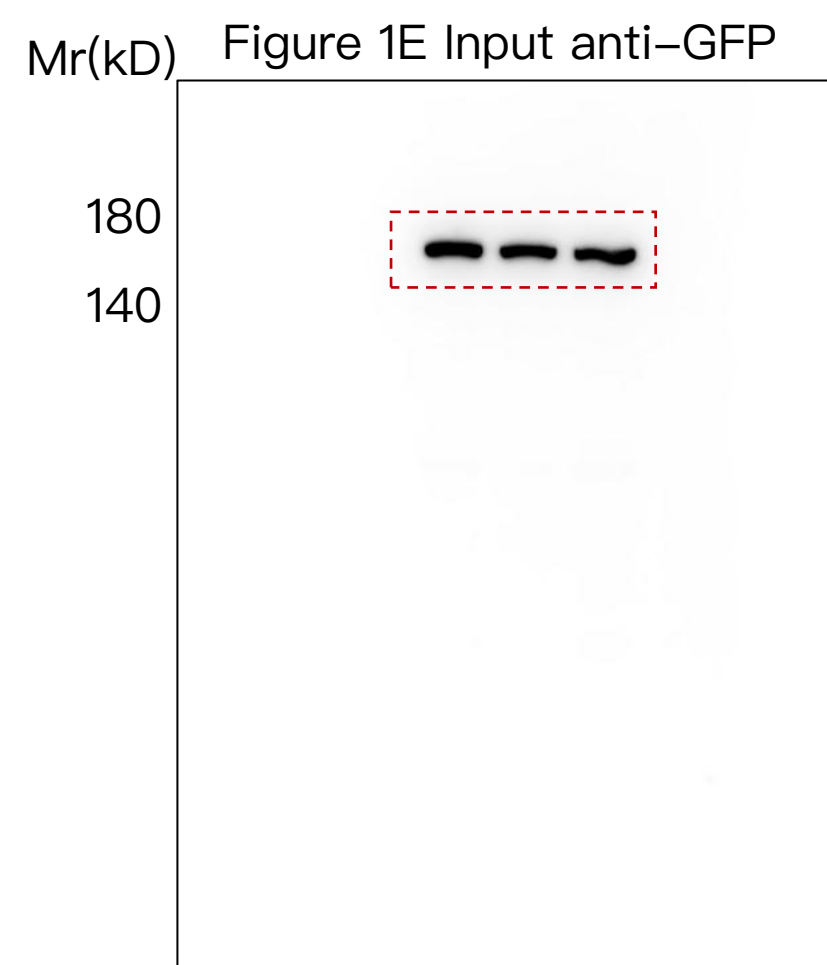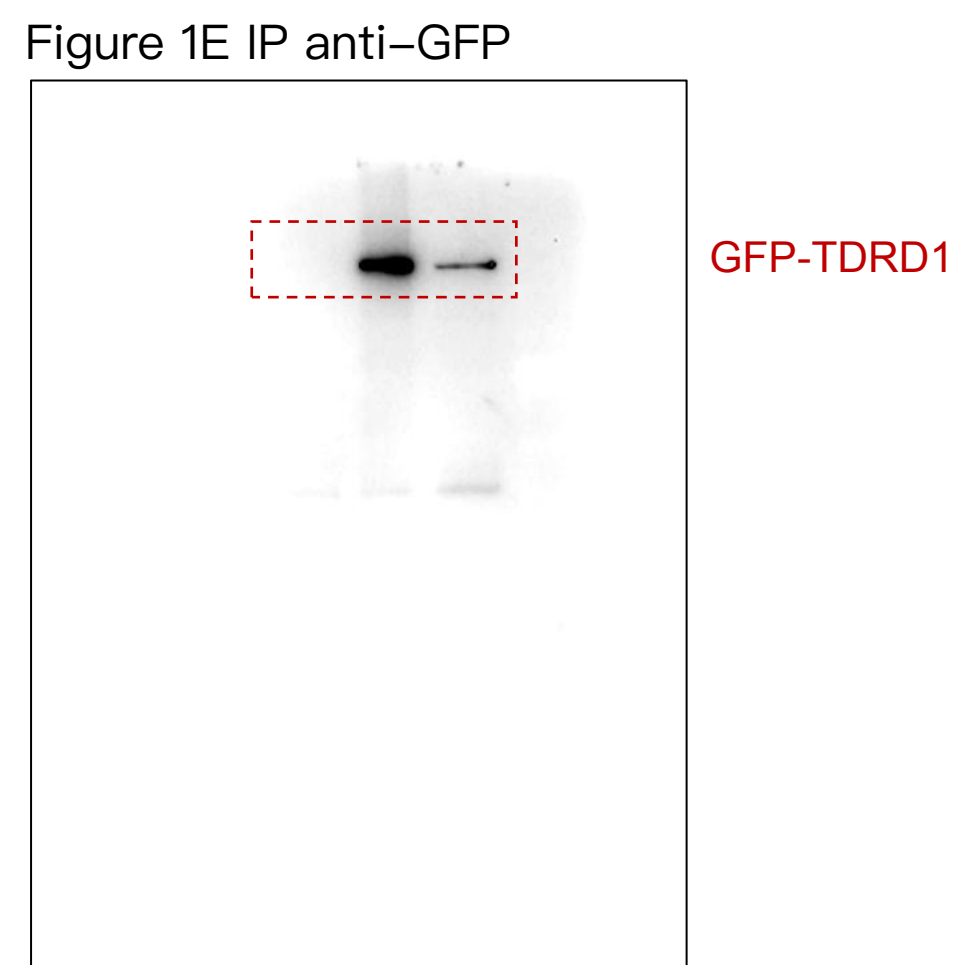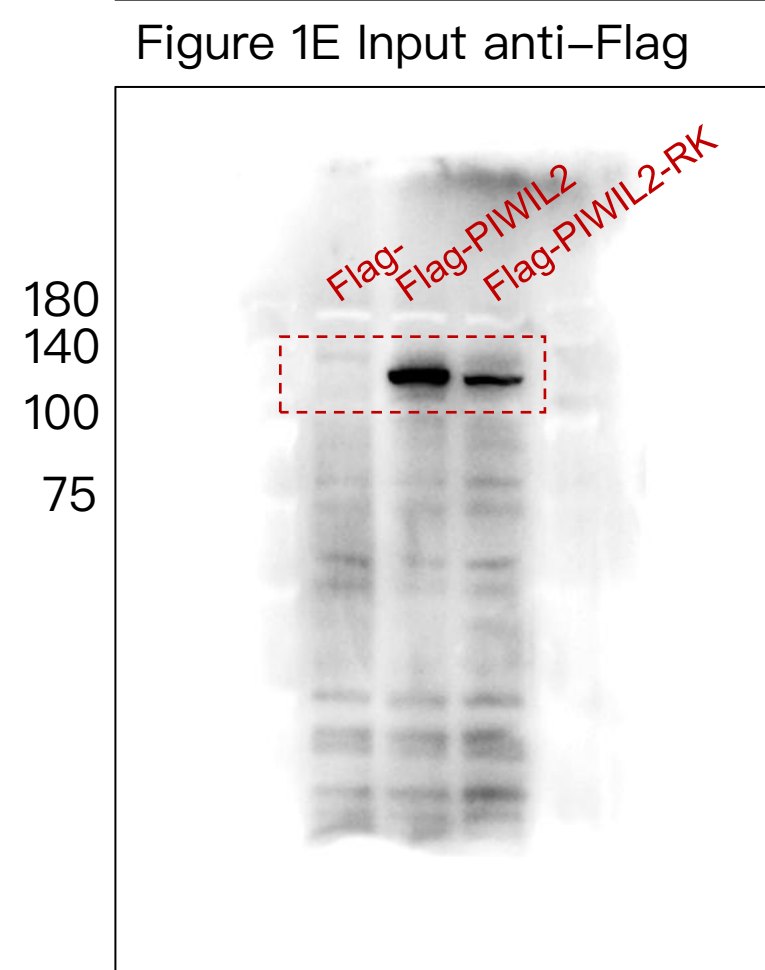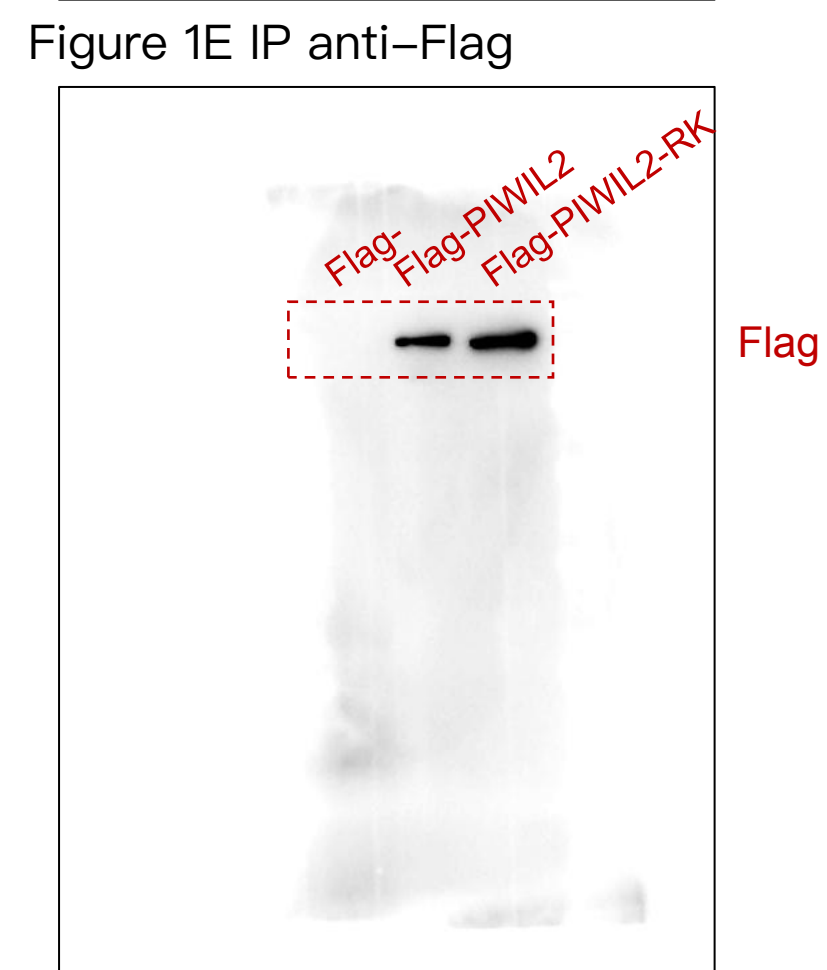

Supplement: Supplementary file 6 — Source data Fig. 1 [file 44318_2025_579_MOESM6_ESM.zip › Figure 1/1E/Figure 1E.pdf]

Figure 1F Input anti-Flag

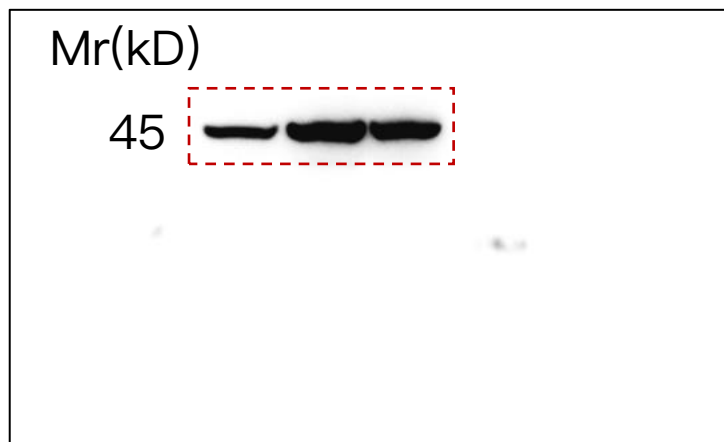

Figure 1F IP anti-Flag

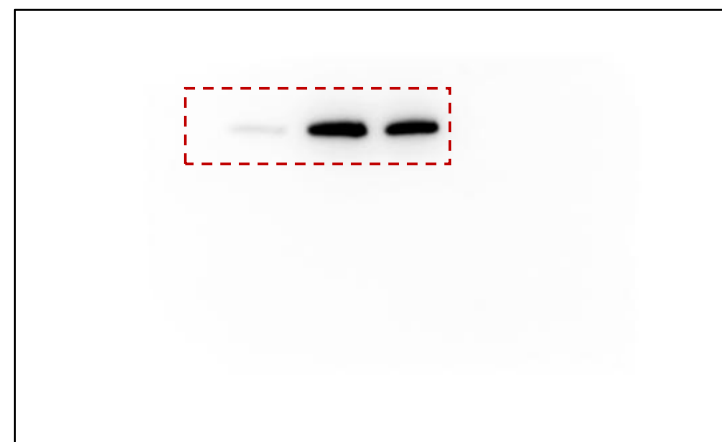

Flag-ASZ1

Figure 1F Input anti-HA

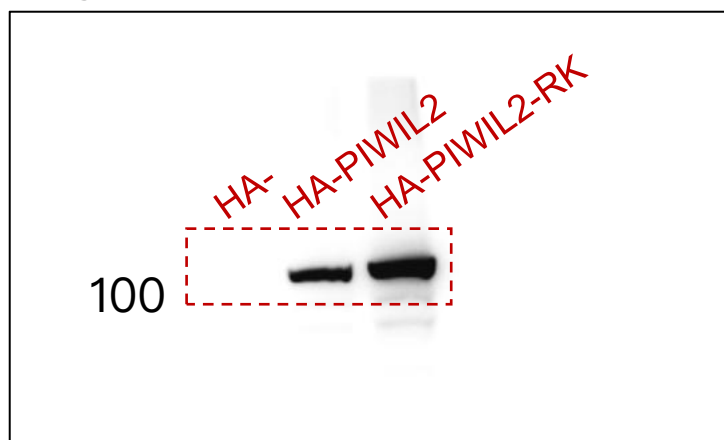

Figure 1F IP anti-HA

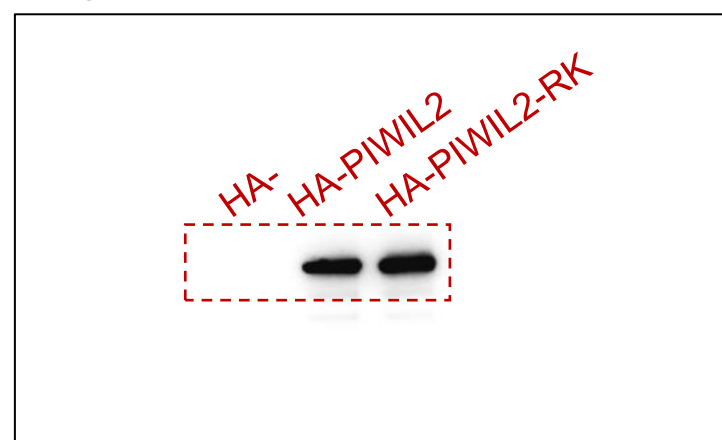

HA

Supplement: Supplementary file 6 — Source data Fig. 1 [file 44318_2025_579_MOESM6_ESM.zip › Figure 1/1F/Figure 1F.pdf]

Negative Control

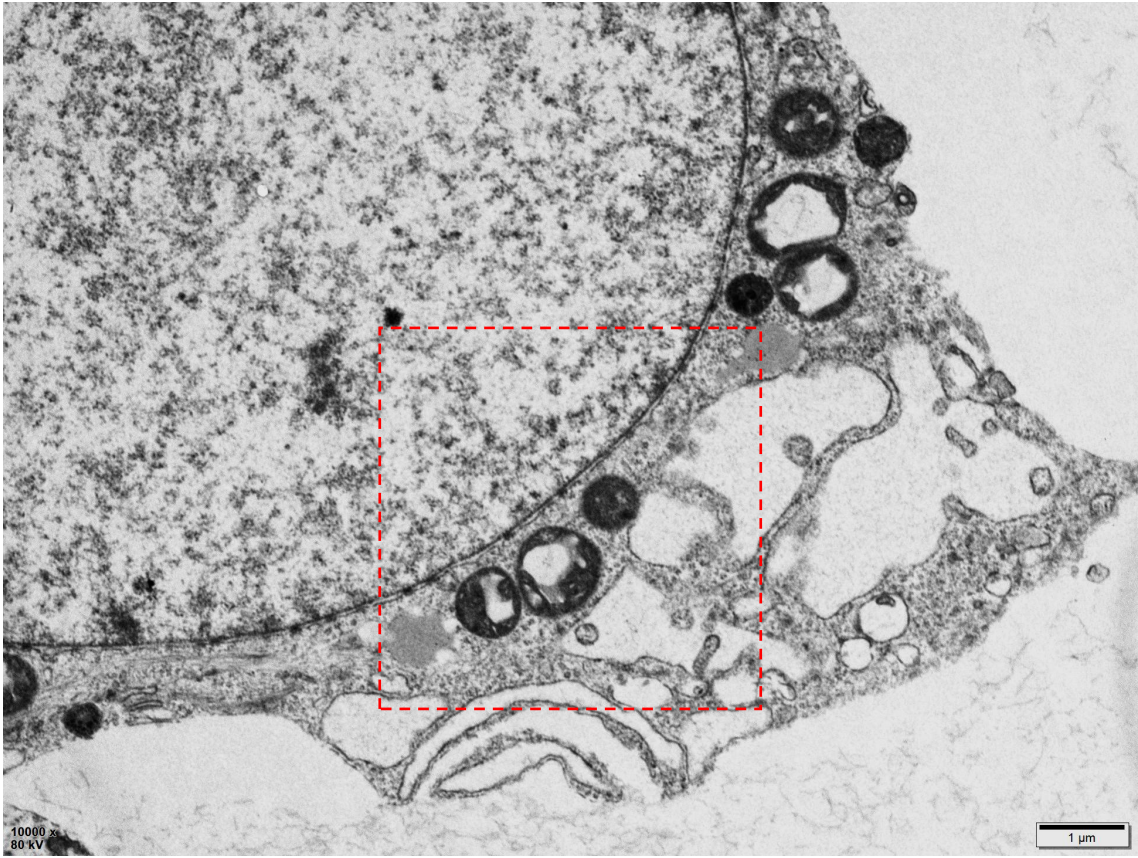

ASZ1+PIWIL2+TDRD1

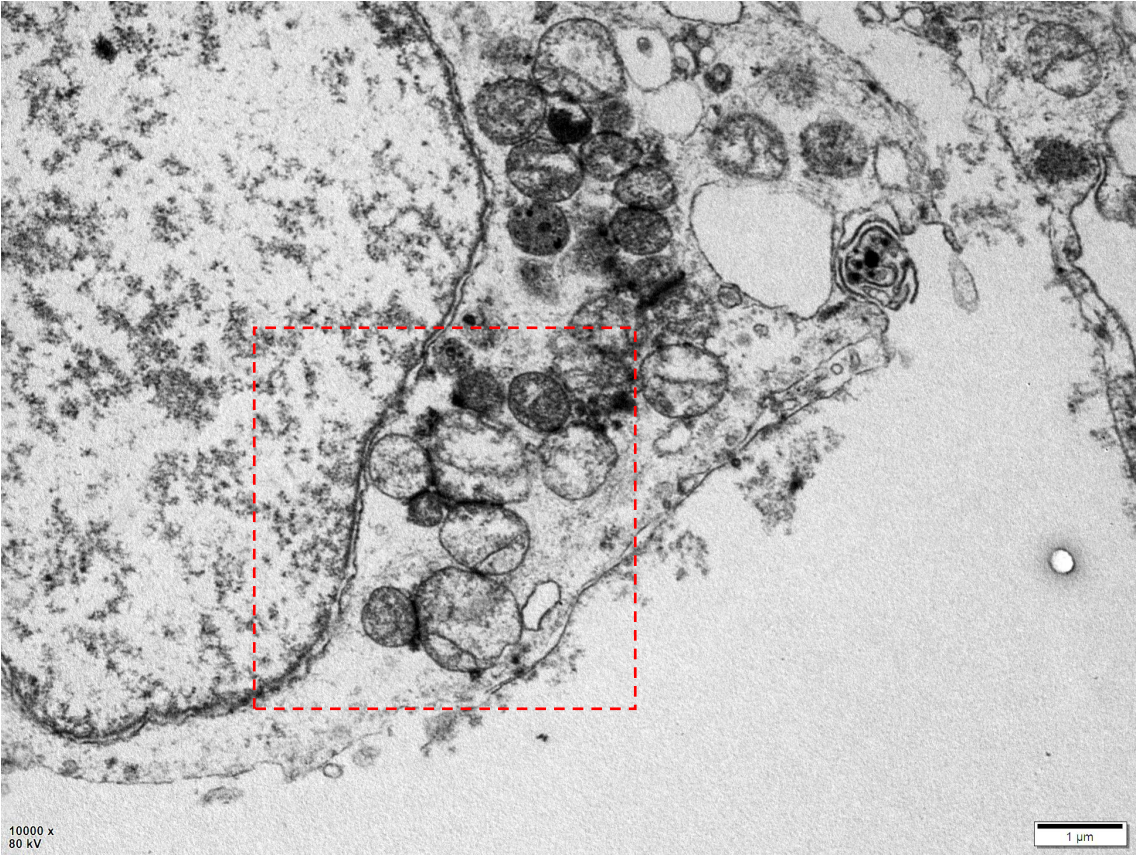

Supplement: Supplementary file 6 — Source data Fig. 1 [file 44318_2025_579_MOESM6_ESM.zip › Figure 1/1H/Figure 1H.pdf]

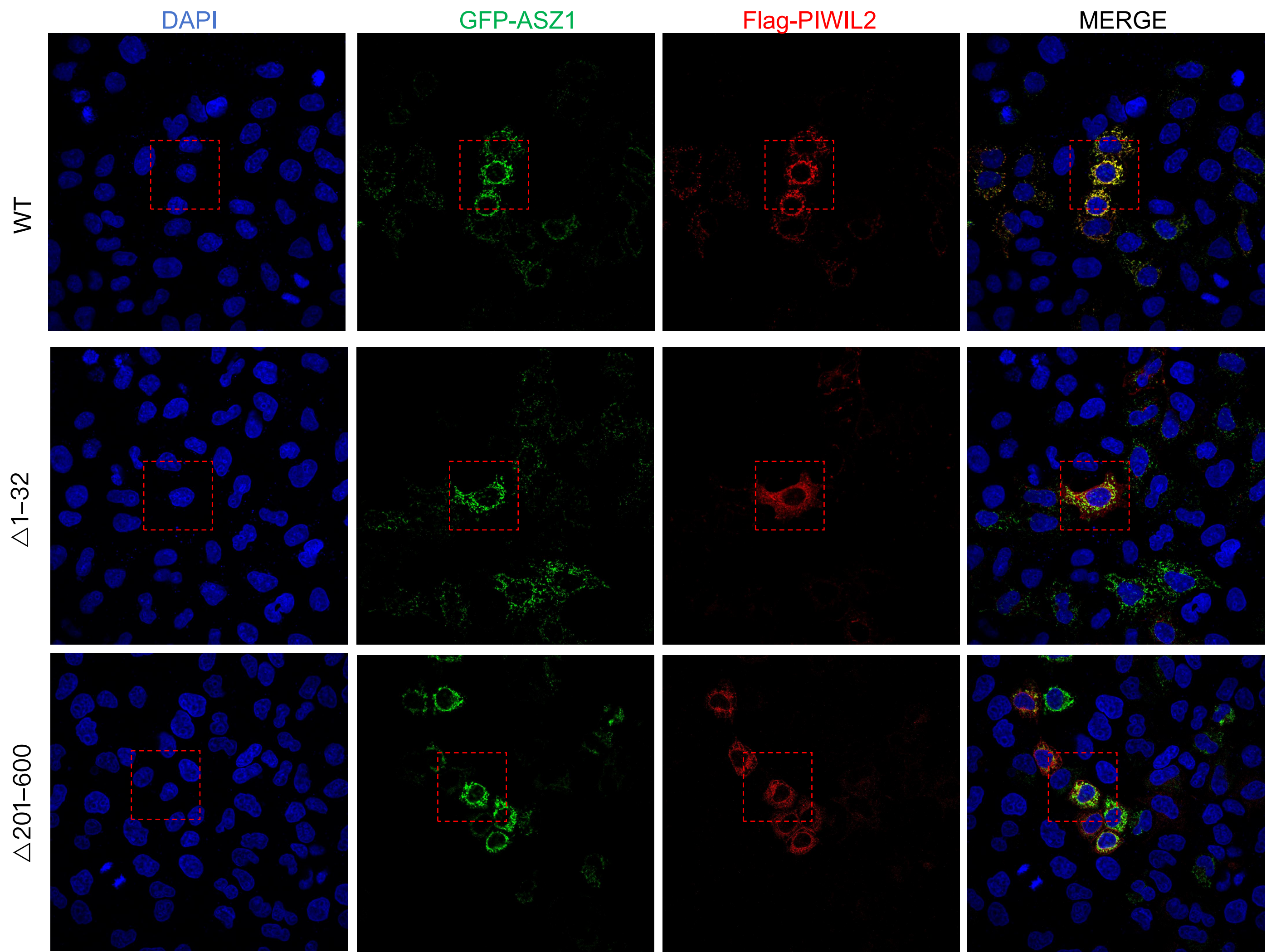

Supplement: Supplementary file 7 — Source data Fig. 2 [file 44318_2025_579_MOESM7_ESM.zip › Figure 2/2E/Figure 2E.pdf]

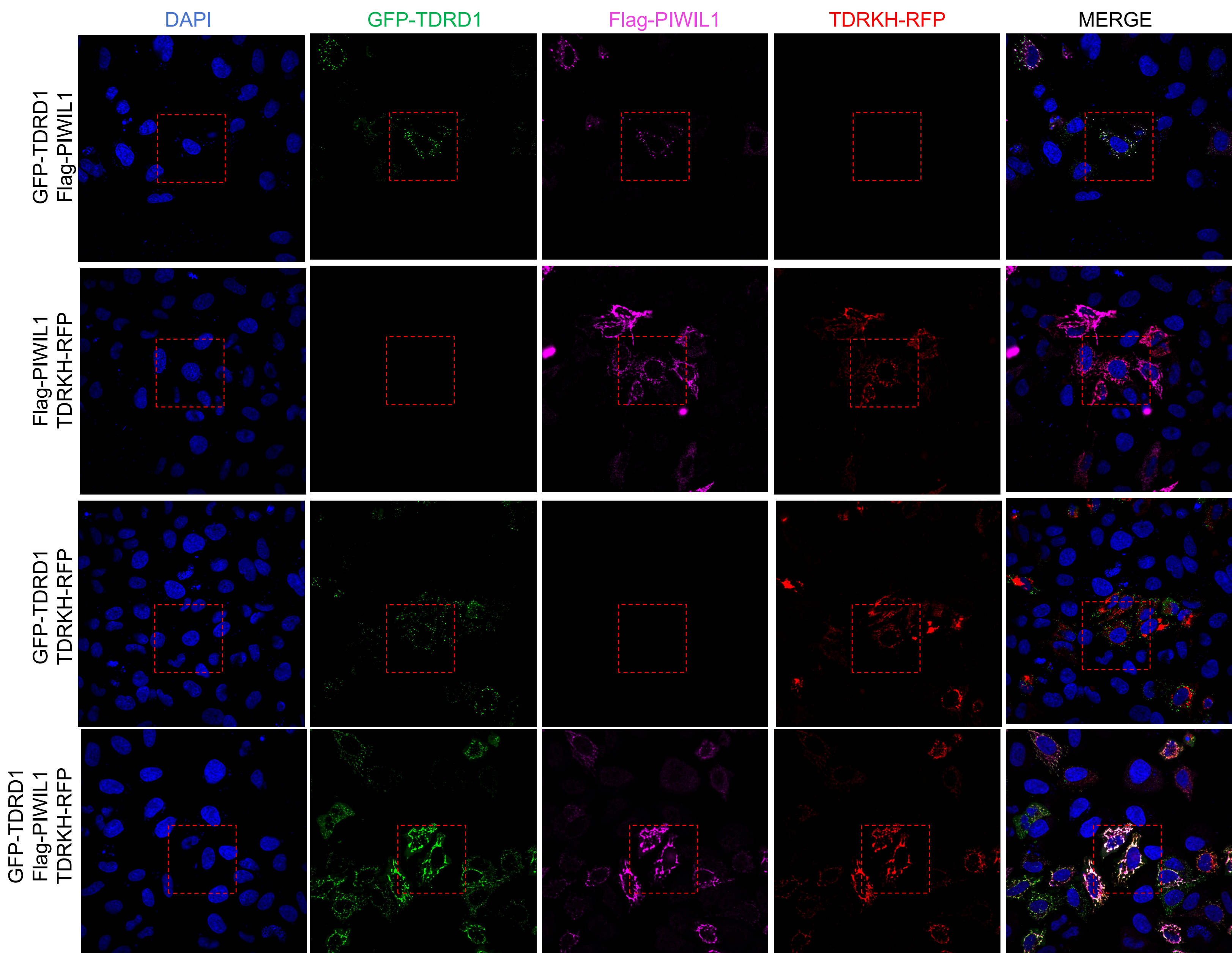

Supplement: Supplementary file 8 — Source data Fig. 3 [file 44318_2025_579_MOESM8_ESM.zip › Figure 3/3F/Figure 3F.pdf]

DAPI

GFP

Flag-PIWIL1

TDRKH-RFP

MERGE

GFP-TDRD1  
Flag-PIWIL1  
TDRKH-RFP

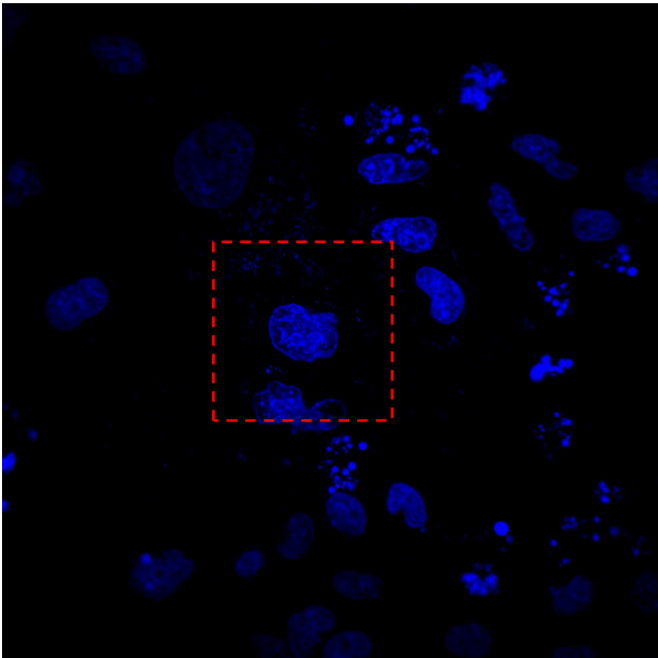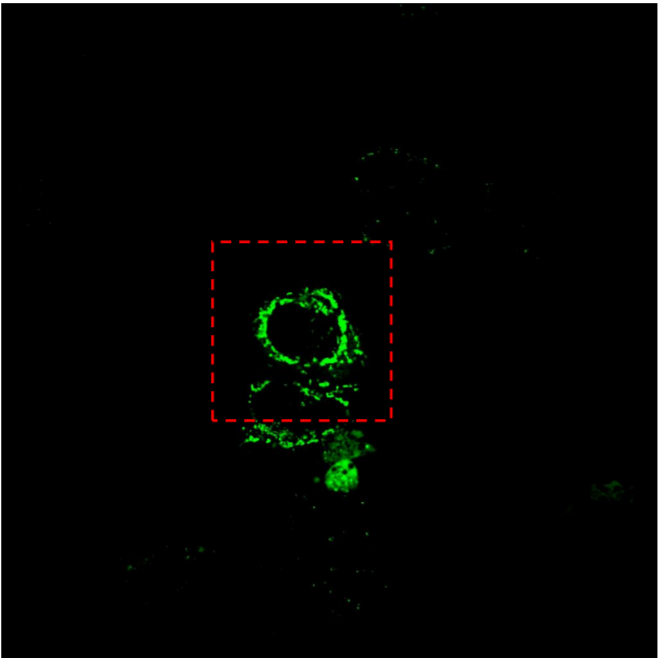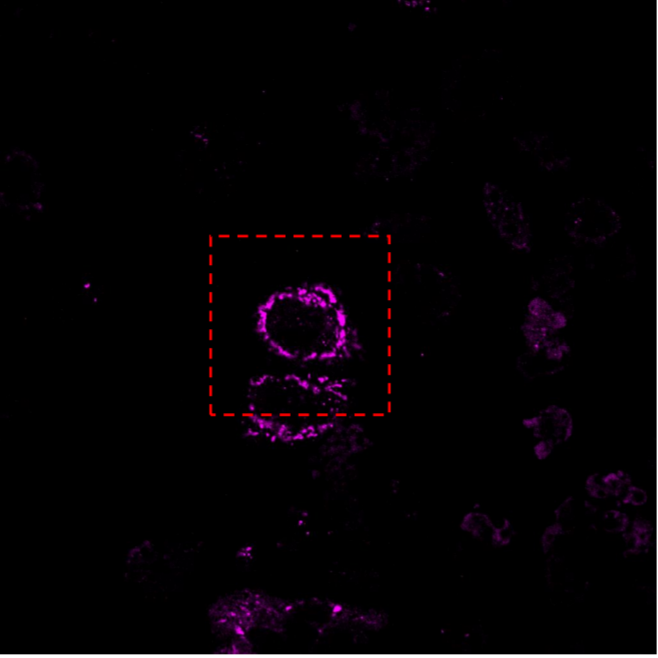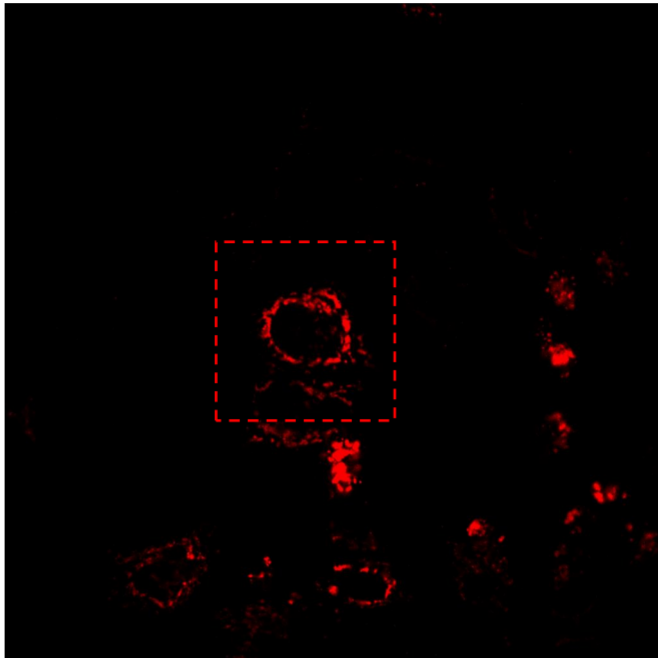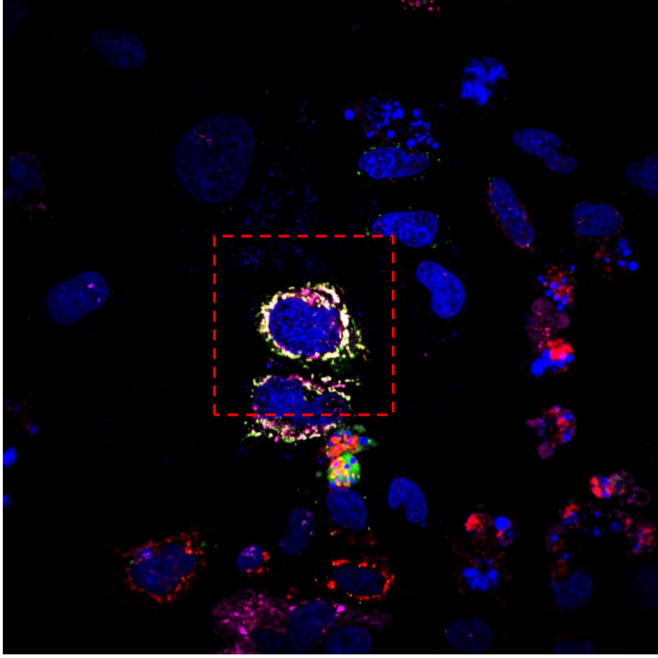

GFP-TDRD1-GS  
Flag-PIWIL1  
TDRKH-RFP

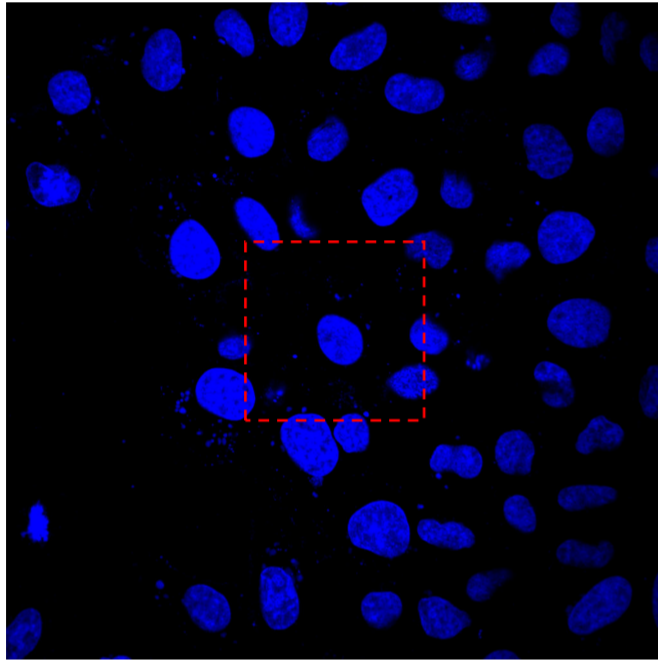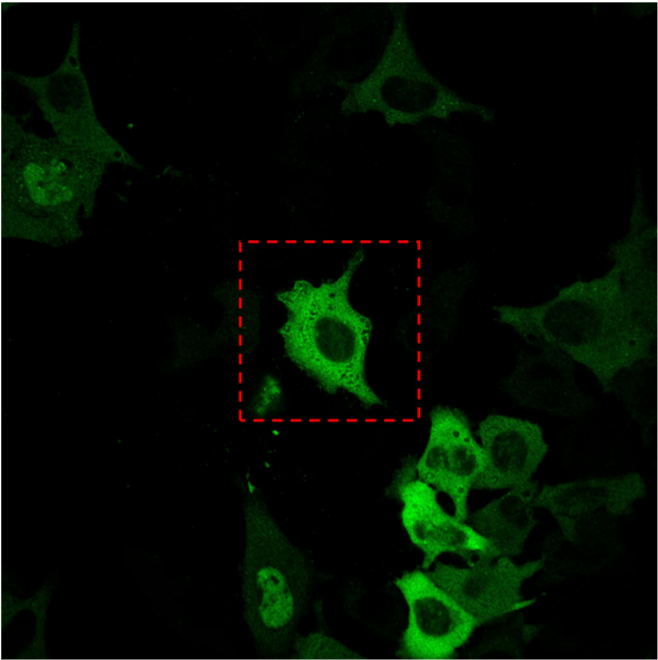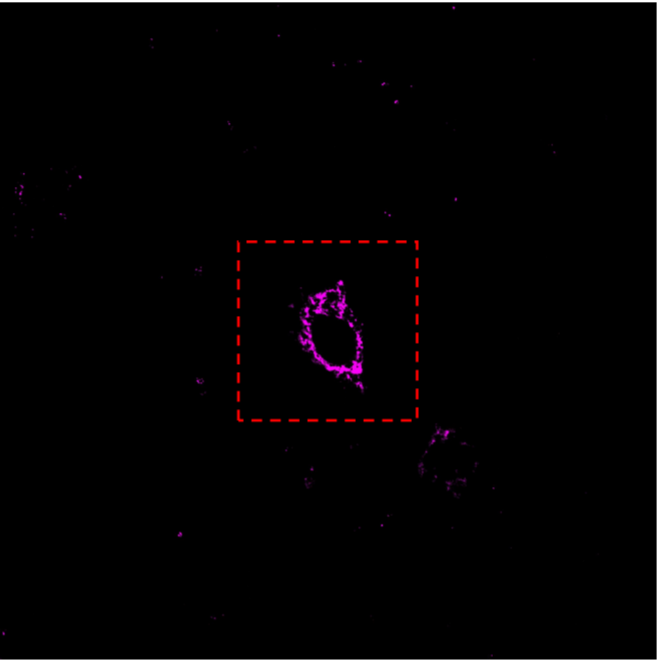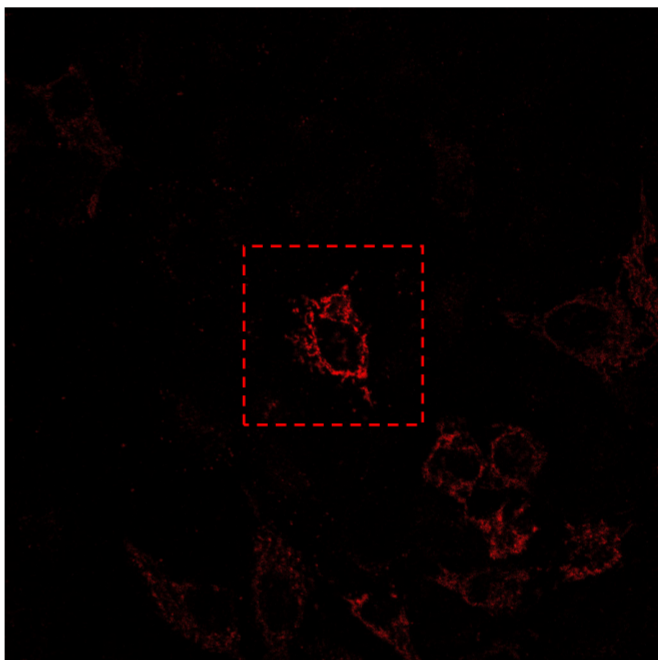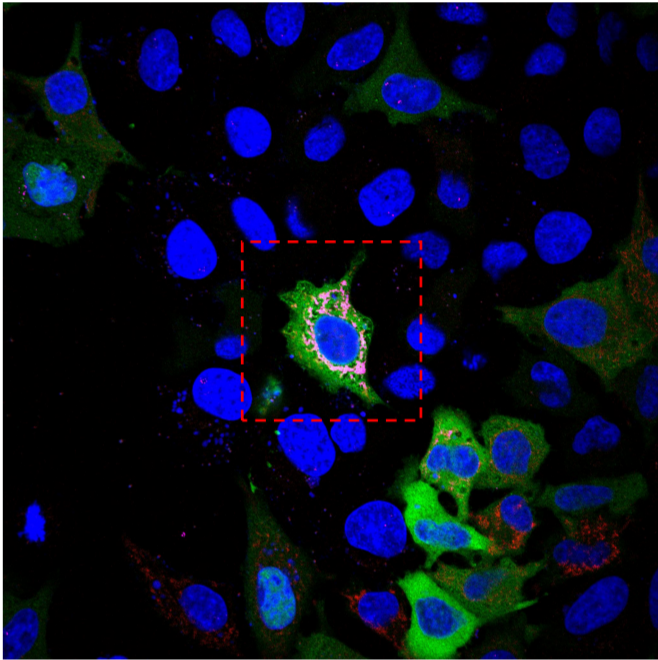

Supplement: Supplementary file 8 — Source data Fig. 3 [file 44318_2025_579_MOESM8_ESM.zip › Figure 3/3G/Figure 3G.pdf]

Negative Control

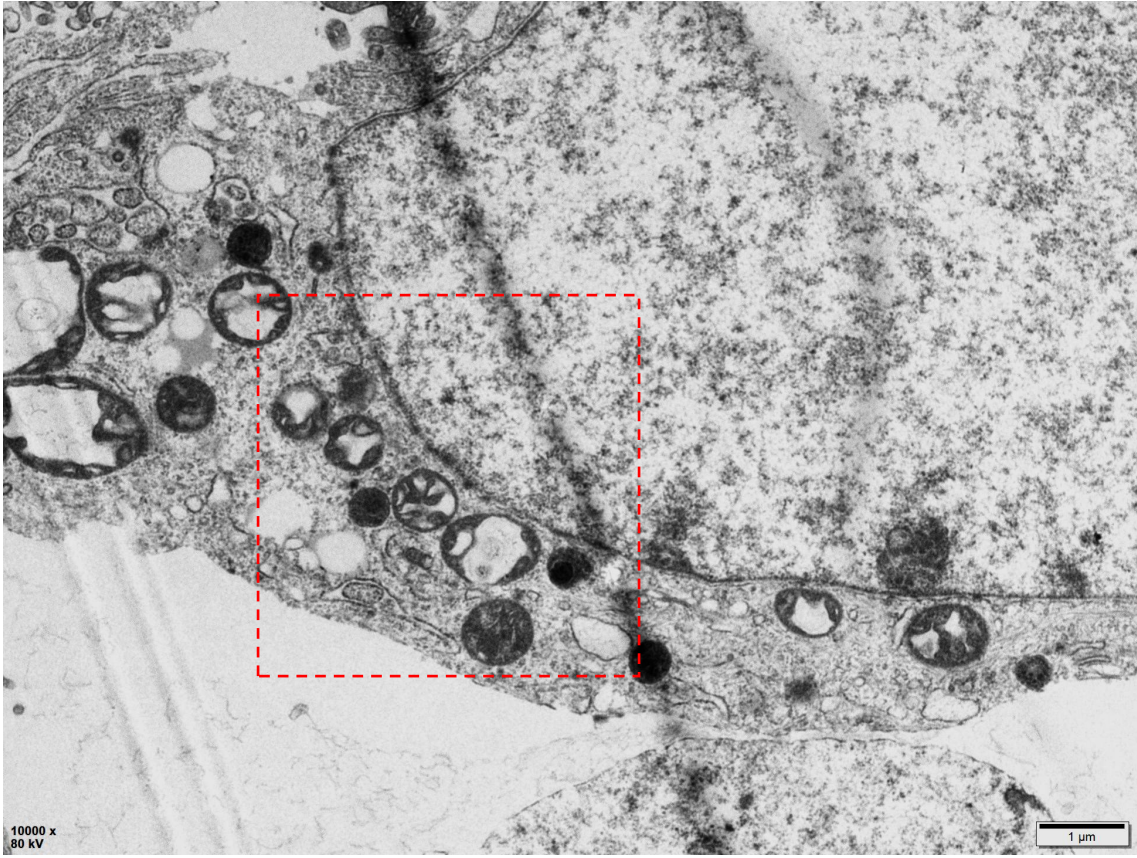

TDRKH+PIWIL1+TDRD1

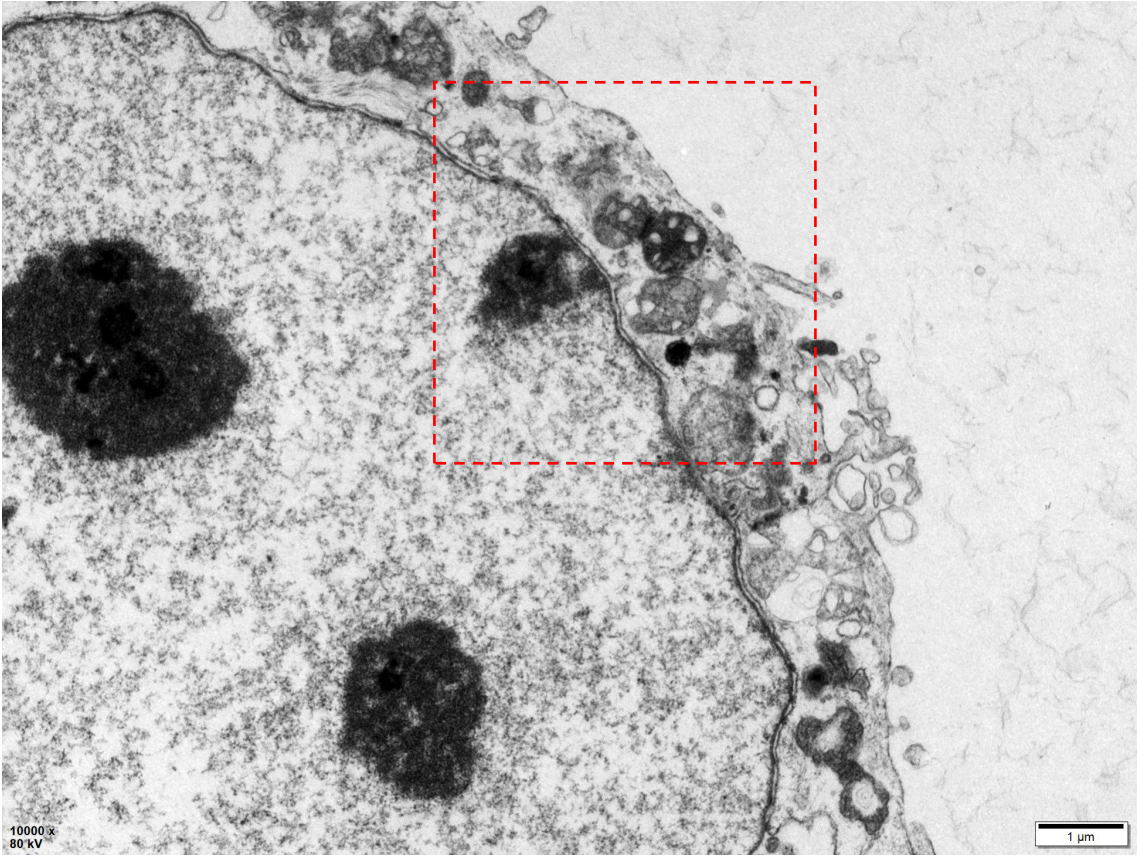

Supplement: Supplementary file 8 — Source data Fig. 3 [file 44318_2025_579_MOESM8_ESM.zip › Figure 3/3H/Figure 3H.pdf]

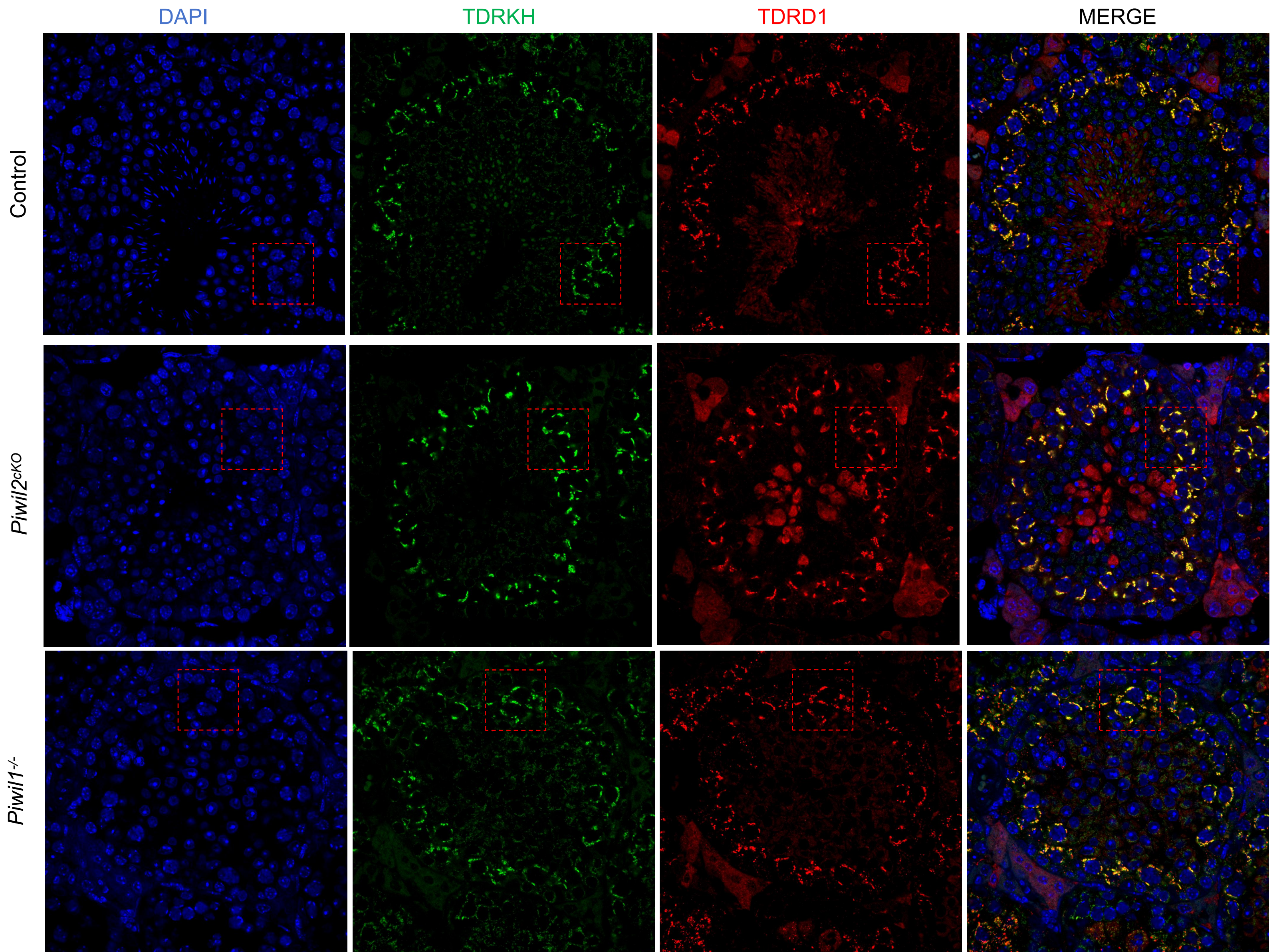

Supplement: Supplementary file 9 — Source data Fig. 4 [file 44318_2025_579_MOESM9_ESM.zip › Figure 4/4A/Figure 4A.pdf]

Pachytene Spermatocytes (Adult)

Control

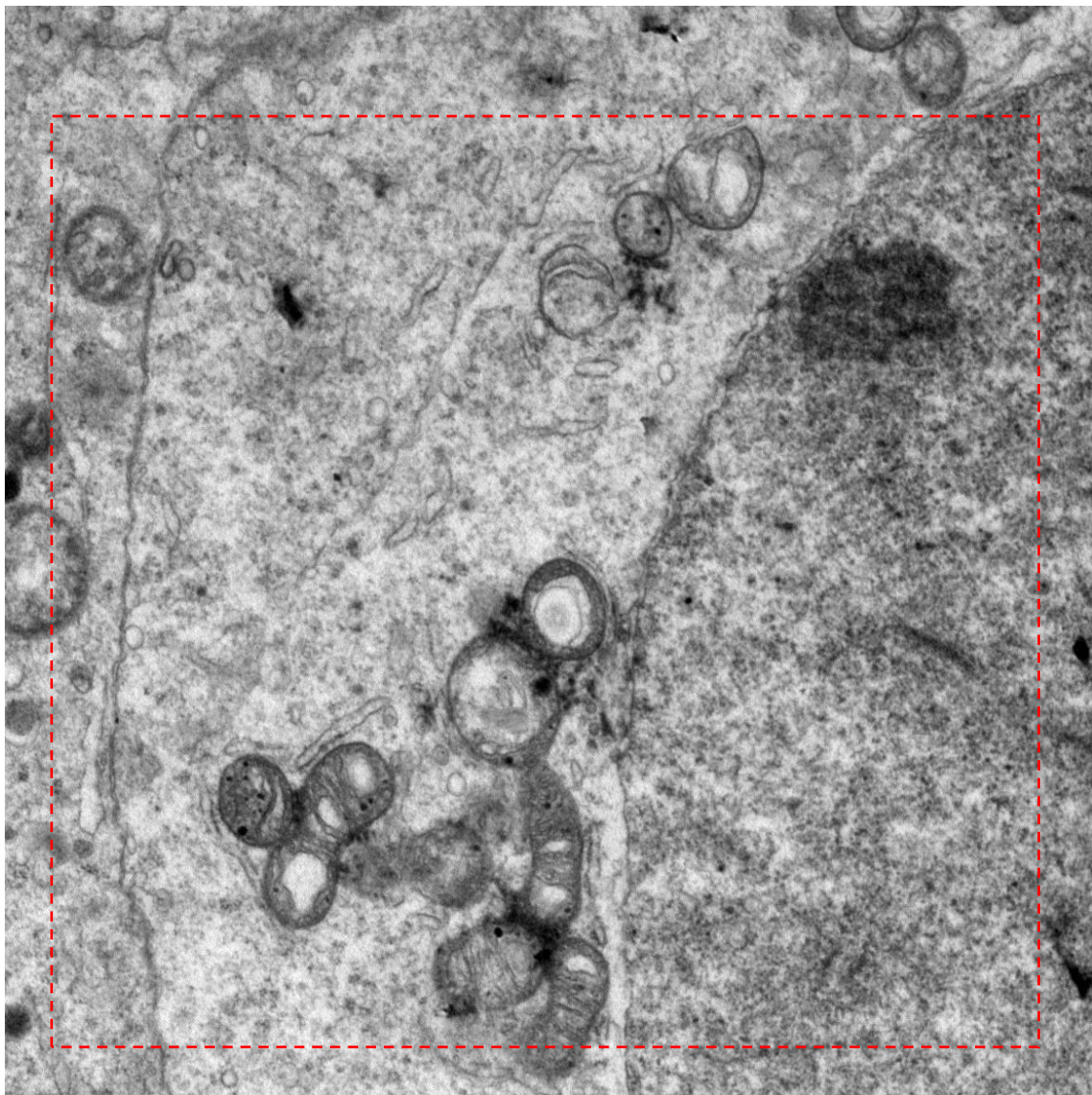

*Piwi*2<sup>cko</sup>

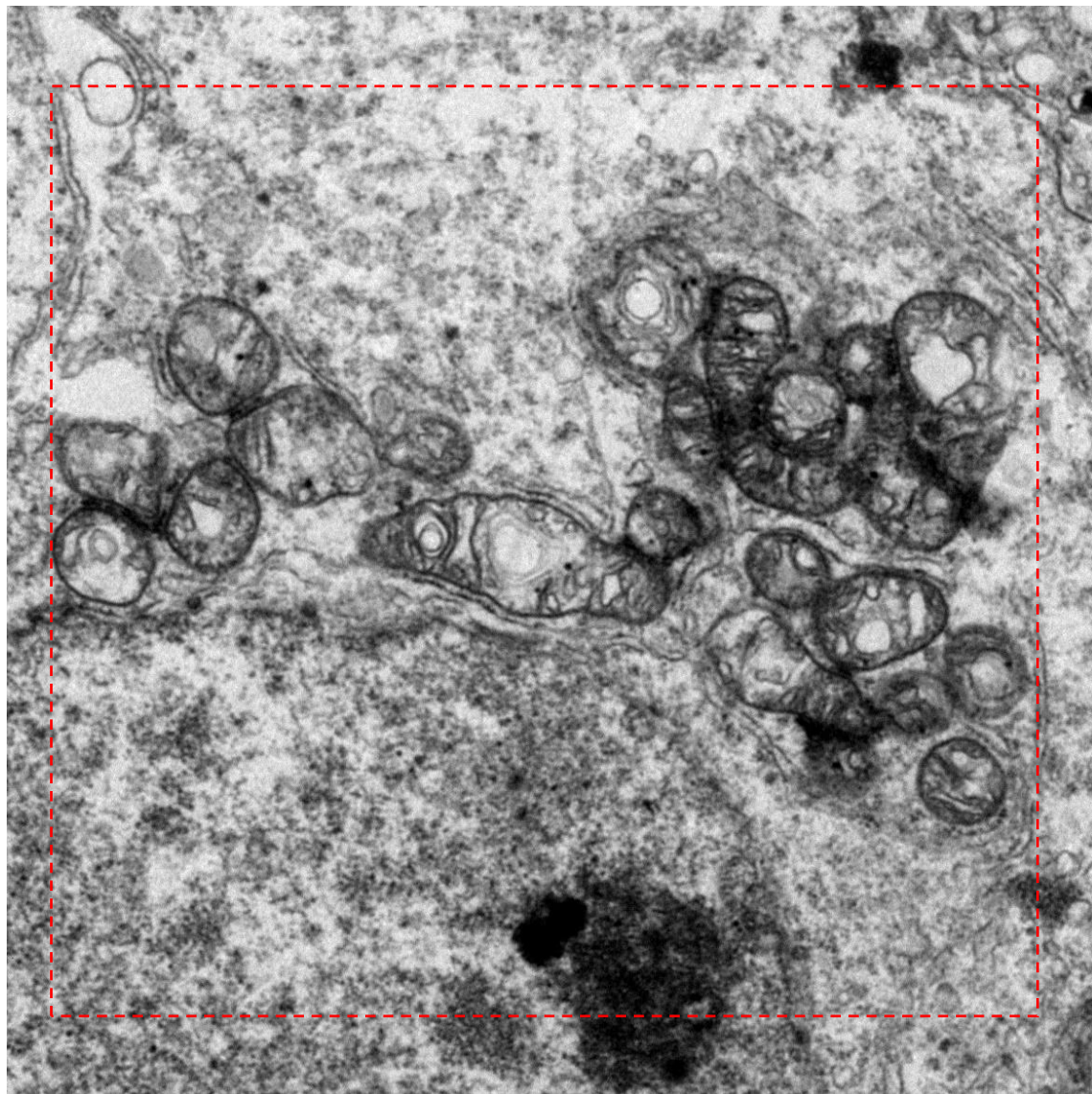

Supplement: Supplementary file 9 — Source data Fig. 4 [file 44318_2025_579_MOESM9_ESM.zip › Figure 4/4B/Figure 4B.pdf]

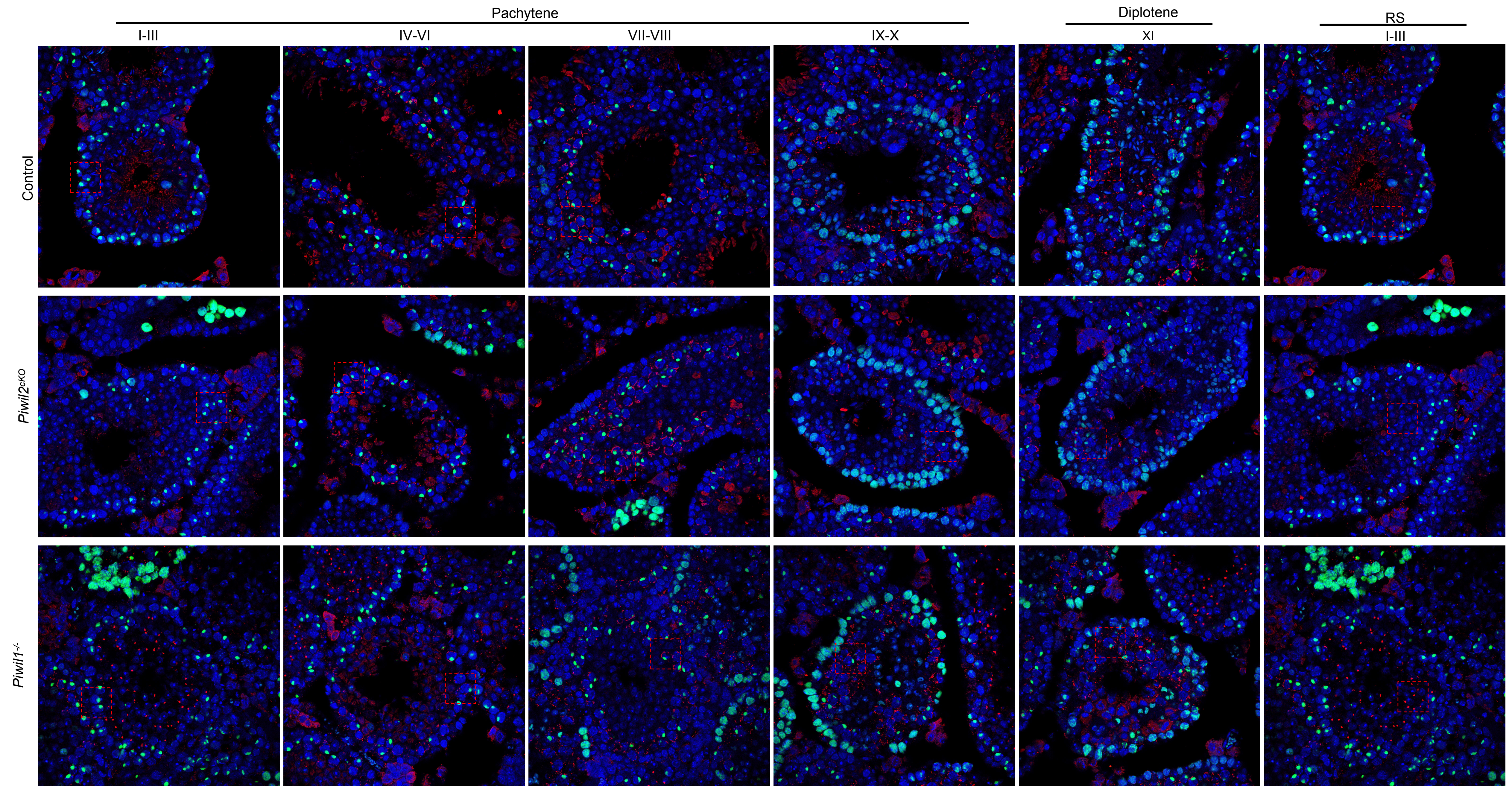

Supplement: Supplementary file 9 — Source data Fig. 4 [file 44318_2025_579_MOESM9_ESM.zip › Figure 4/4C/Figure 4C.pdf]

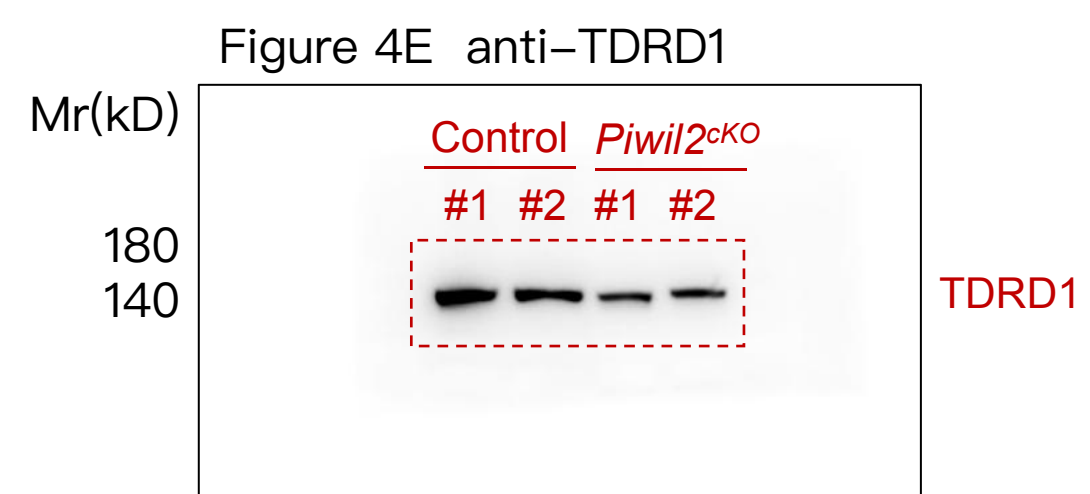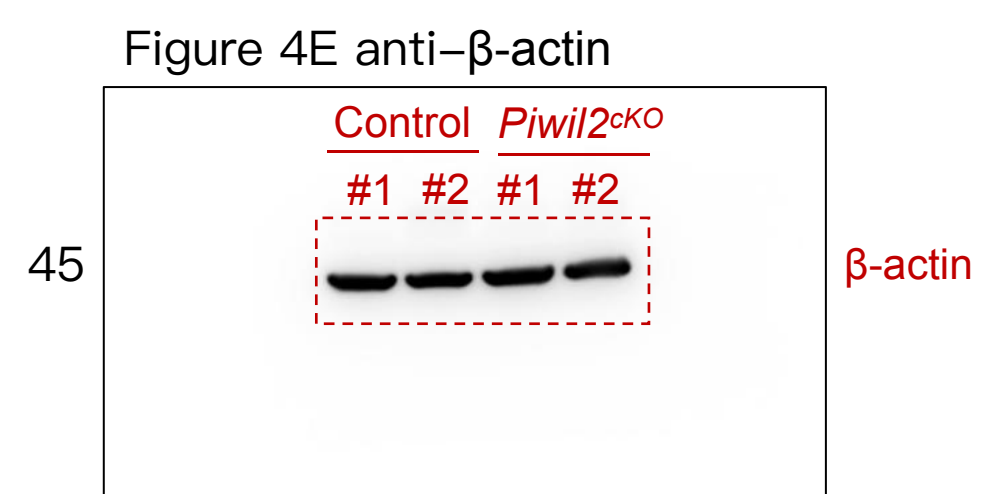

Supplement: Supplementary file 9 — Source data Fig. 4 [file 44318_2025_579_MOESM9_ESM.zip › Figure 4/4D/Figure 4D.pdf]

Figure 4F anti-PIWIL1

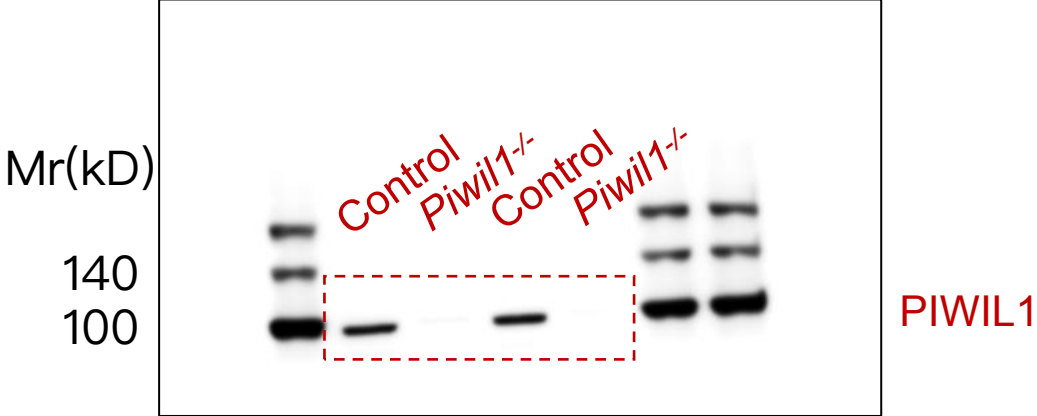

Figure 4F anti-TDRD1

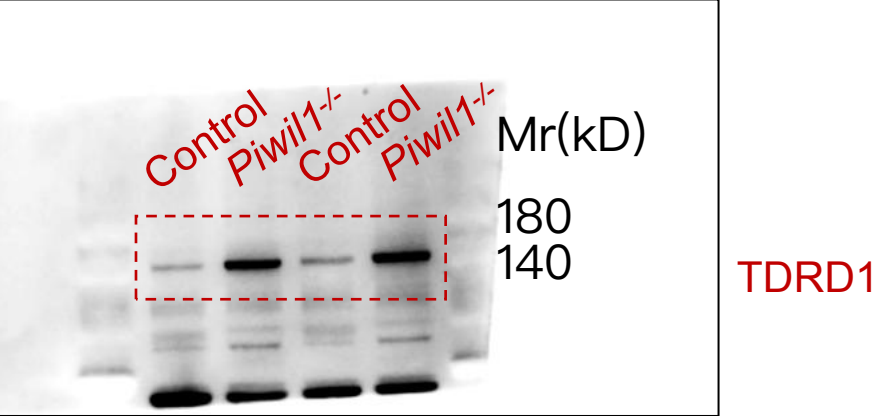

Figure 4F anti-PIWIL2

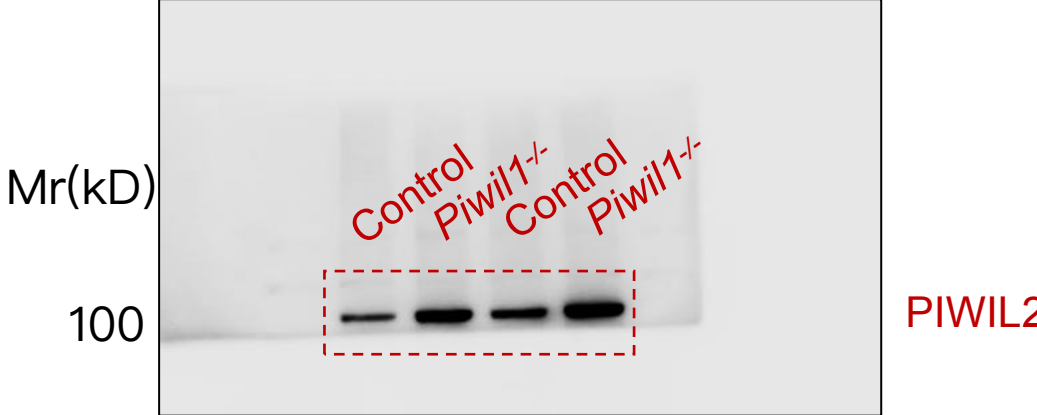

Figure 4F anti-β-actin

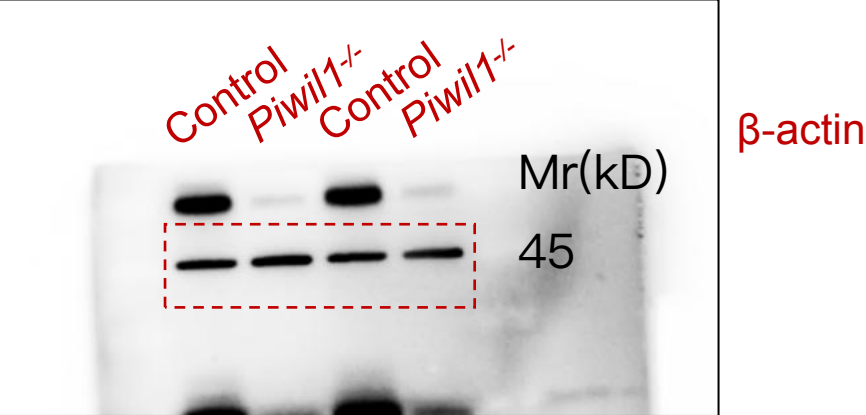

Supplement: Supplementary file 9 — Source data Fig. 4 [file 44318_2025_579_MOESM9_ESM.zip › Figure 4/4E/Figure 4E.pdf]

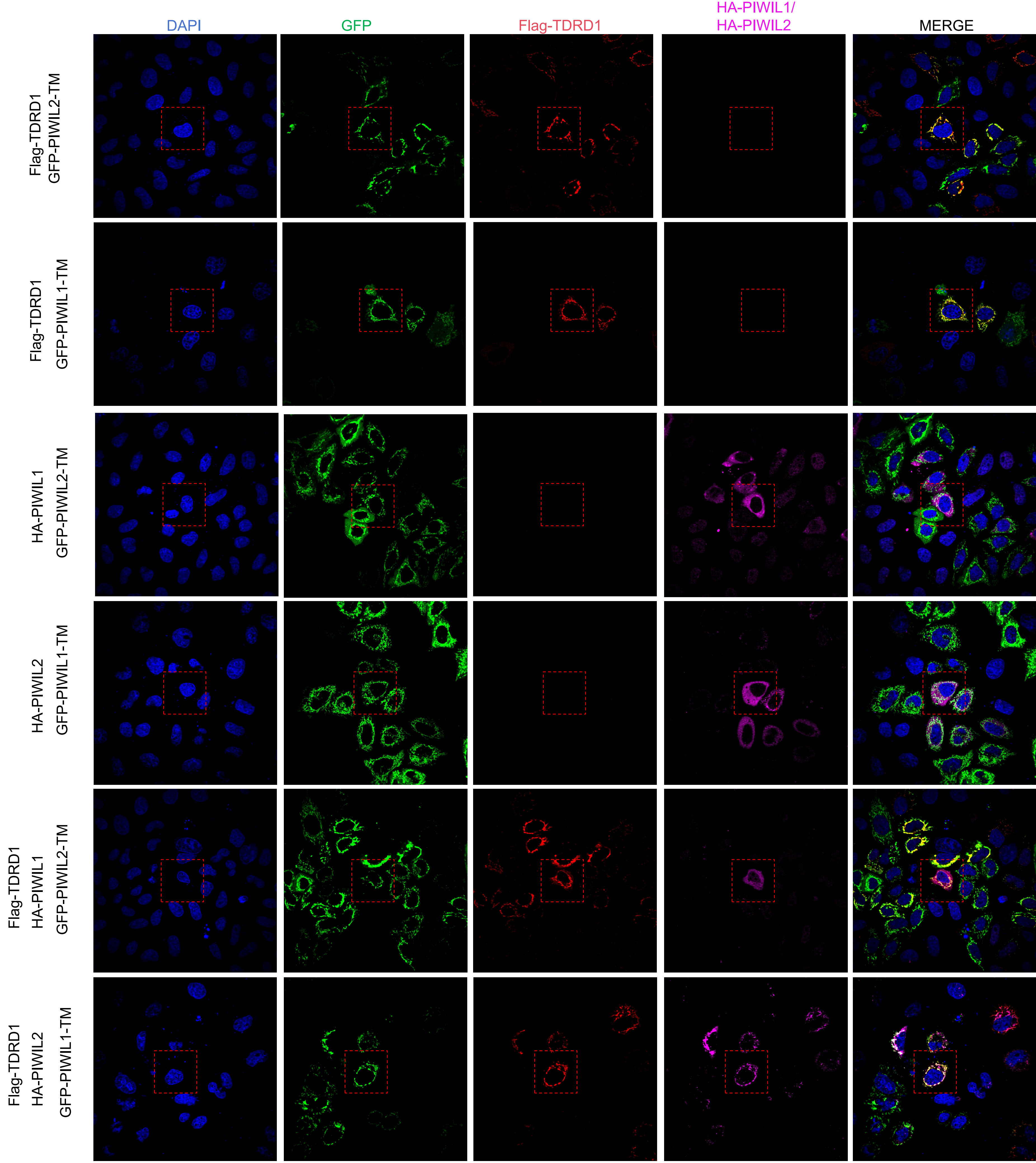

Supplement: Supplementary file 9 — Source data Fig. 4 [file 44318_2025_579_MOESM9_ESM.zip › Figure 4/4G/Figure 4G.pdf]

mCherry-ASZ1

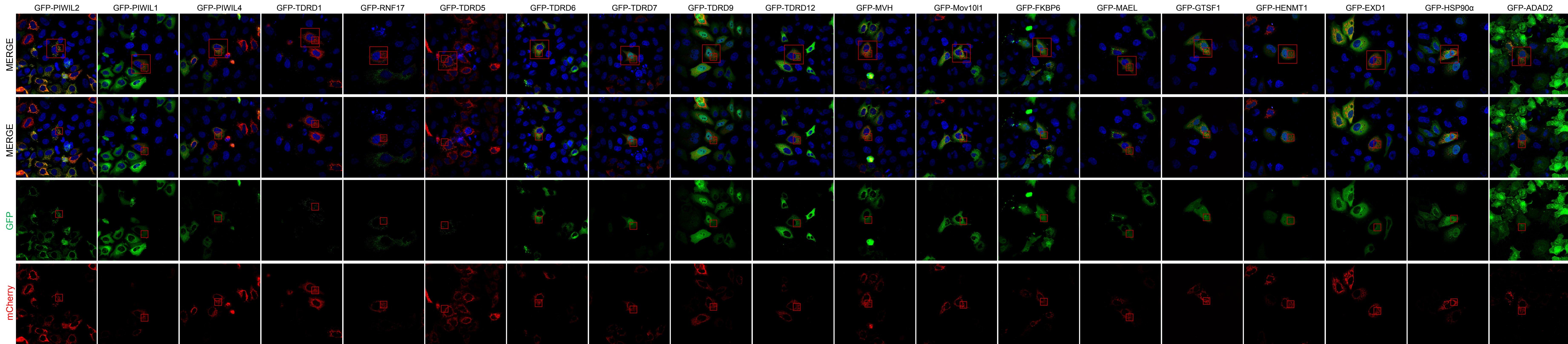

Supplement: Supplementary file 10 — Source data Fig. 5 [file 44318_2025_579_MOESM10_ESM.zip › Figure 5/5A/Figure 5A.pdf]

TDRKH-RFP

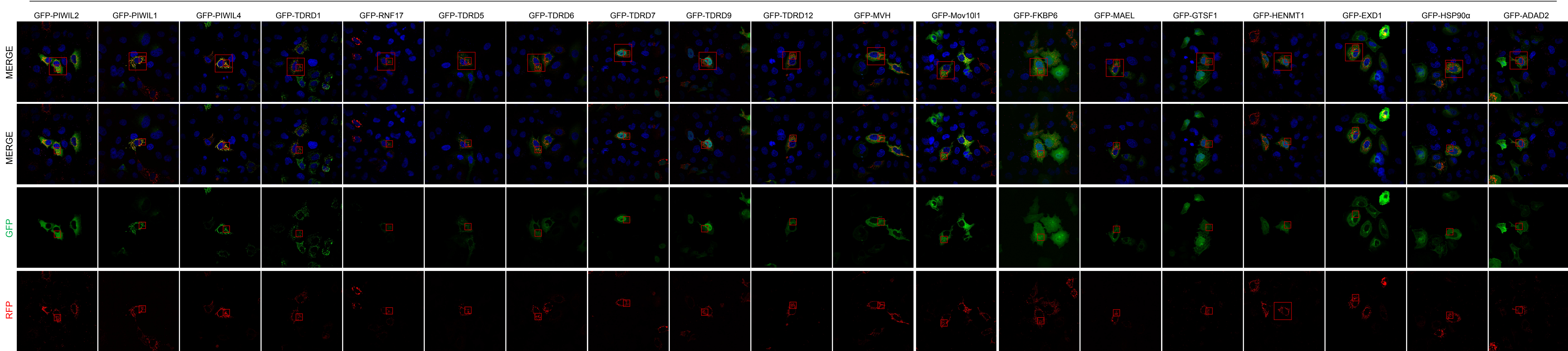

Supplement: Supplementary file 10 — Source data Fig. 5 [file 44318_2025_579_MOESM10_ESM.zip › Figure 5/5B/Figure 5B.pdf]

mCherry-TDRD1

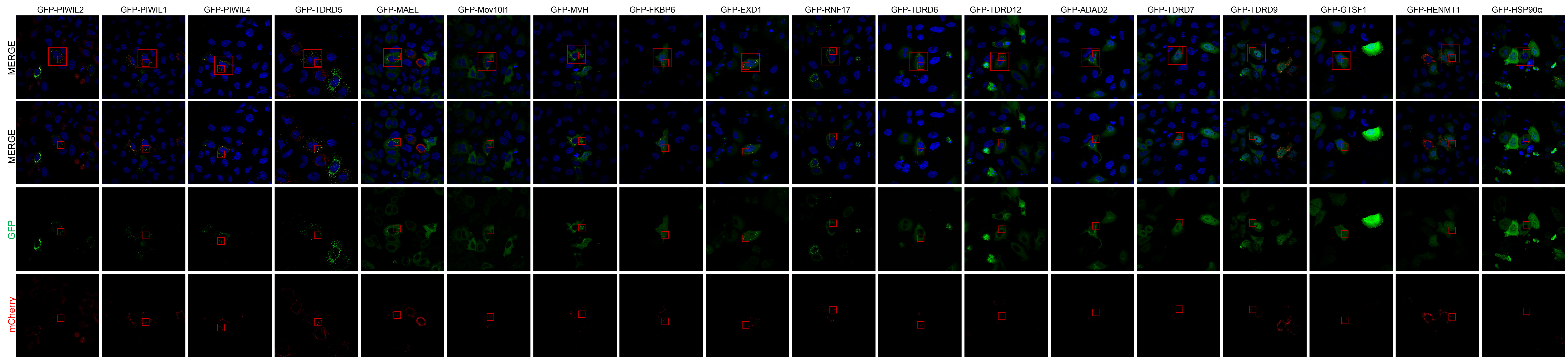

Supplement: Supplementary file 10 — Source data Fig. 5 [file 44318_2025_579_MOESM10_ESM.zip › Figure 5/5C/Figure 5C.pdf]

Control

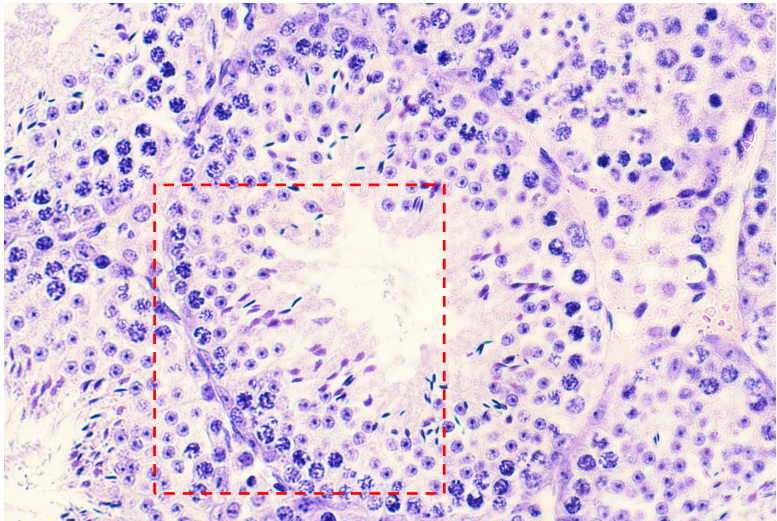

*Tdrd1*<sup>cko</sup>

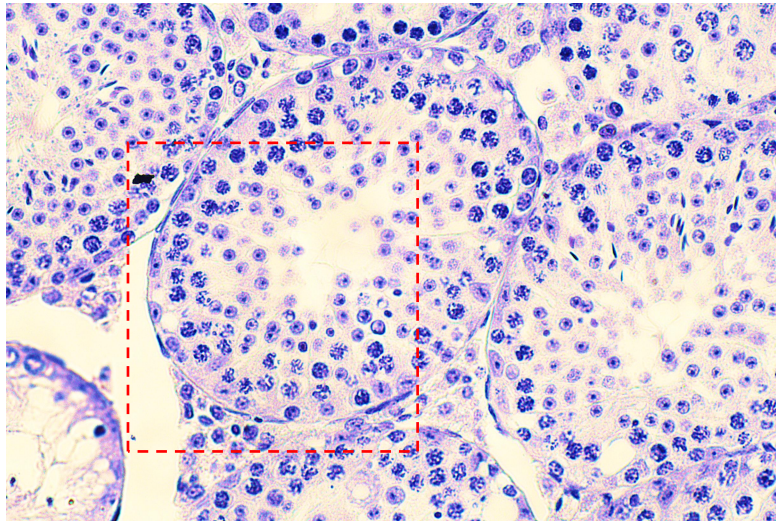

Supplement: Supplementary file 11 — Source data Fig. 6 [file 44318_2025_579_MOESM11_ESM.zip › Figure 6/6B/Figure 6B.pdf]

TDRKH/rH2AX/DAPI

Control

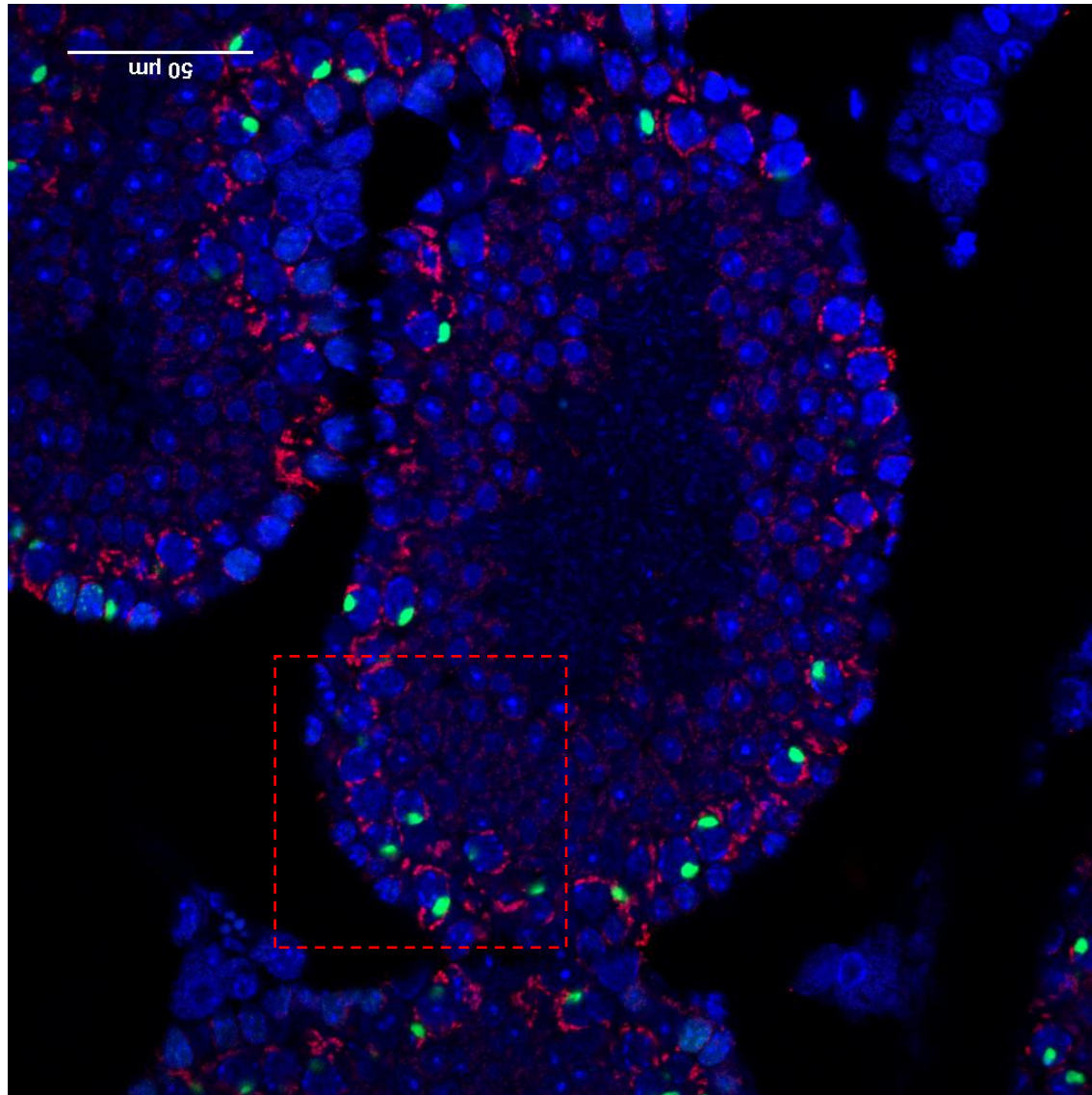

*Tdrd1*<sup>CKO</sup>

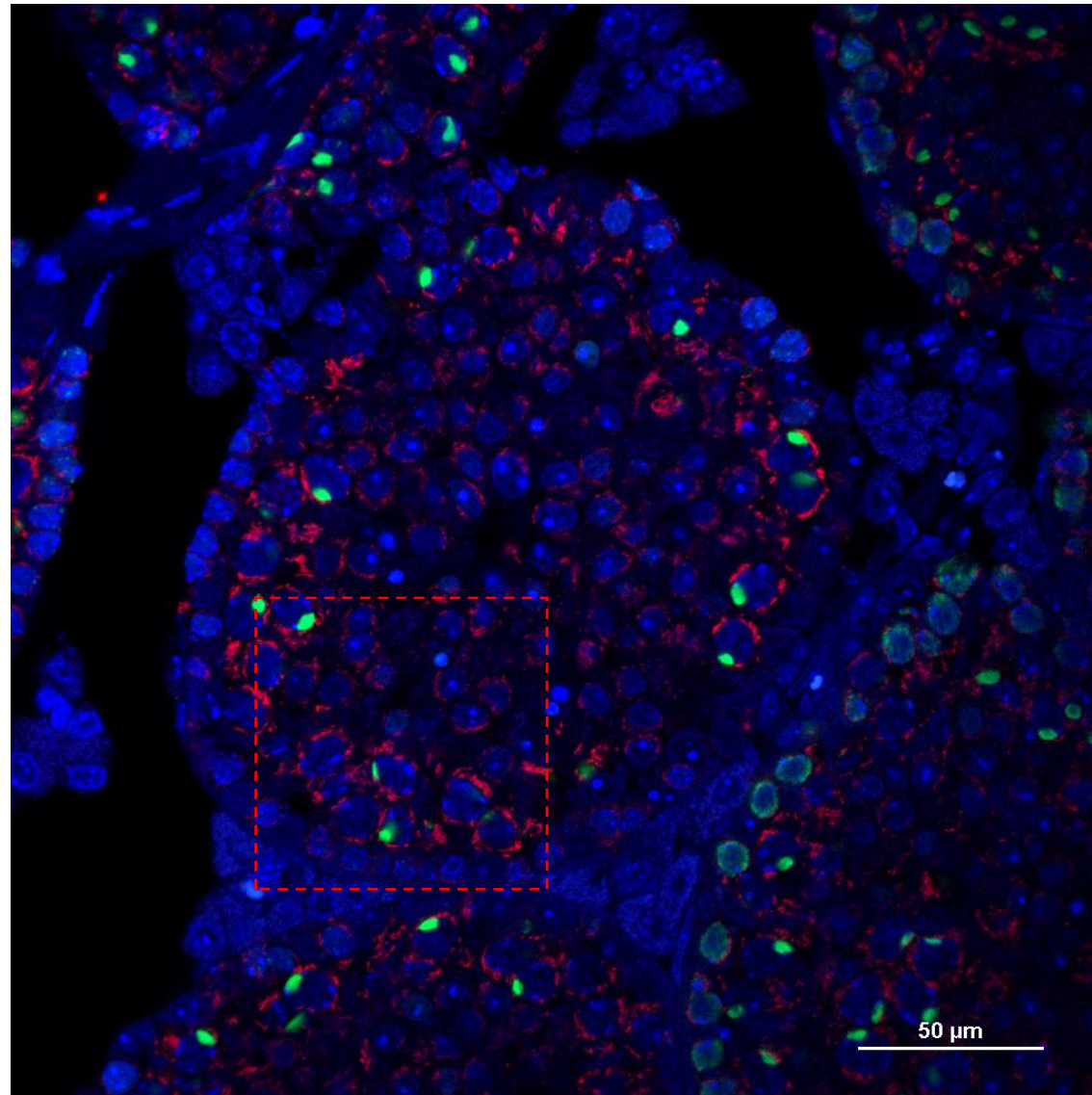

Supplement: Supplementary file 11 — Source data Fig. 6 [file 44318_2025_579_MOESM11_ESM.zip › Figure 6/6C/Figure 6C.pdf]

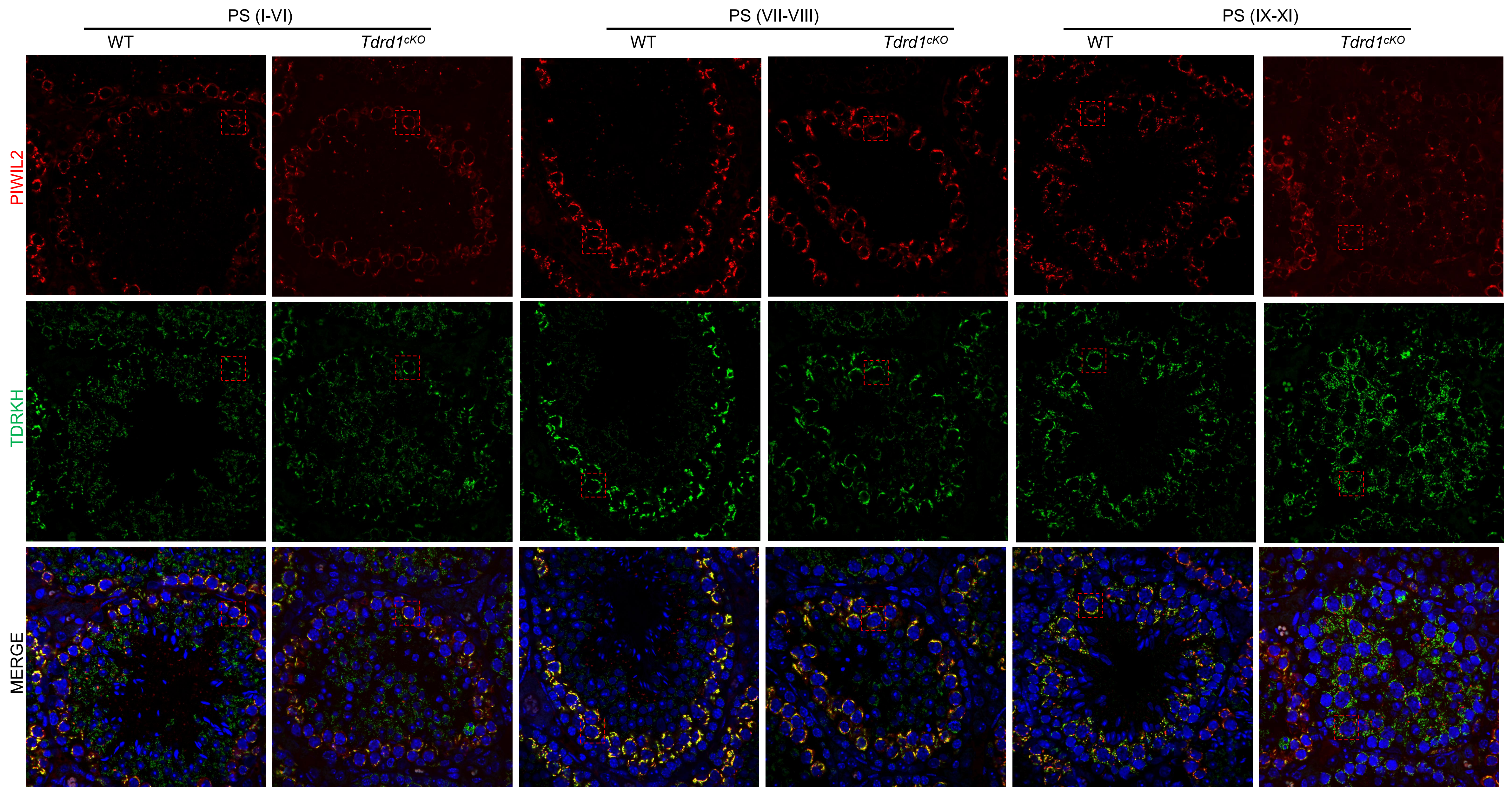

Supplement: Supplementary file 11 — Source data Fig. 6 [file 44318_2025_579_MOESM11_ESM.zip › Figure 6/6D/Figure 6D.pdf]

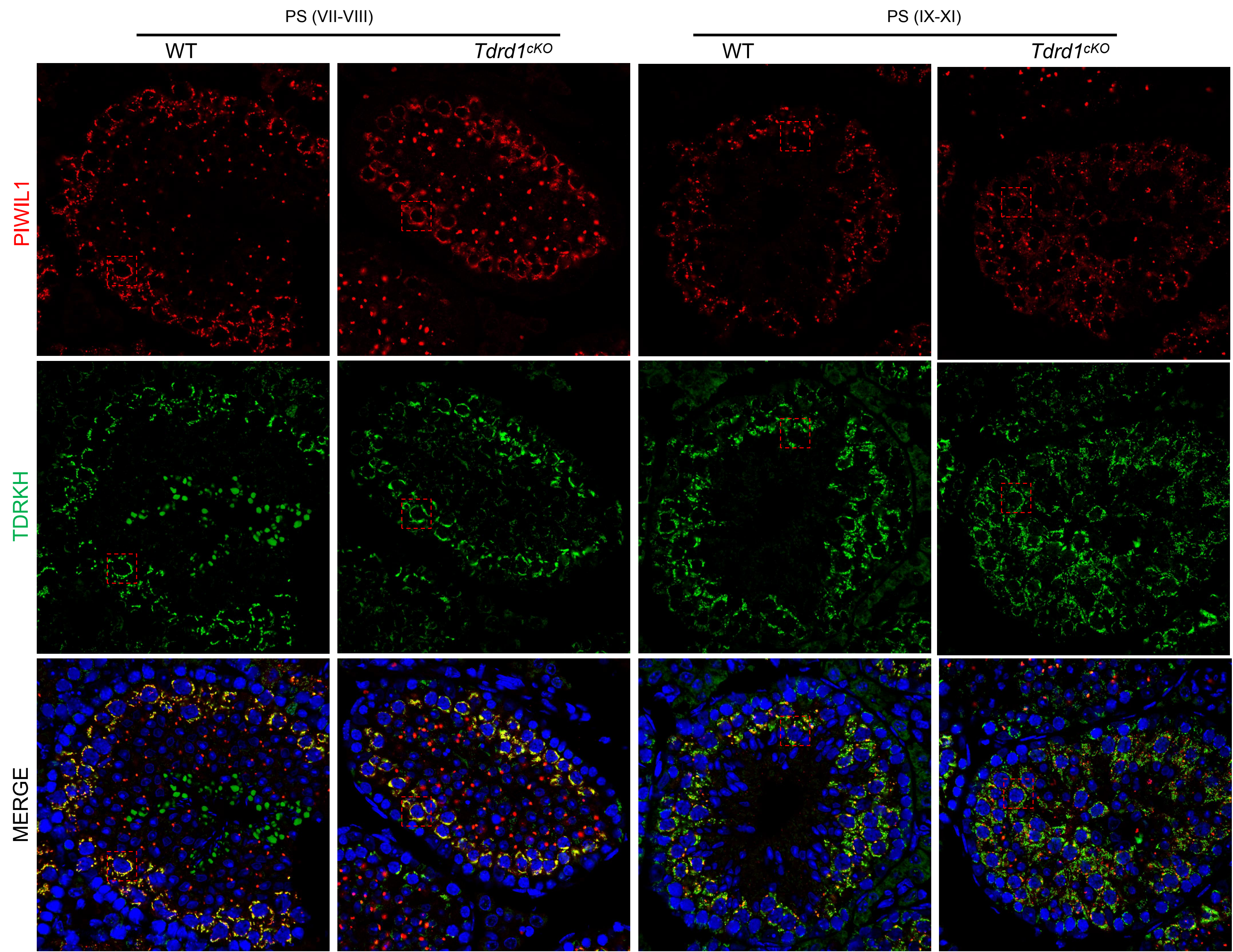

Supplement: Supplementary file 11 — Source data Fig. 6 [file 44318_2025_579_MOESM11_ESM.zip › Figure 6/6E/Figure 6E.pdf]

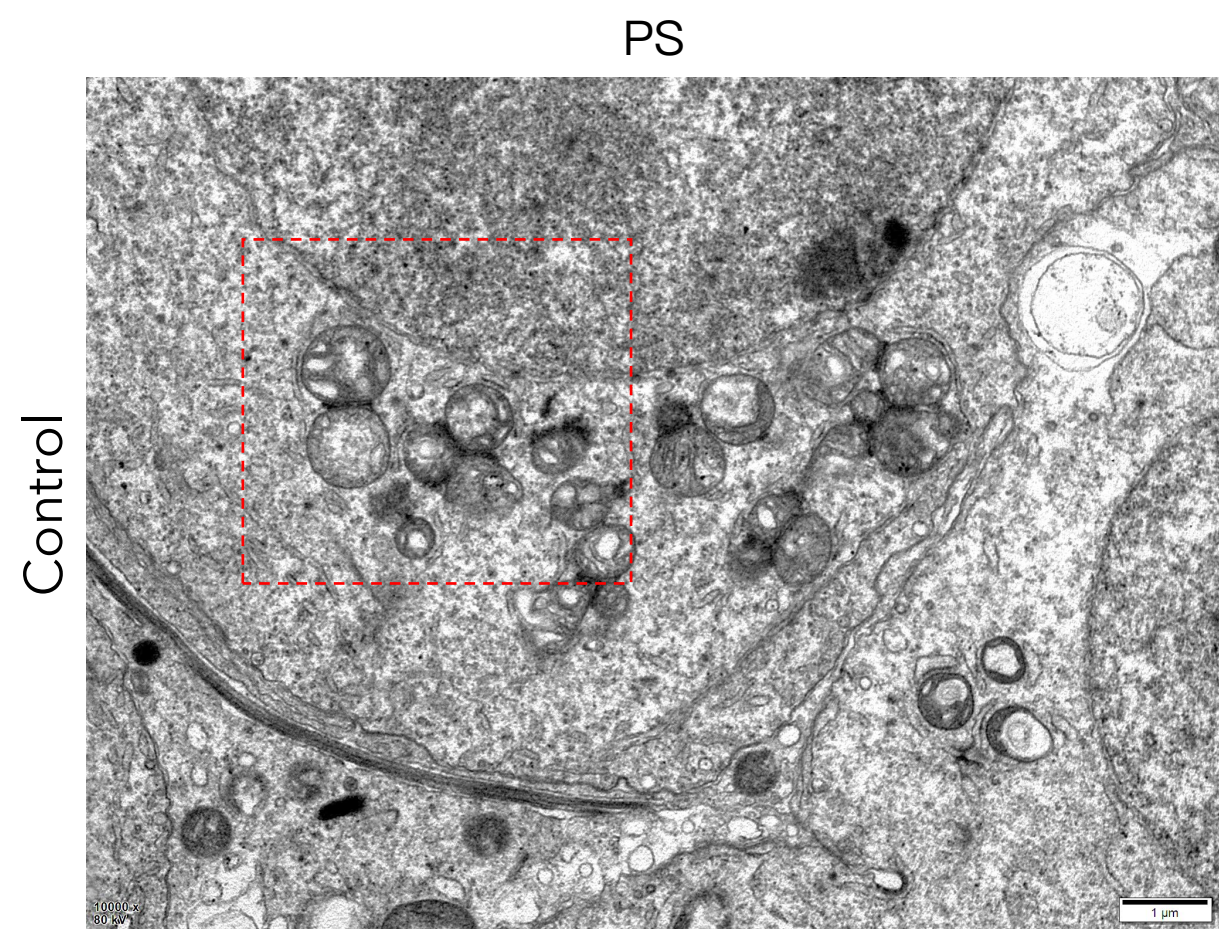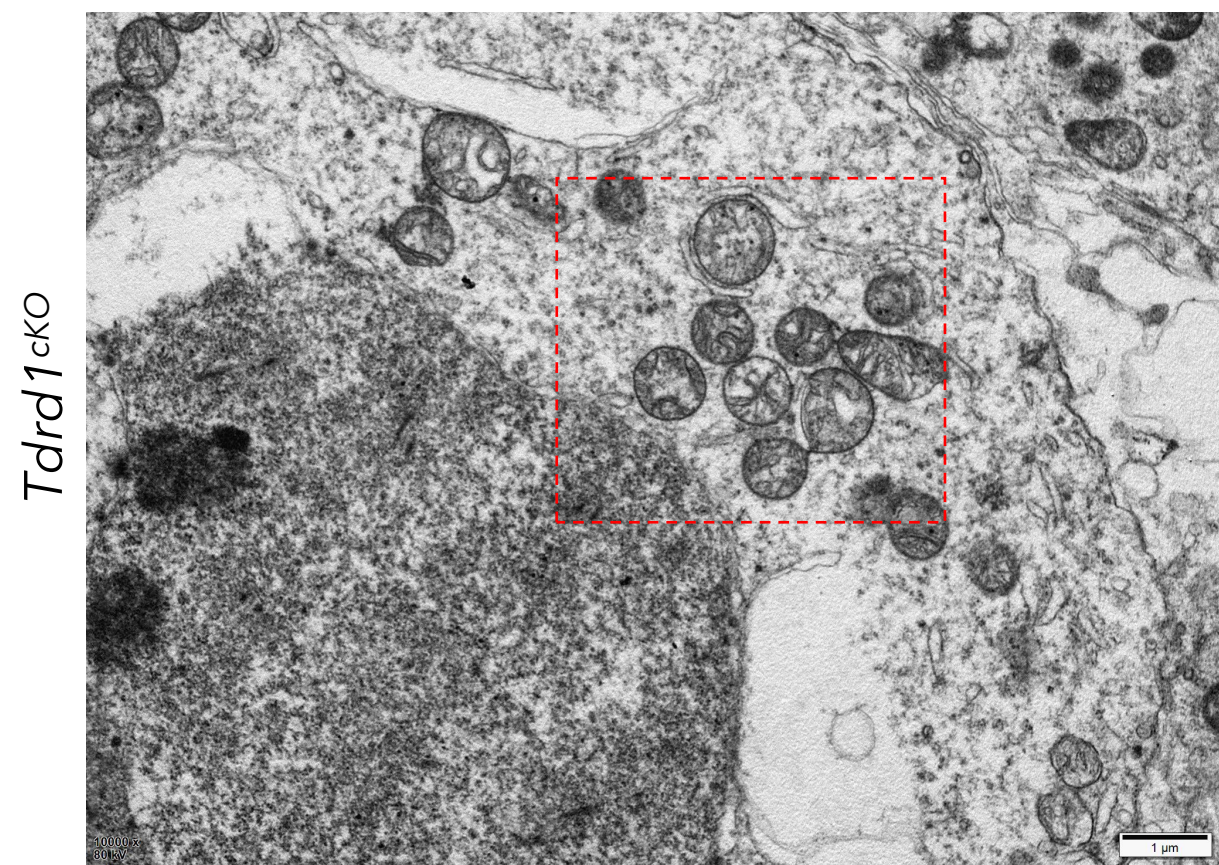

Supplement: Supplementary file 11 — Source data Fig. 6 [file 44318_2025_579_MOESM11_ESM.zip › Figure 6/6F/Figure 6F.pdf]

RS

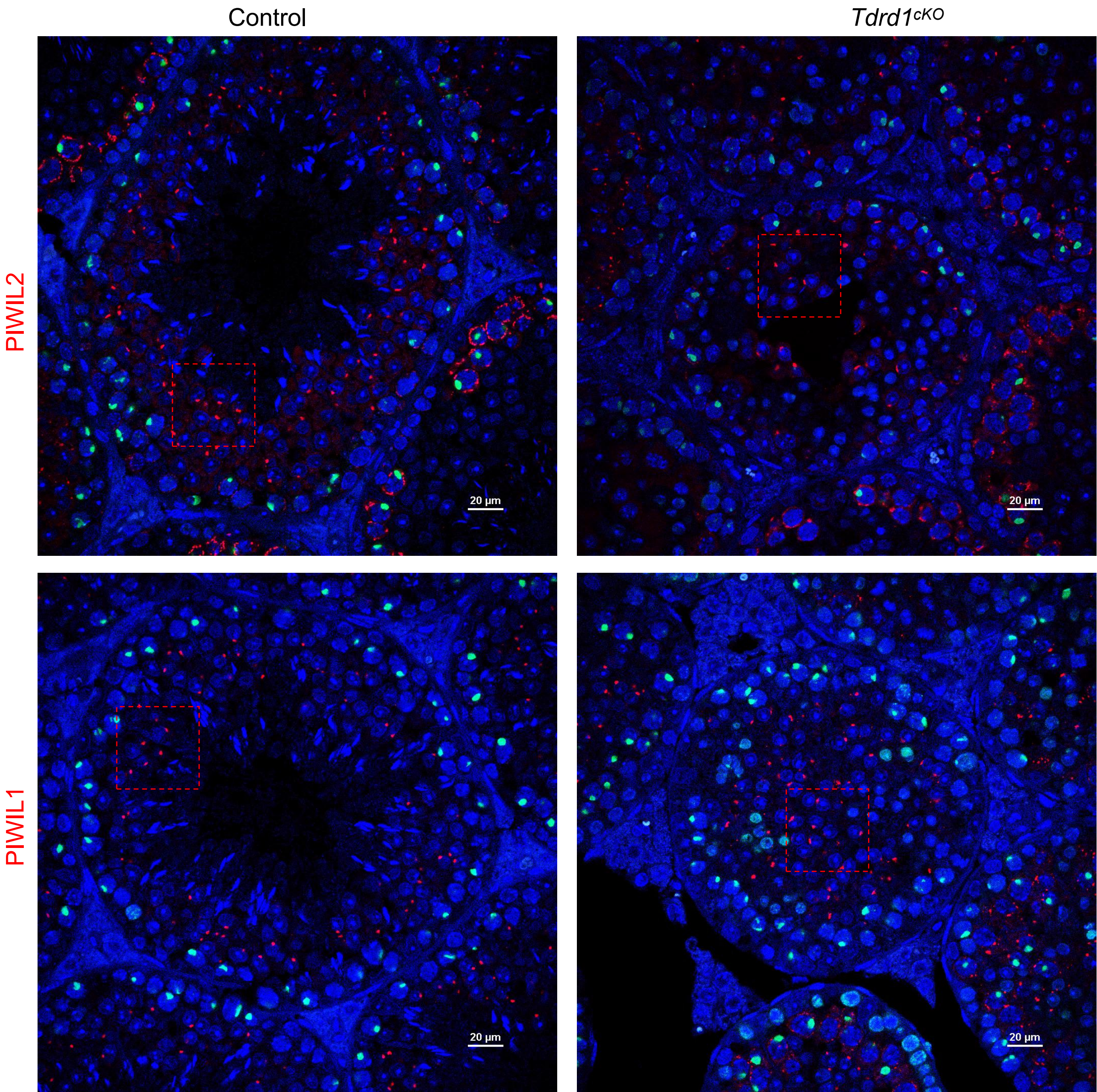

Supplement: Supplementary file 11 — Source data Fig. 6 [file 44318_2025_579_MOESM11_ESM.zip › Figure 6/6G/Figure 6G.pdf]

RS

Control

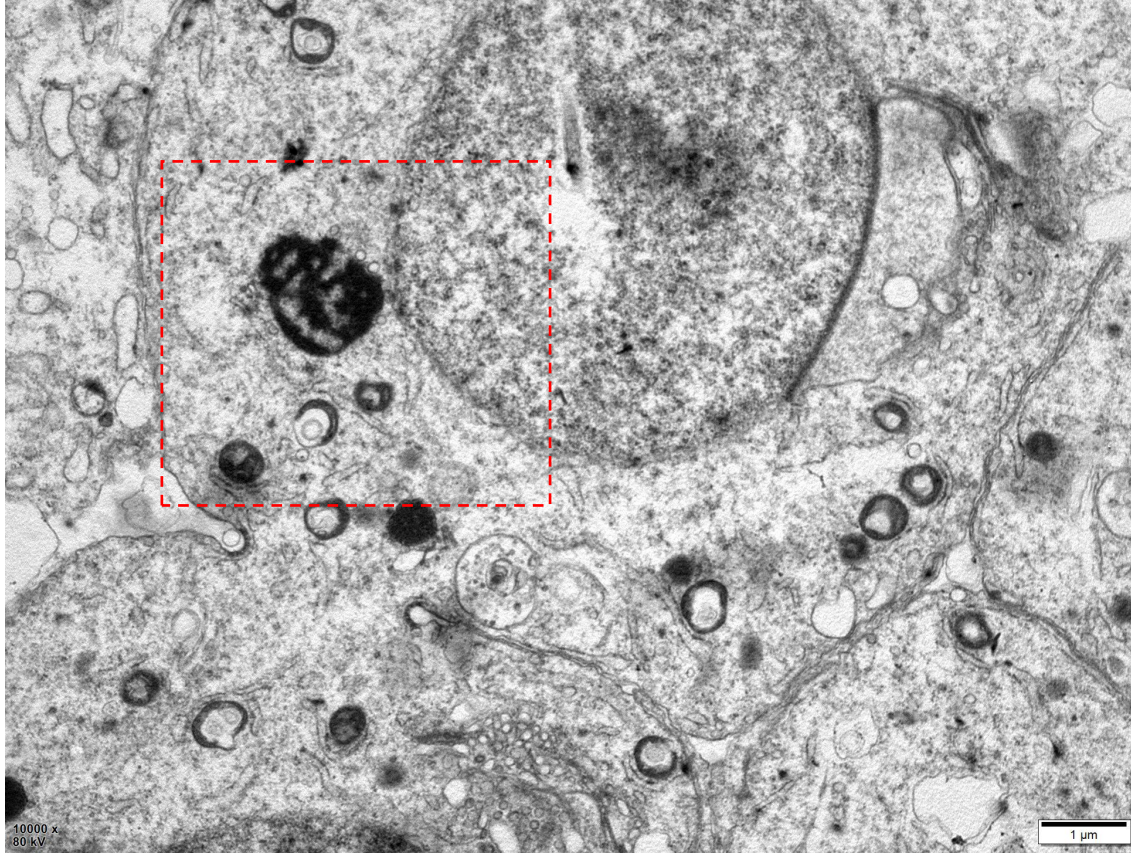

*Tdrd1*<sup>cko</sup>

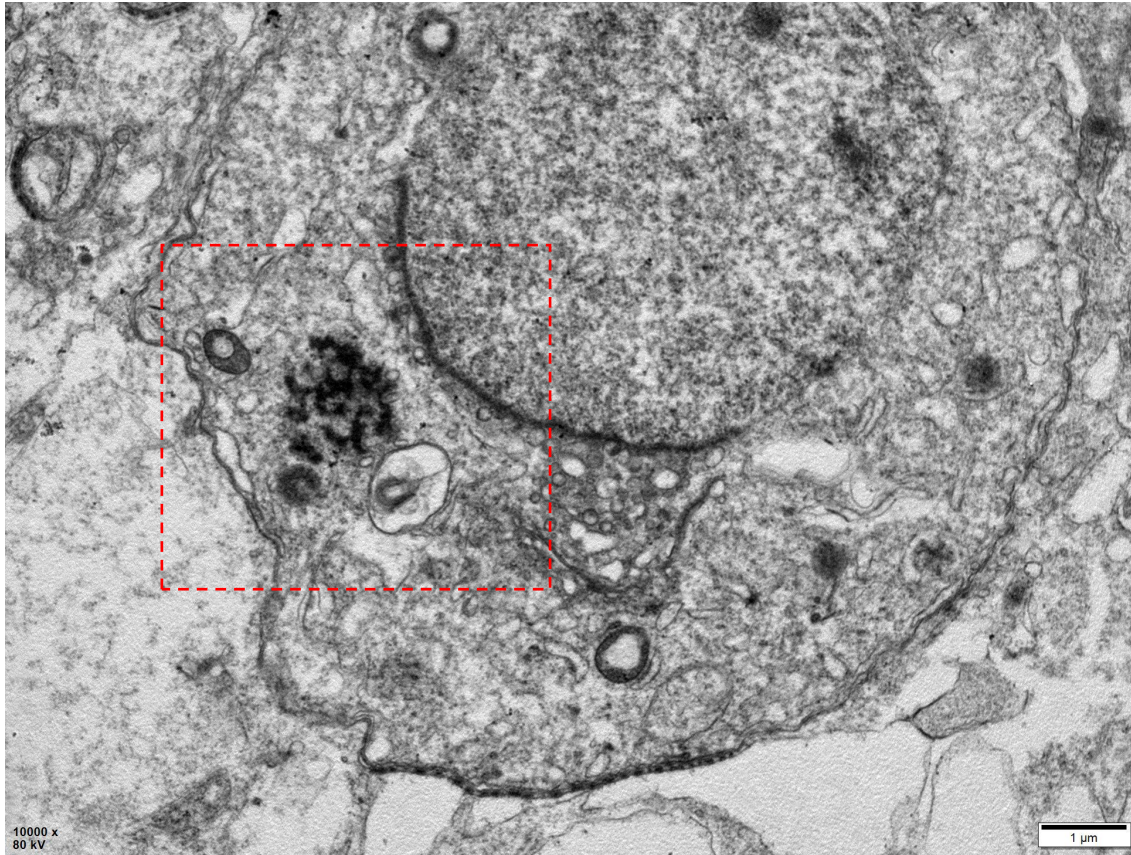

Supplement: Supplementary file 11 — Source data Fig. 6 [file 44318_2025_579_MOESM11_ESM.zip › Figure 6/6H/Figure 6H.pdf]

Figure 6J piRNA

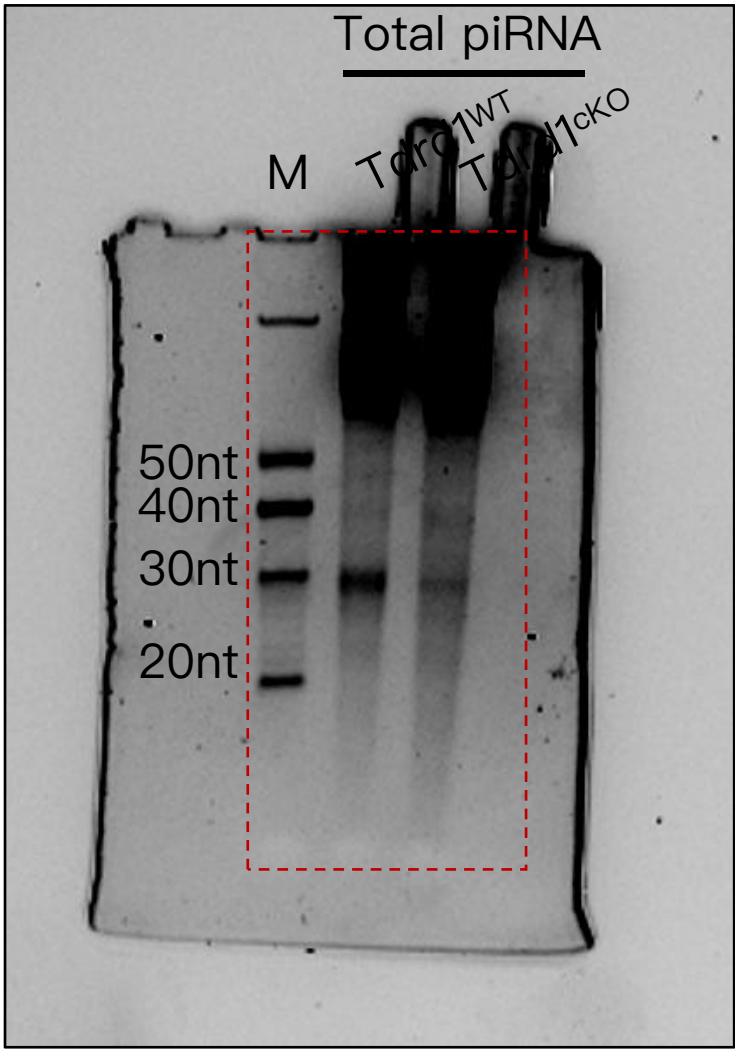

Supplement: Supplementary file 11 — Source data Fig. 6 [file 44318_2025_579_MOESM11_ESM.zip › Figure 6/6J/Figure 6J.pdf]

WT

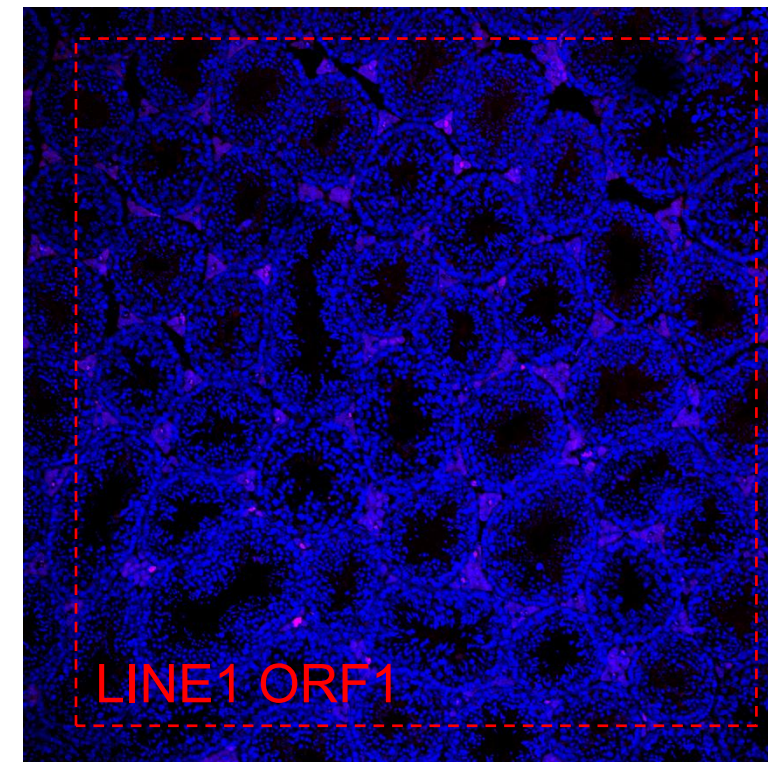

*Tdrd1*<sup>CKO</sup>

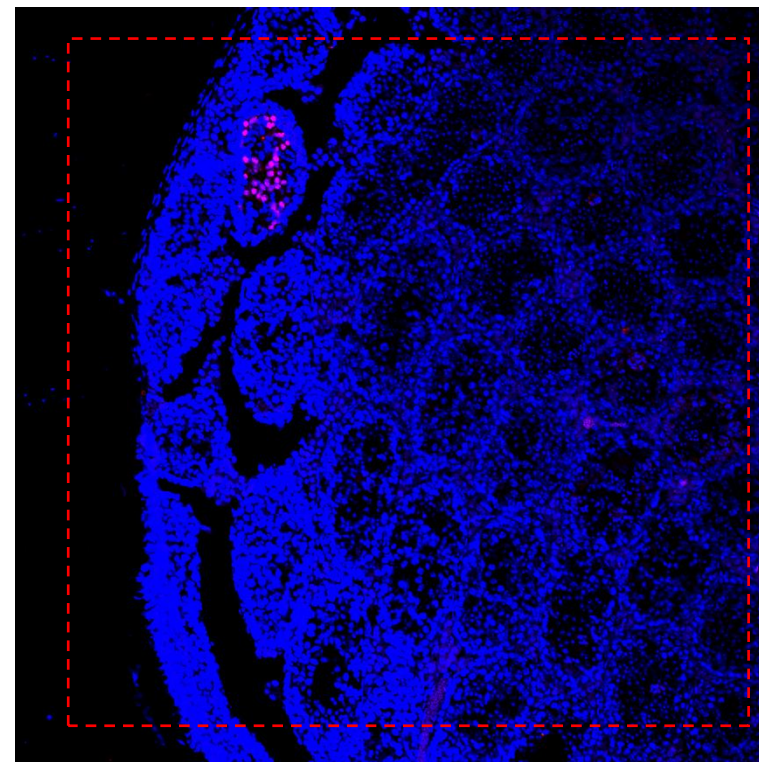

*Tdrd1*<sup>-/-</sup>

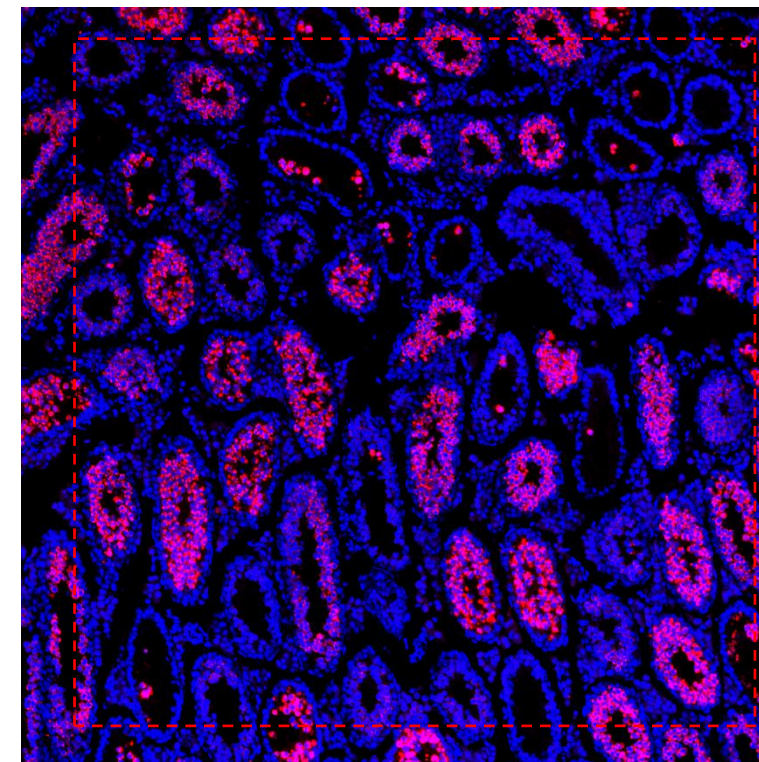

Supplement: Supplementary file 11 — Source data Fig. 6 [file 44318_2025_579_MOESM11_ESM.zip › Figure 6/6L/Figure 6L.pdf]

Pachytene Spermatocytes

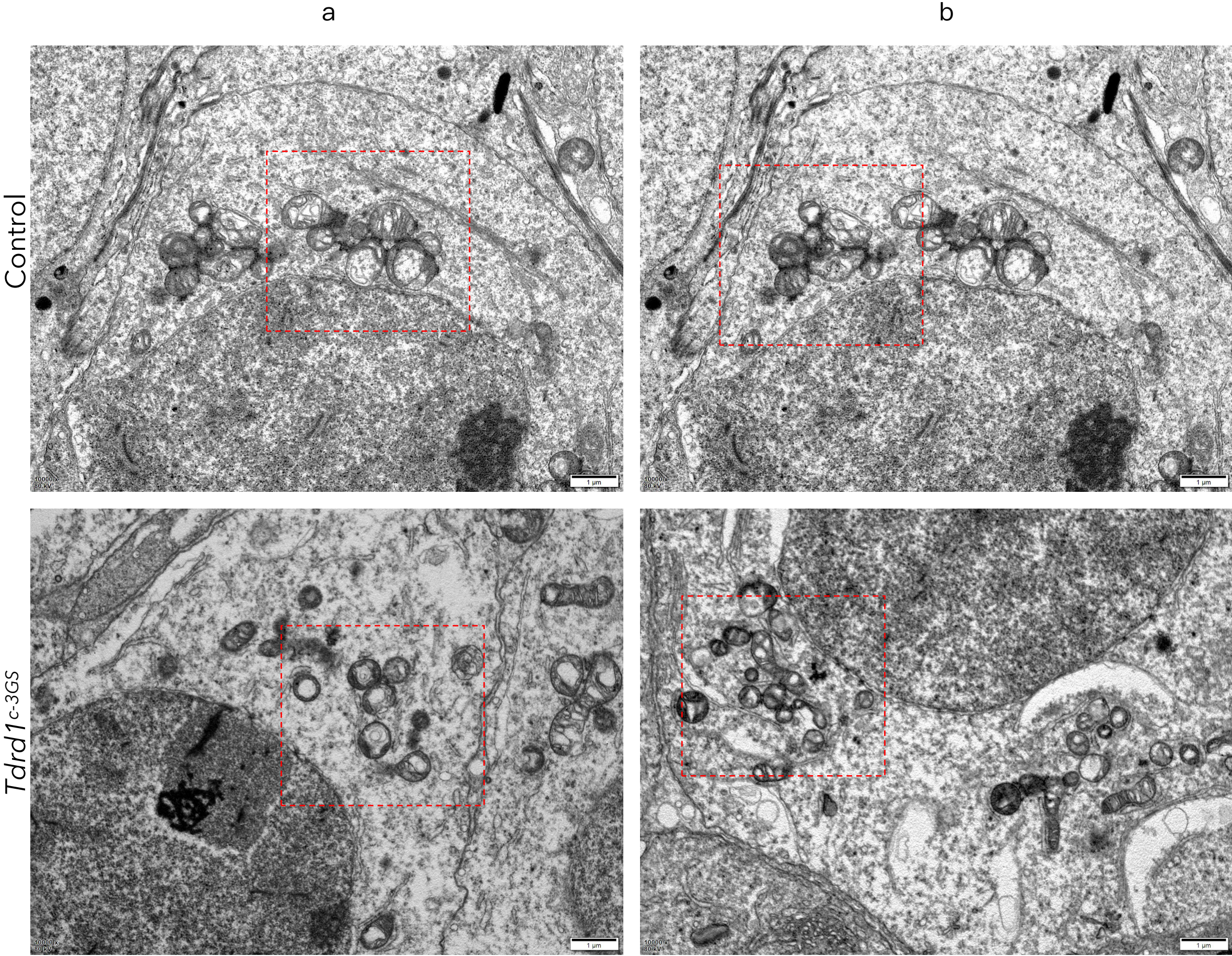

Supplement: Supplementary file 12 — Source data Fig. 7 [file 44318_2025_579_MOESM12_ESM.zip › Figure 7/7J/Figure 7J.pdf]

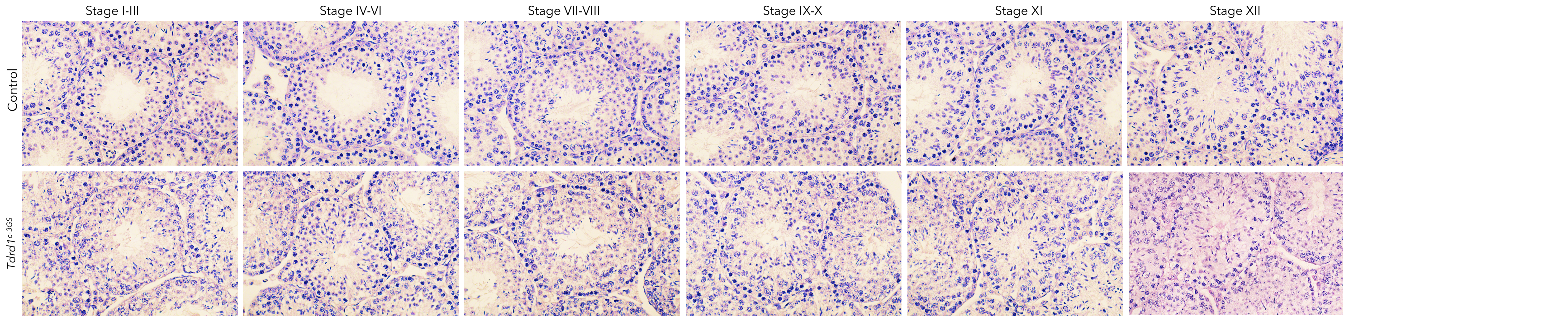

Supplement: Supplementary file 12 — Source data Fig. 7 [file 44318_2025_579_MOESM12_ESM.zip › Figure 7/7D/Figure 7D.pdf]

Figure 7B anti-TDRD1

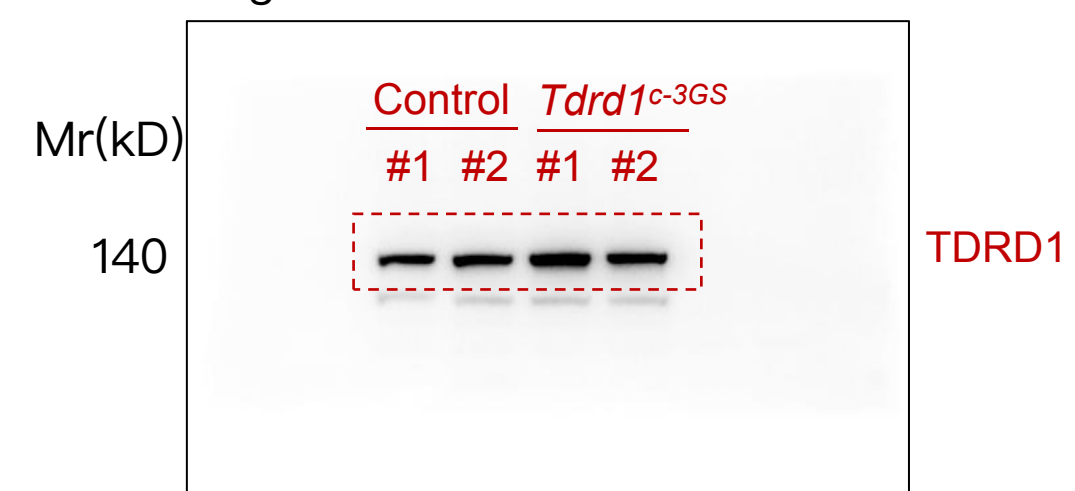

Figure 7B anti- $\beta$ -actin

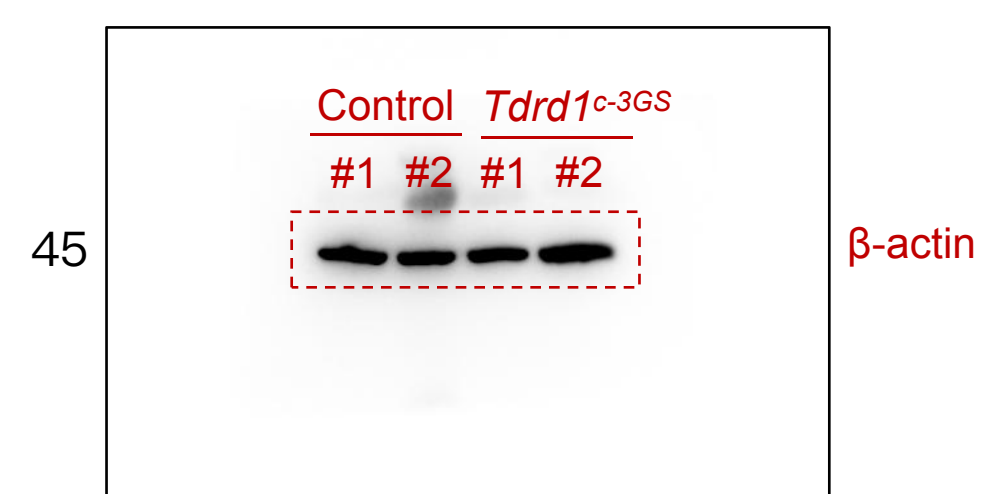

Supplement: Supplementary file 12 — Source data Fig. 7 [file 44318_2025_579_MOESM12_ESM.zip › Figure 7/7B/Figure 7B.pdf]

Control

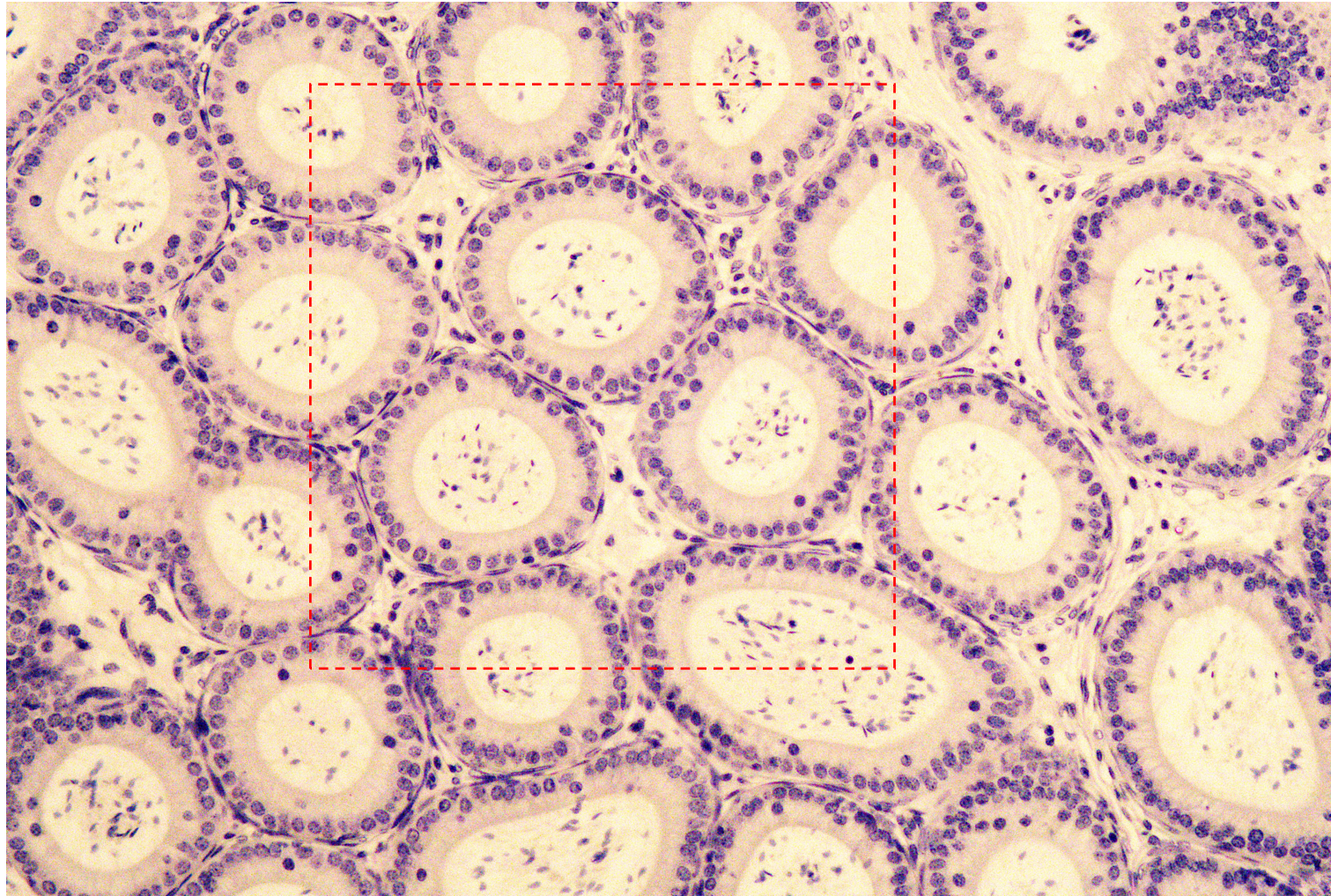

*Tdrd1*<sup>C-3GS</sup>

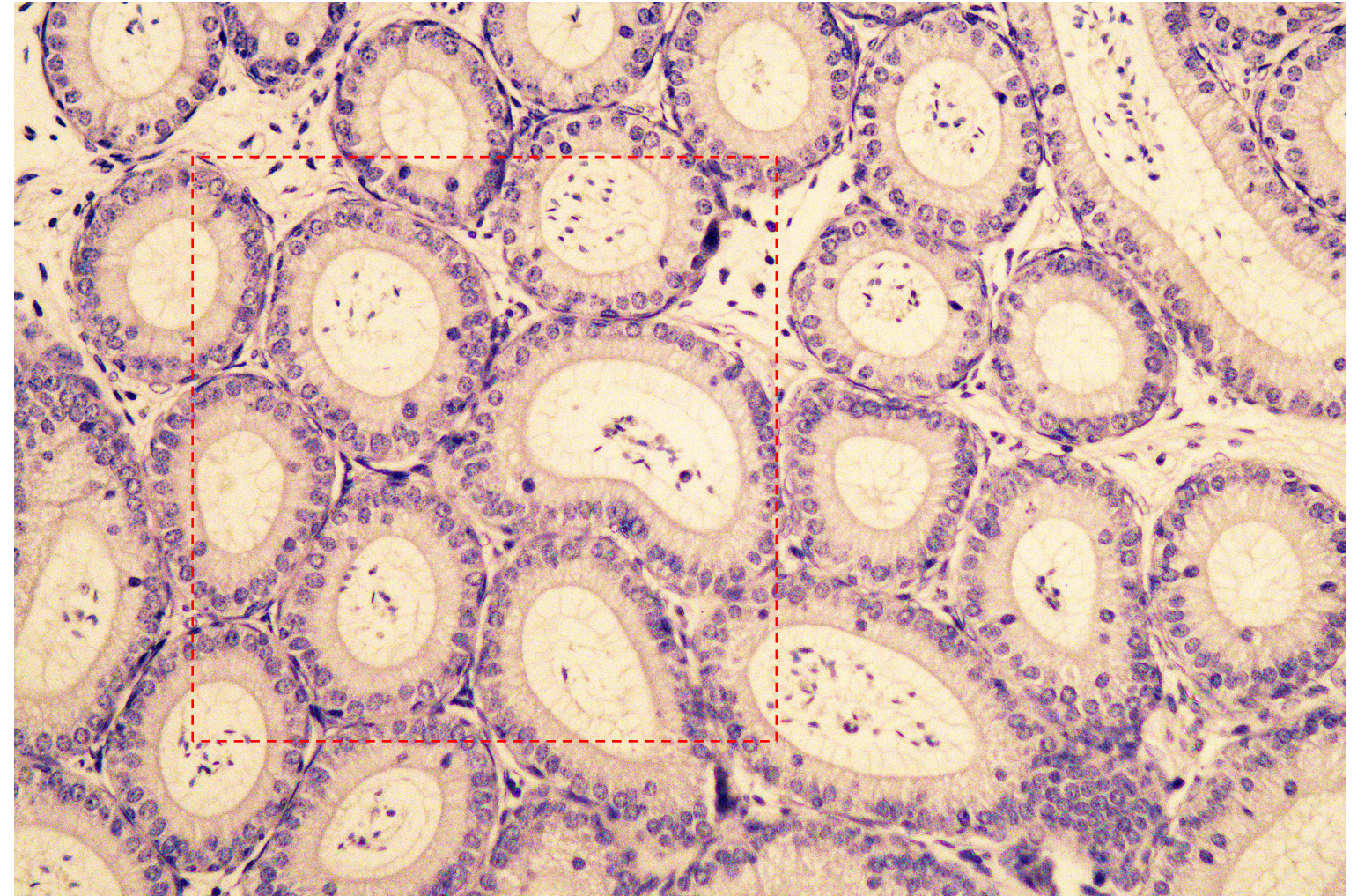

Caput  
Epididymis

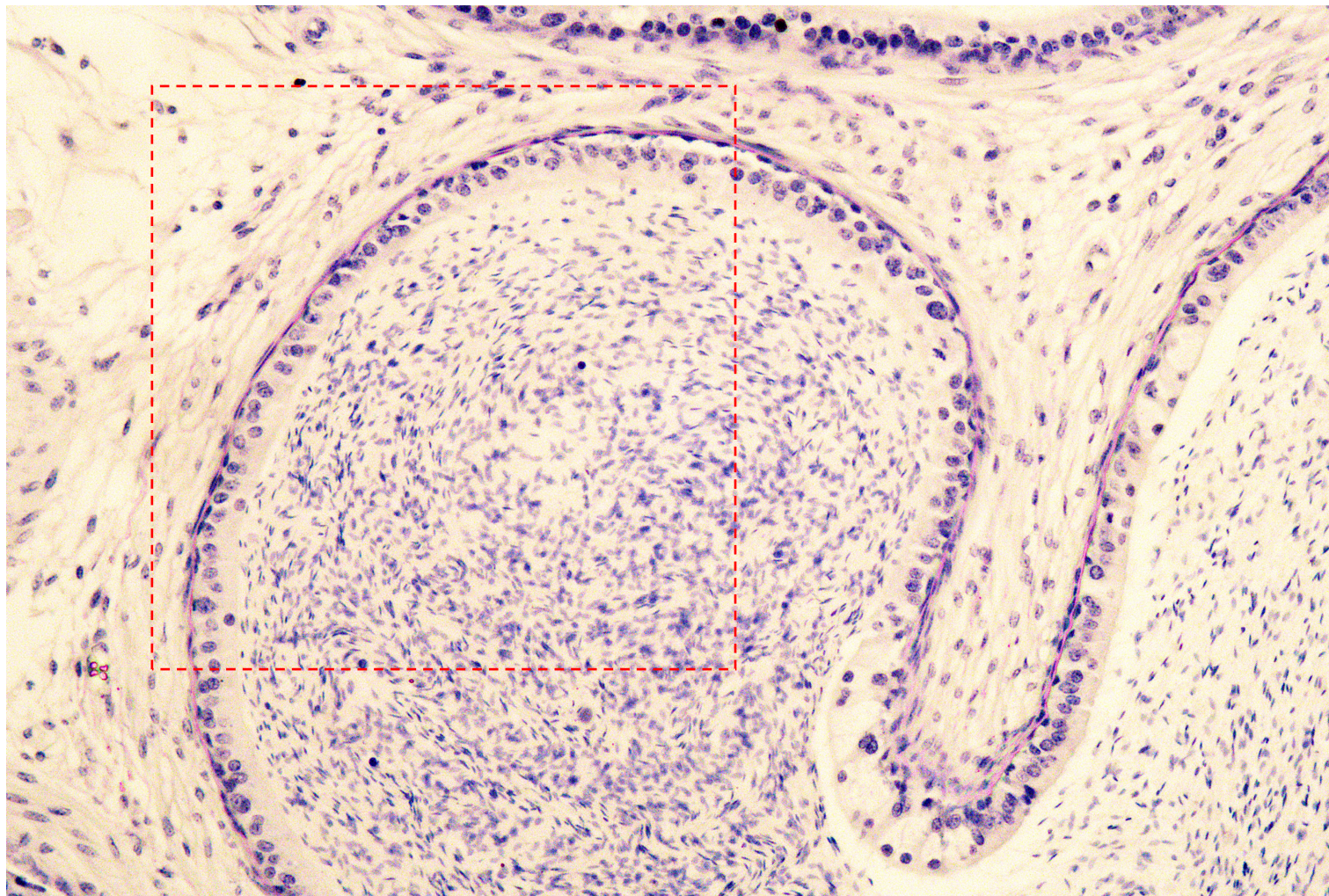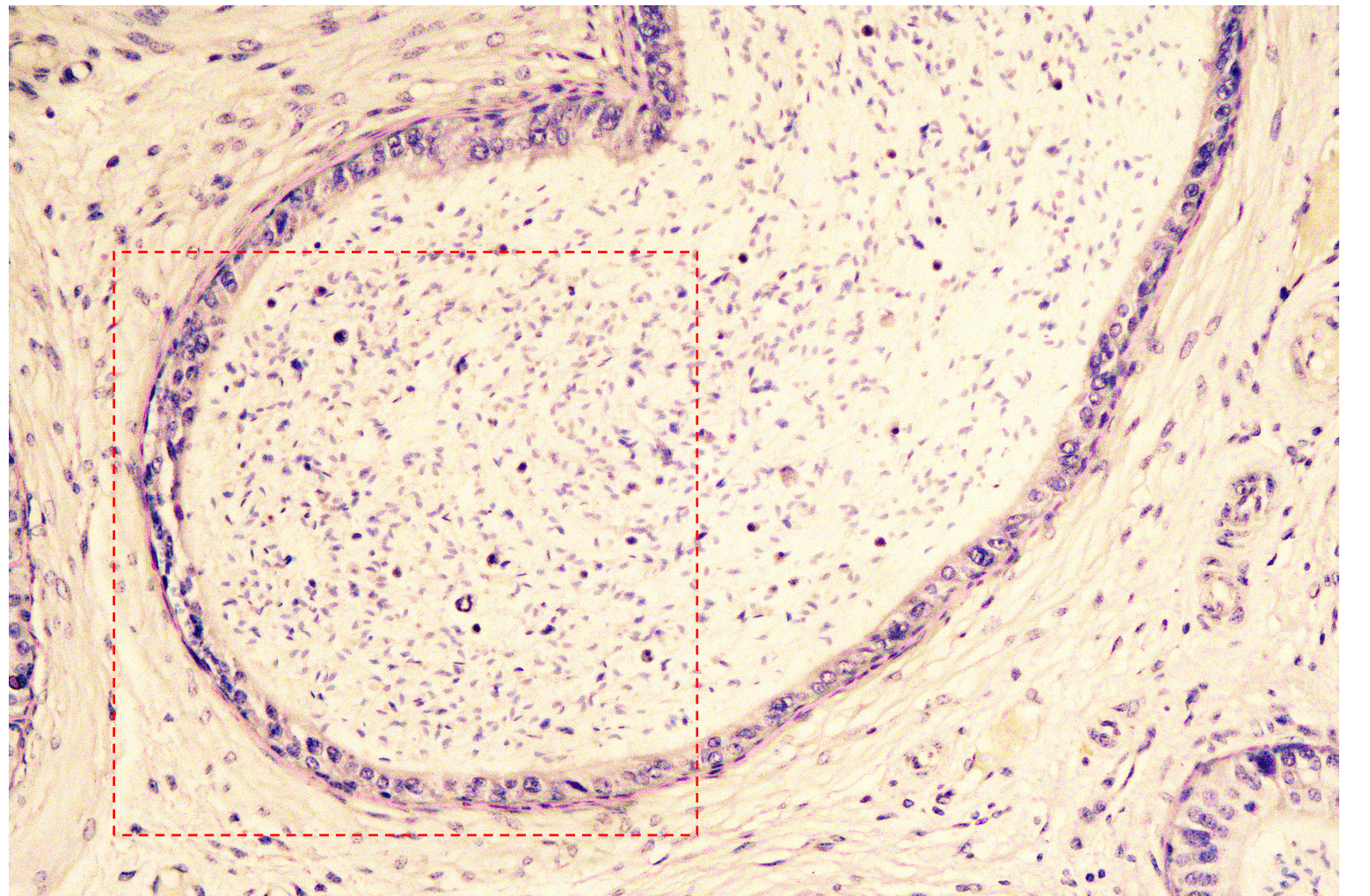

Cauda  
Epididymis

Supplement: Supplementary file 12 — Source data Fig. 7 [file 44318_2025_579_MOESM12_ESM.zip › Figure 7/7E/Figure 7E.pdf]

Round Spermatid

Control

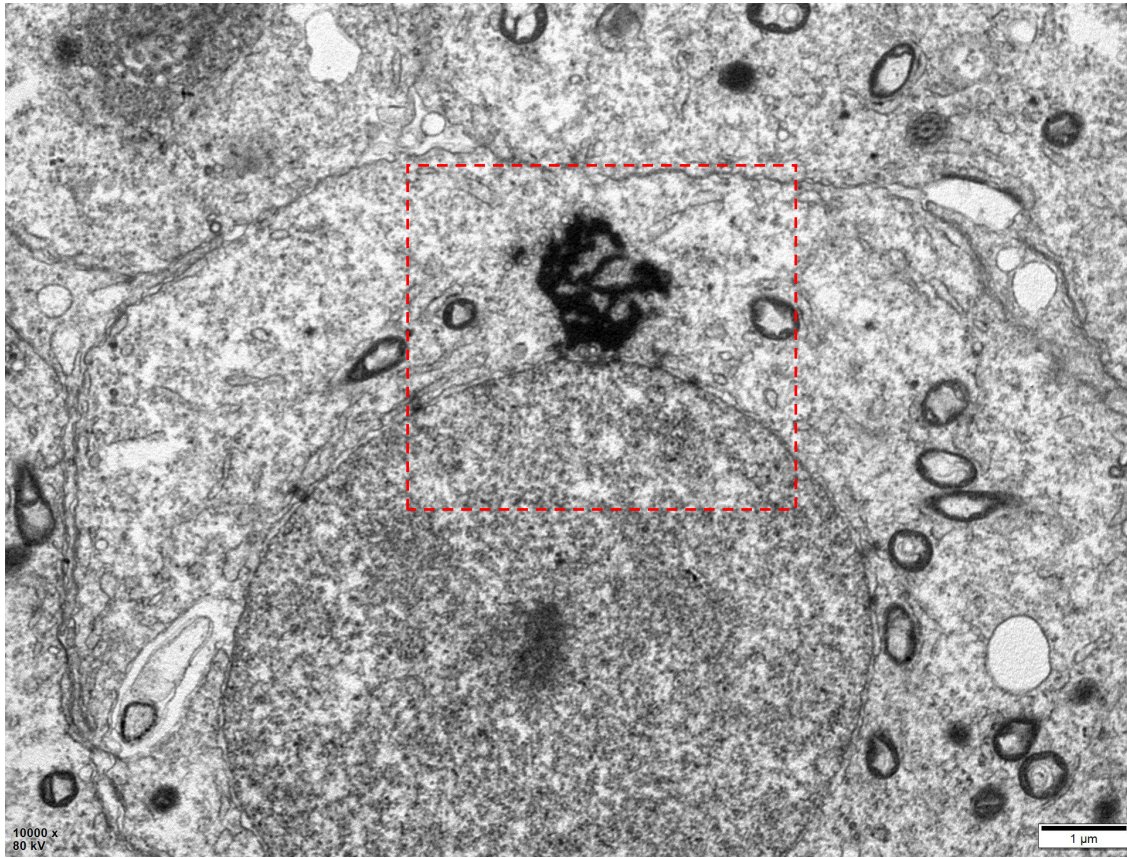

*Tdrd1<sup>c-3G5</sup>*

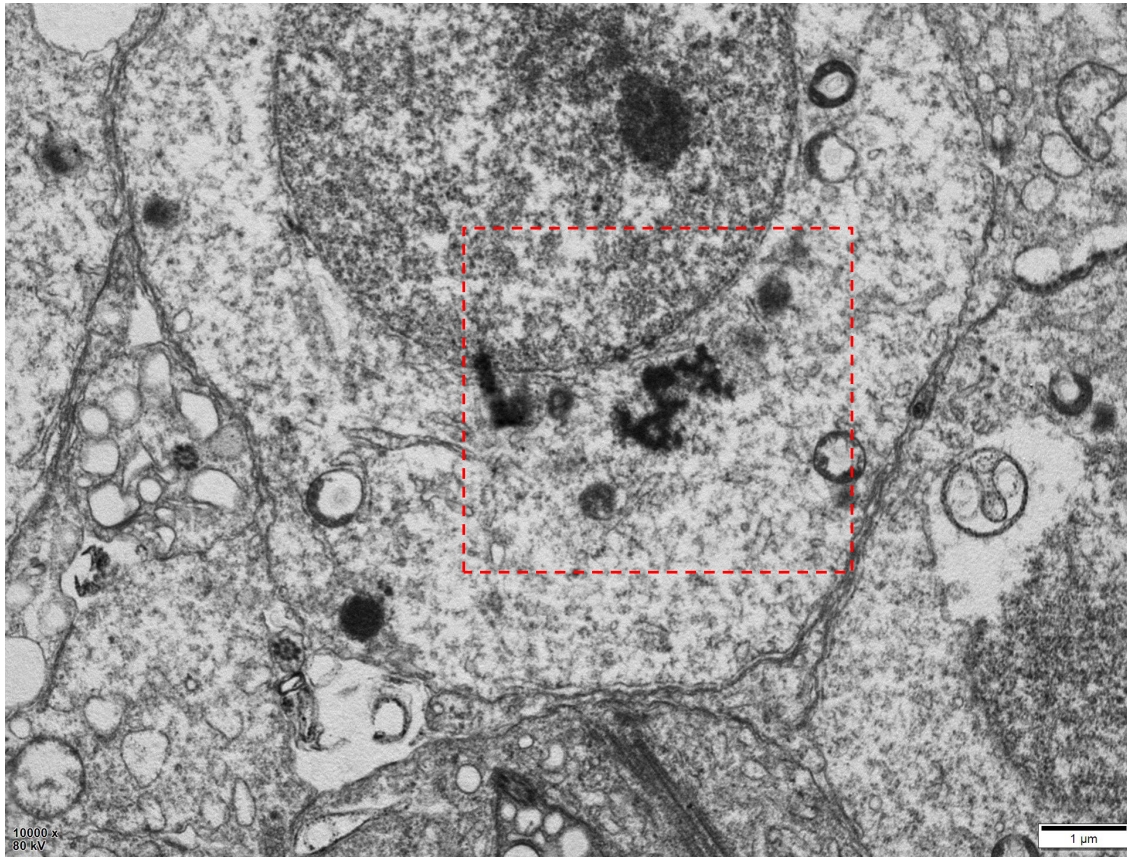

Supplement: Supplementary file 12 — Source data Fig. 7 [file 44318_2025_579_MOESM12_ESM.zip › Figure 7/7K/Figure 7K.pdf]

Control

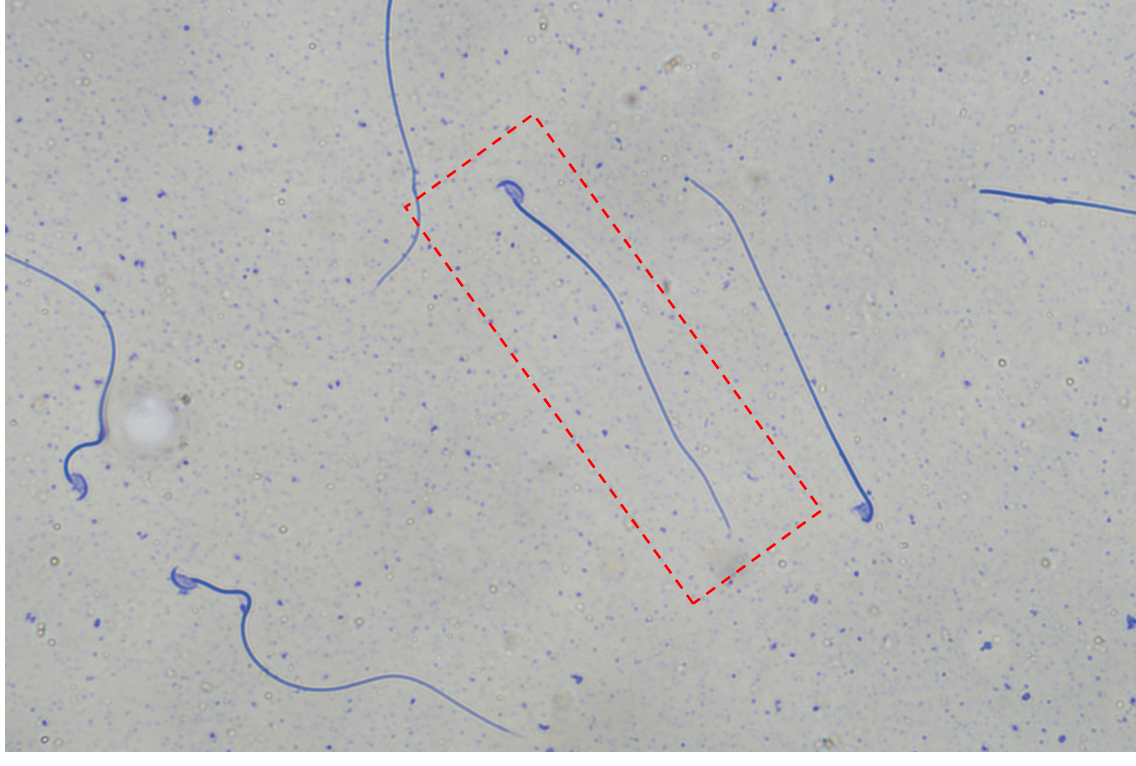

a

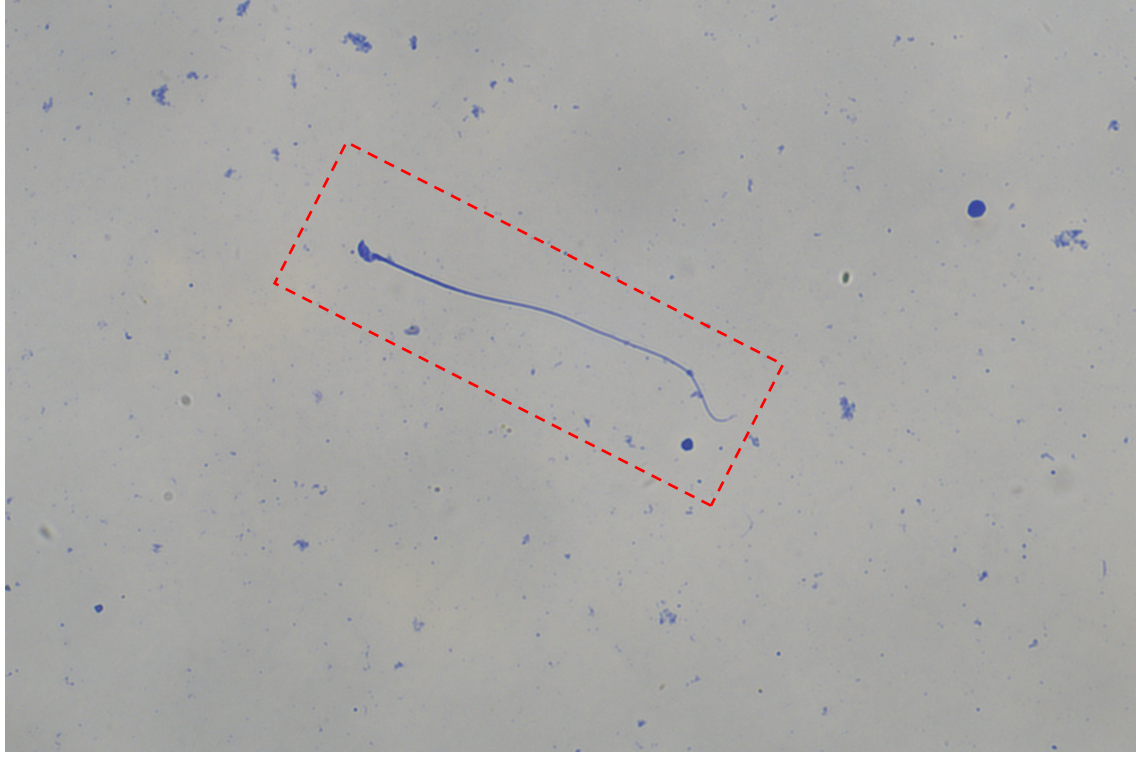

b

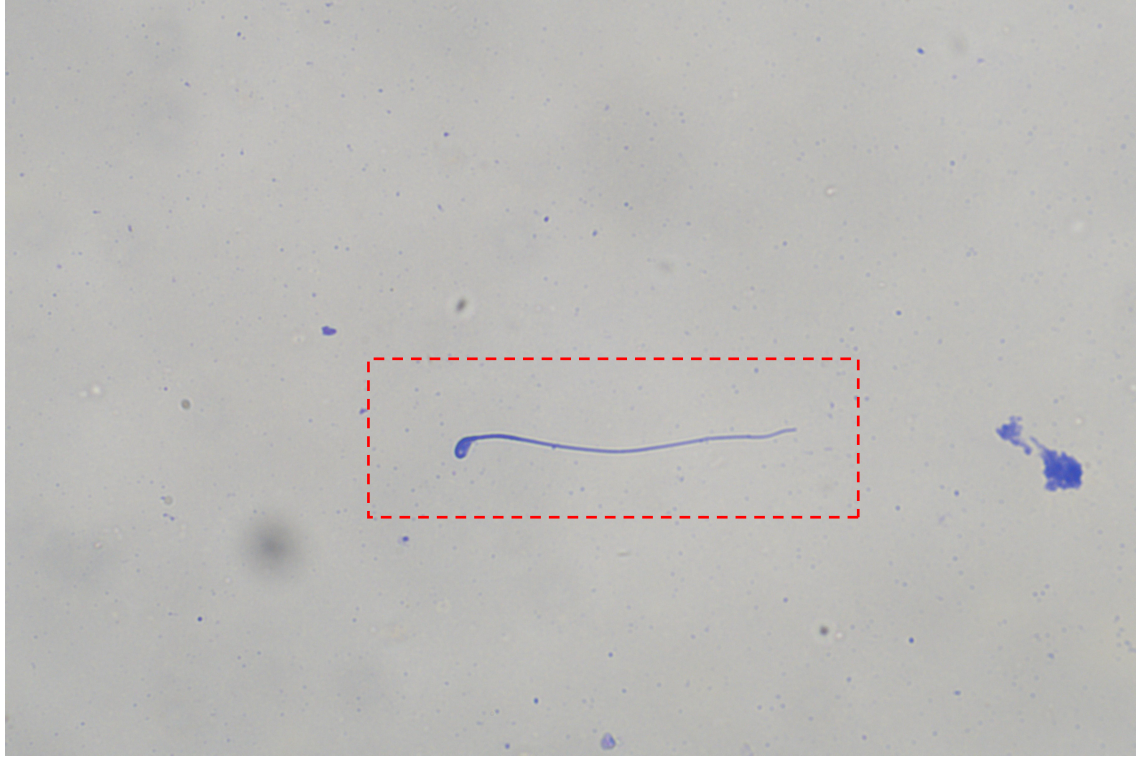

c

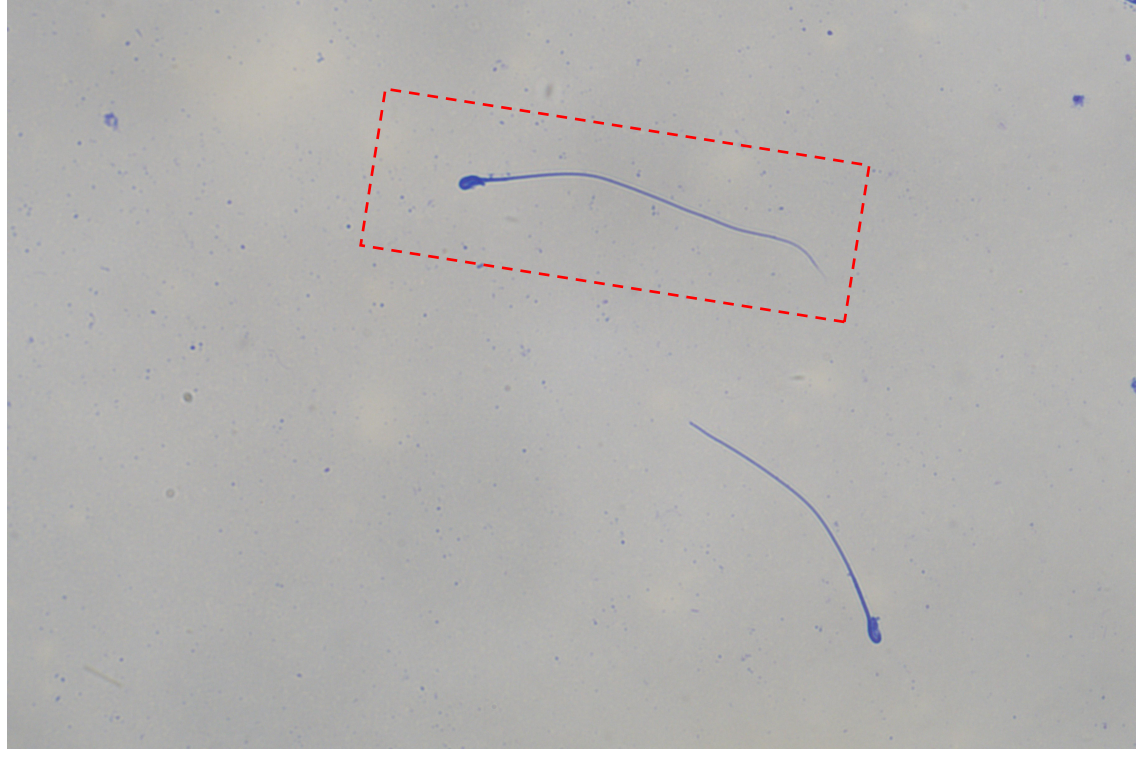

d

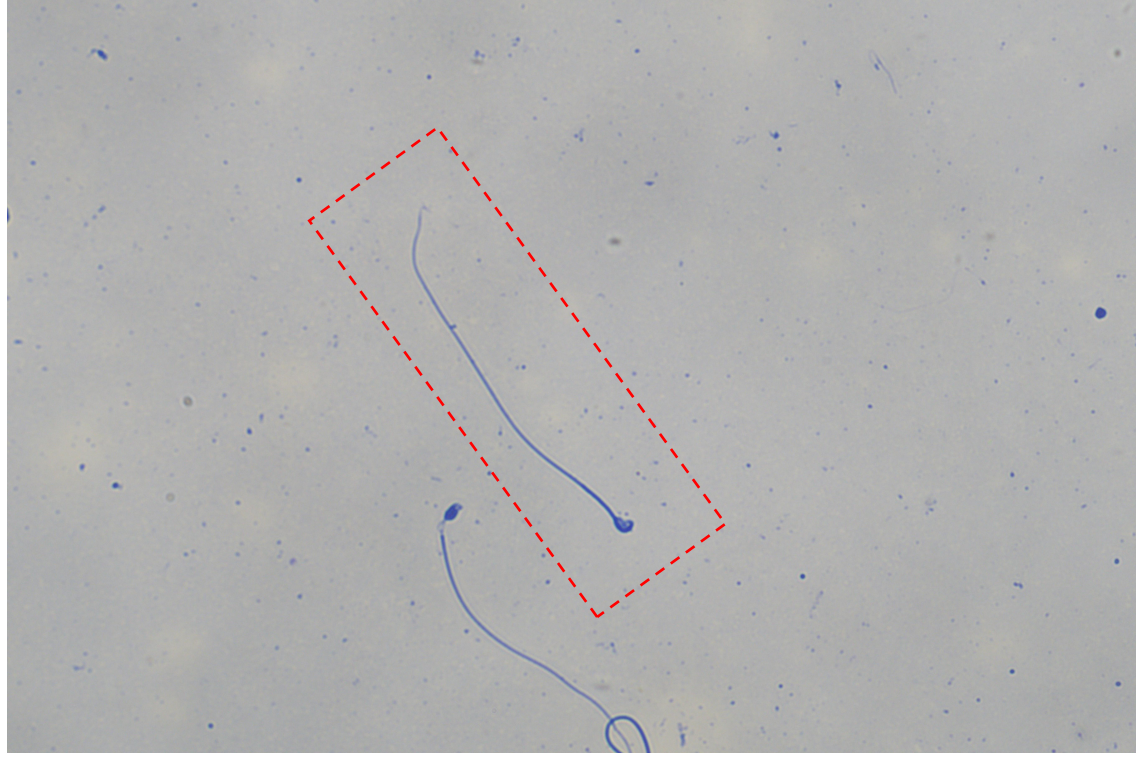

e

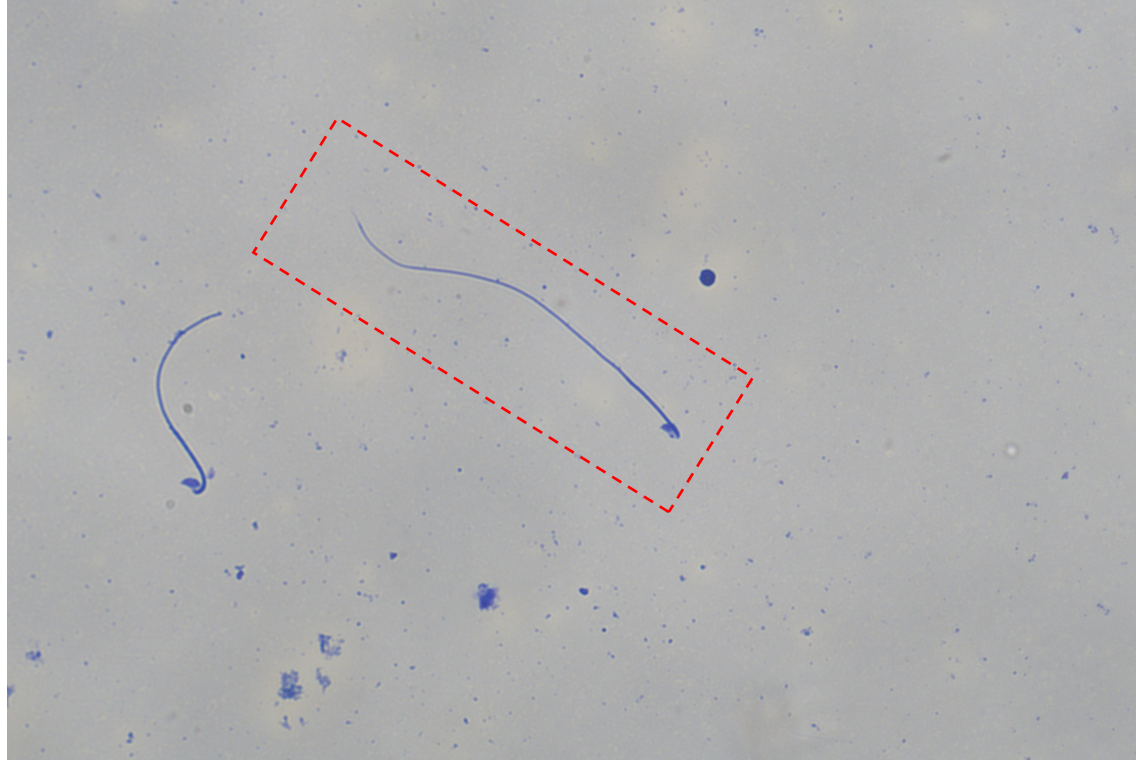

Supplement: Supplementary file 12 — Source data Fig. 7 [file 44318_2025_579_MOESM12_ESM.zip › Figure 7/7G/Figure 7G.pdf]
