# Supplementary material for: PIWI proteins tether the piRNA biogenesis machinery to mitochondria during mammalian spermatogenesis
Source: EMBO J. 2025 Sep 29;44(22):6397–424. doi: 10.1038/s44318-025-00579-x (PMC12624062; doi:10.1038/s44318-025-00579-x)

Figure 2G Input anti-PIWIL2

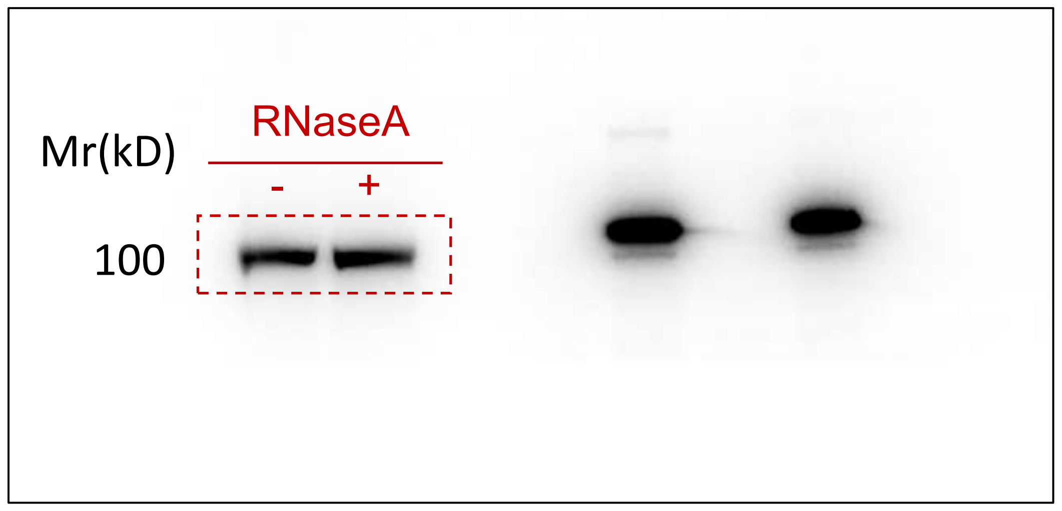

Figure 2G IP anti-PIWIL2

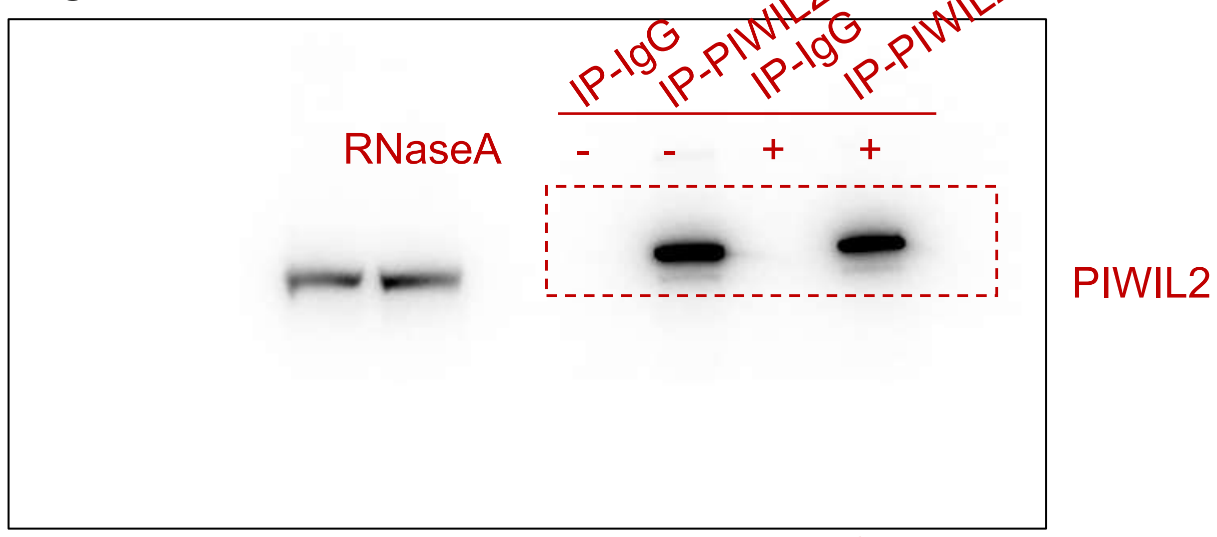

Figure 2G Input anti-ASZ1

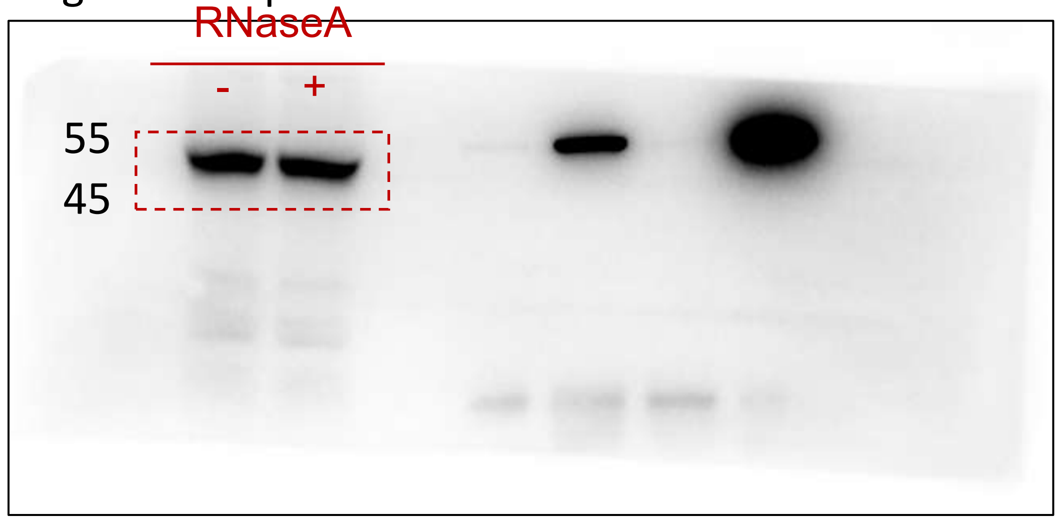

Figure 2G IP anti-ASZ1

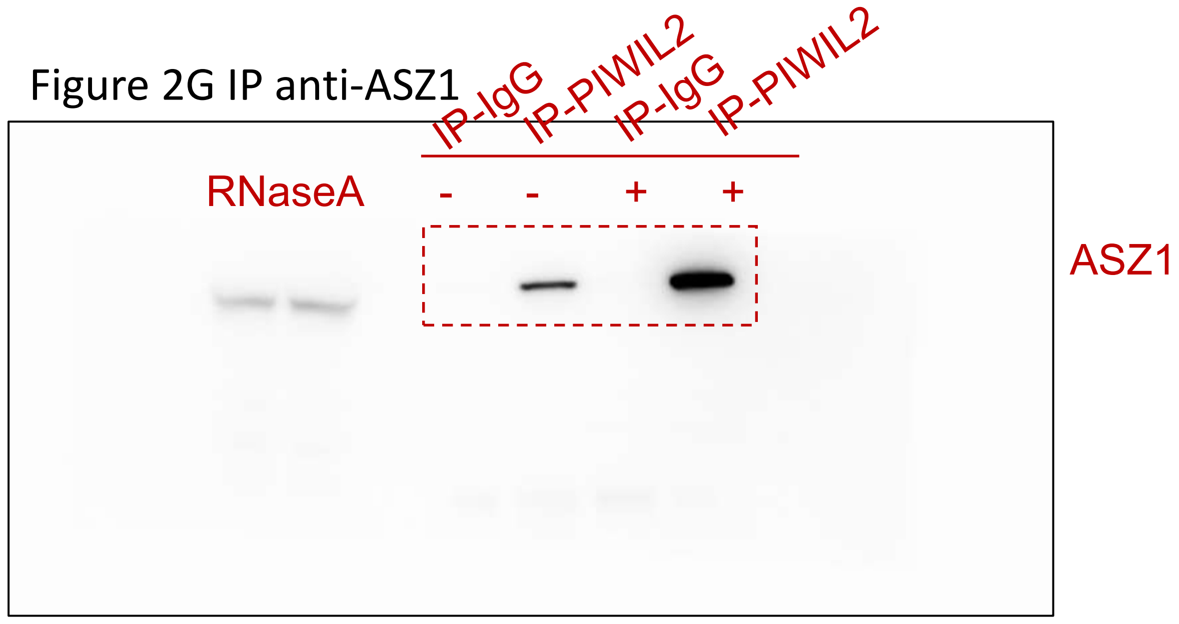

Figure 2G Input anti-TDRD1

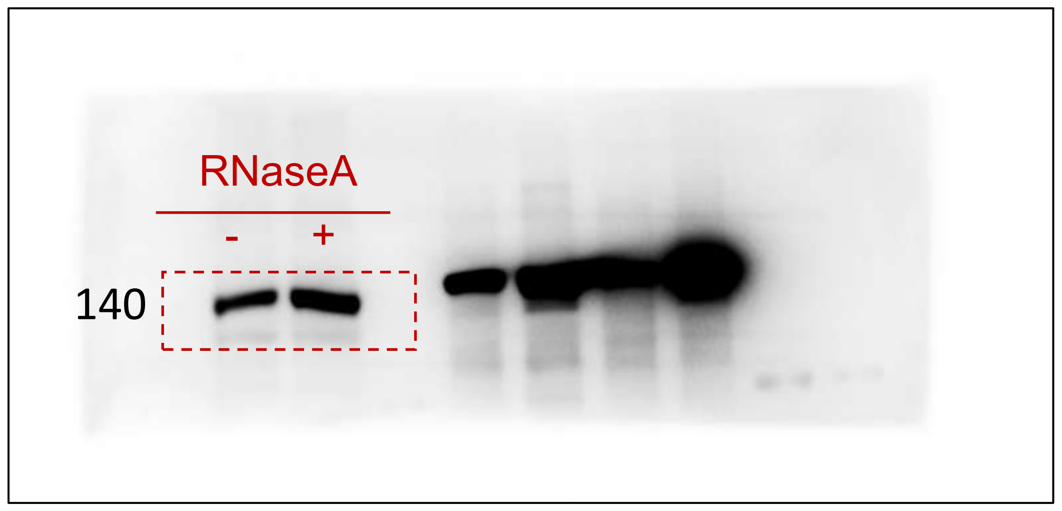

Figure 2G IP anti-TDRD1

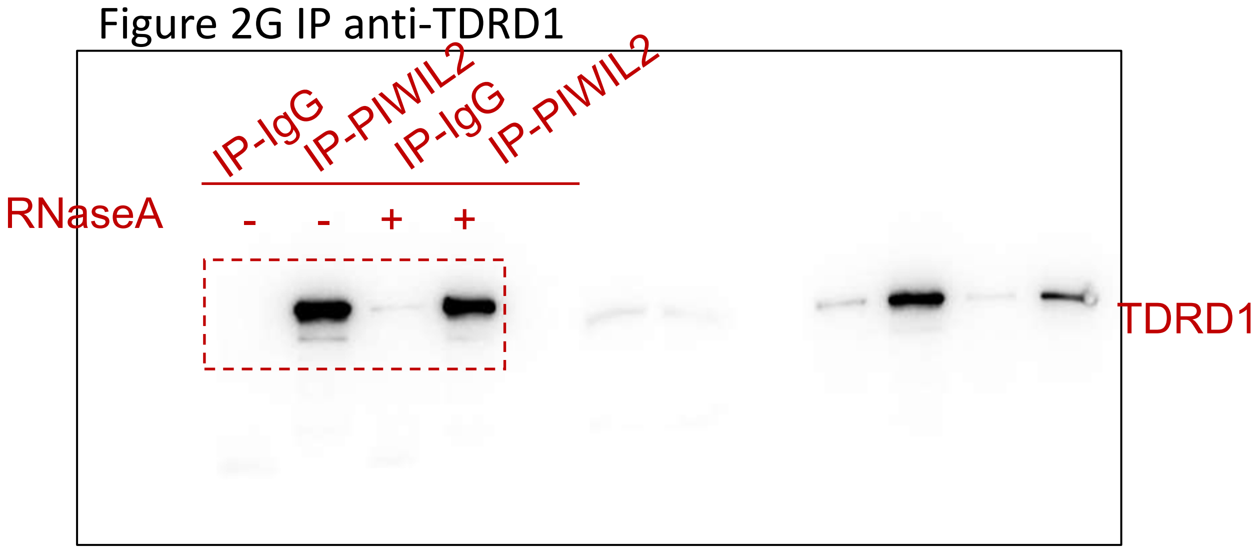

Figure 2G Input anti-β-actin

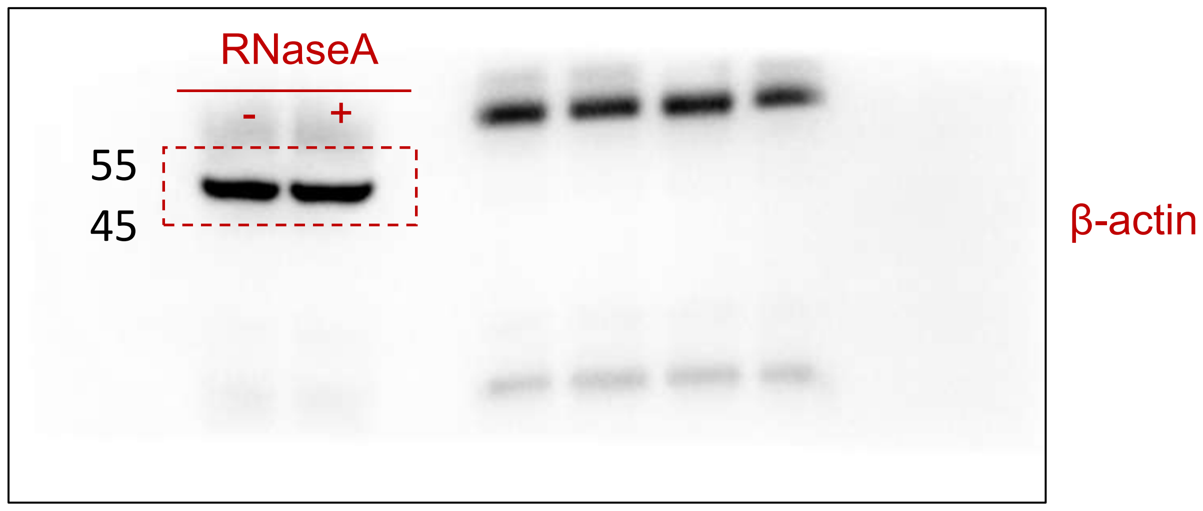

Supplement: Supplementary file 7 — Source data Fig. 2 [file 44318_2025_579_MOESM7_ESM.zip › Figure 2/2G/Figure 2G_20250707170637.pdf]
