# Supplementary material for: PIWI proteins tether the piRNA biogenesis machinery to mitochondria during mammalian spermatogenesis
Source: EMBO J. 2025 Sep 29;44(22):6397–424. doi: 10.1038/s44318-025-00579-x (PMC12624062; doi:10.1038/s44318-025-00579-x)
Supplement: Supplementary file 7 — Source data Fig. 2 [file 44318_2025_579_MOESM7_ESM.zip › Figure 2/2I/Figure 2I.pdf]

Figure 2I Input anti–TDRD1

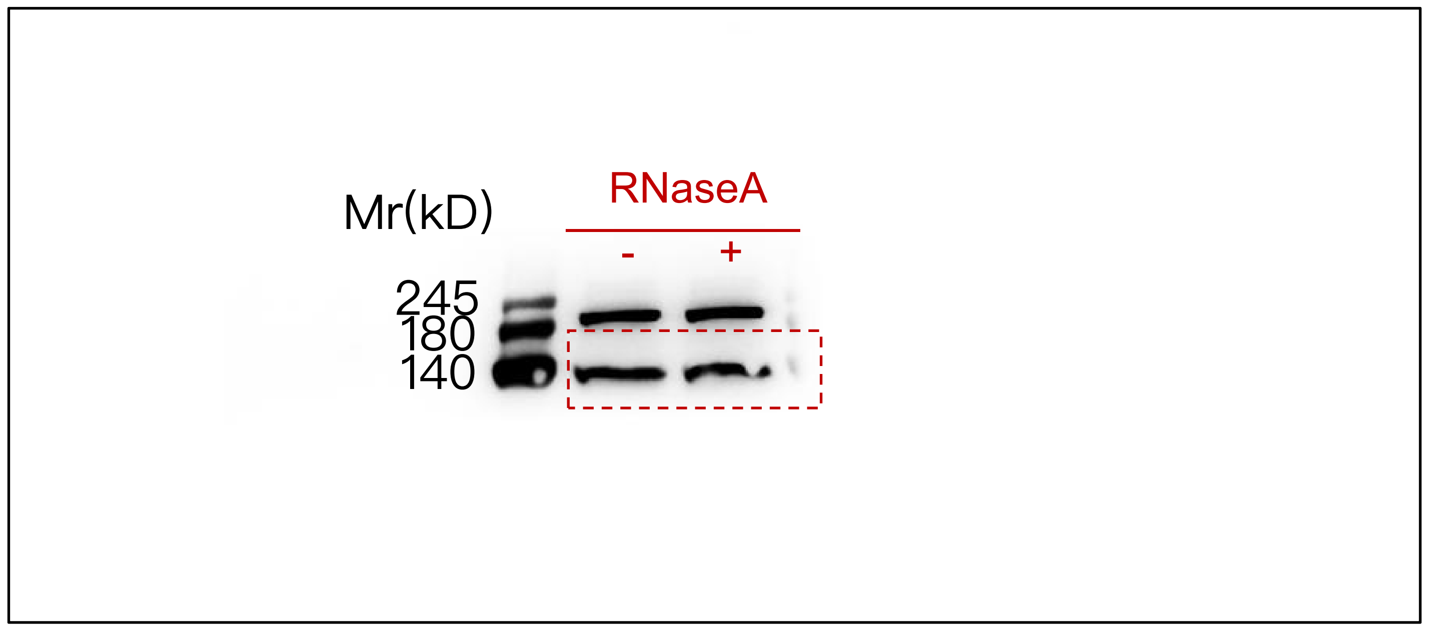

Figure 2I IP anti–TDRD1

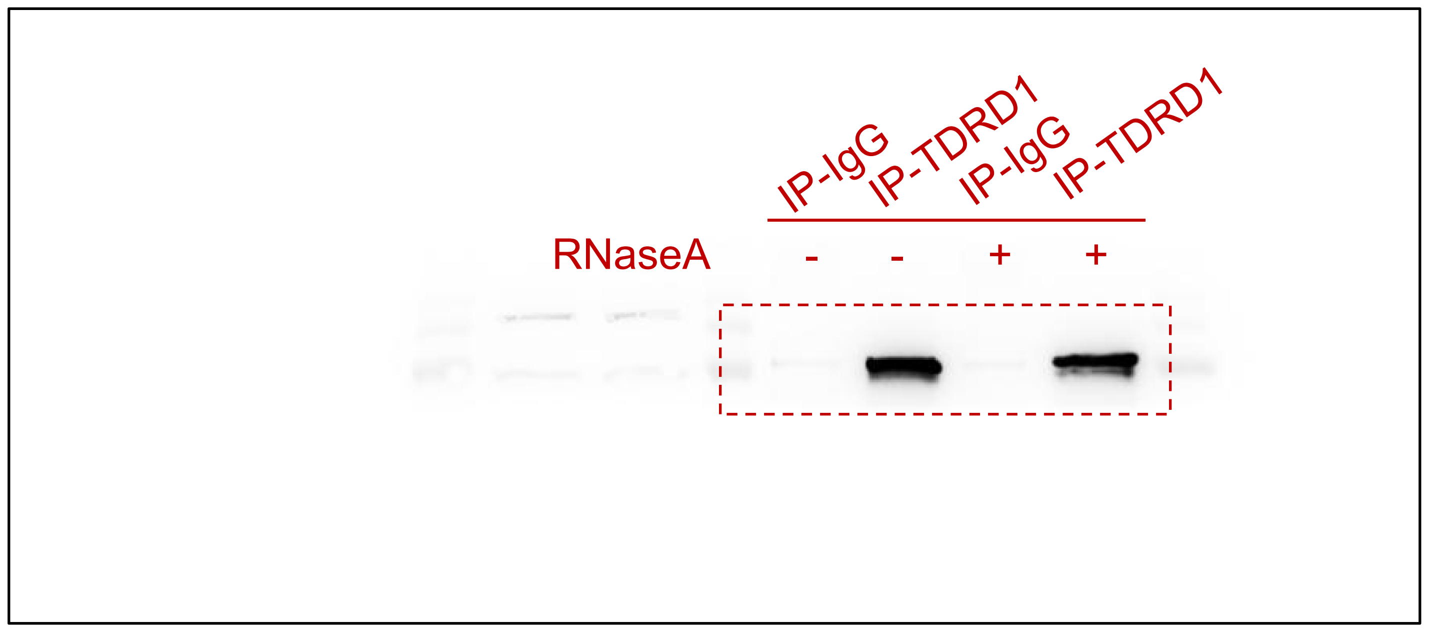

TDRD1

Figure 2I Input anti–PIWIL2

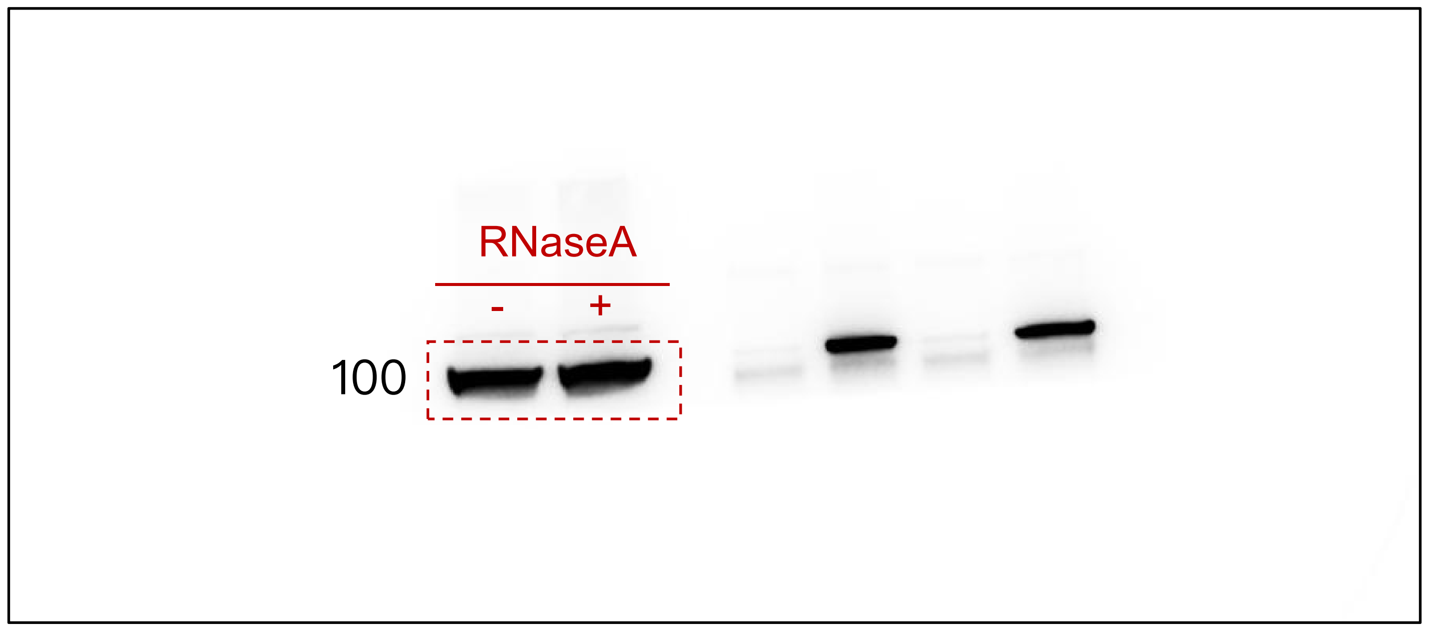

Figure 2I IP anti–PIWIL2

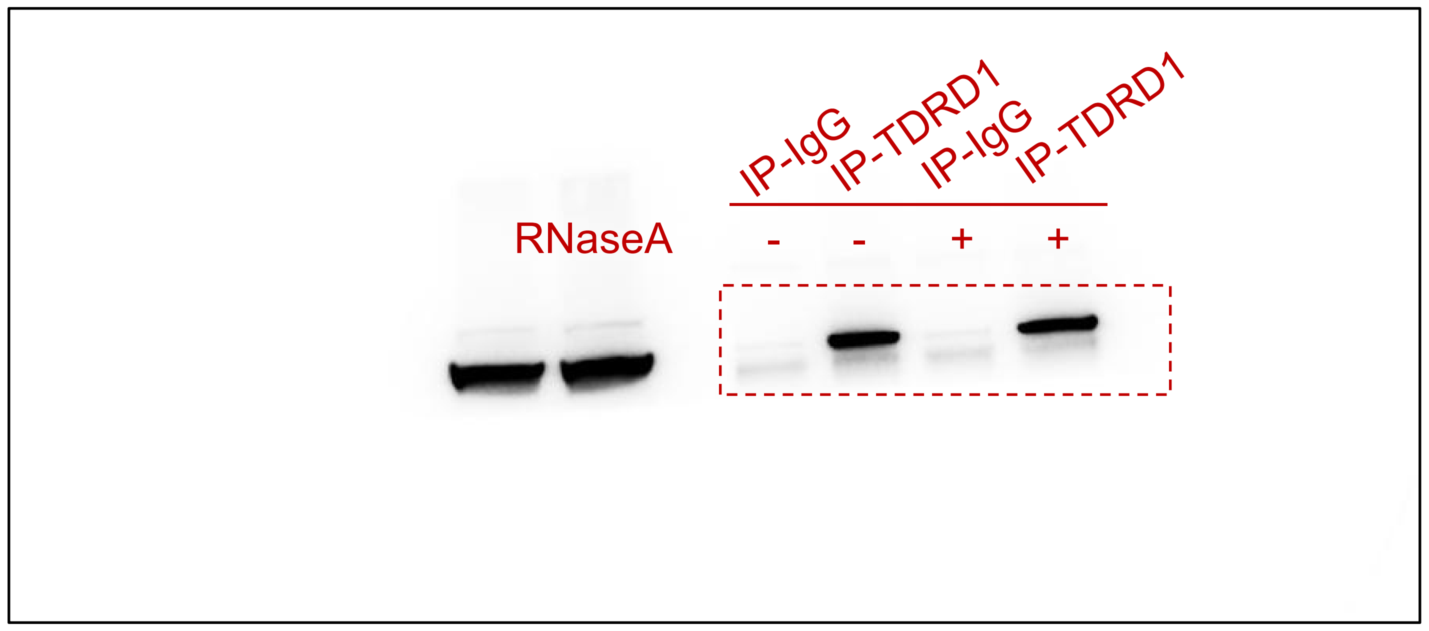

PIWIL2

Figure 2I Input anti–ASZ1

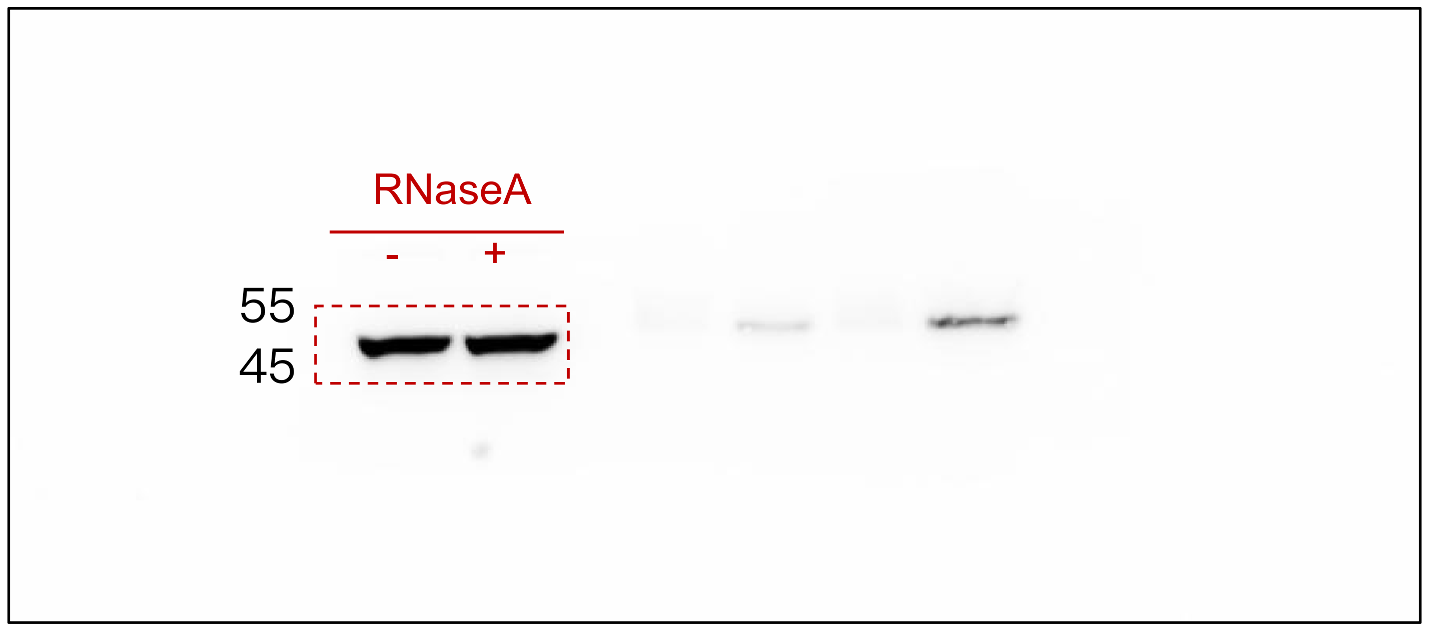

Figure 2I IP anti–ASZ1

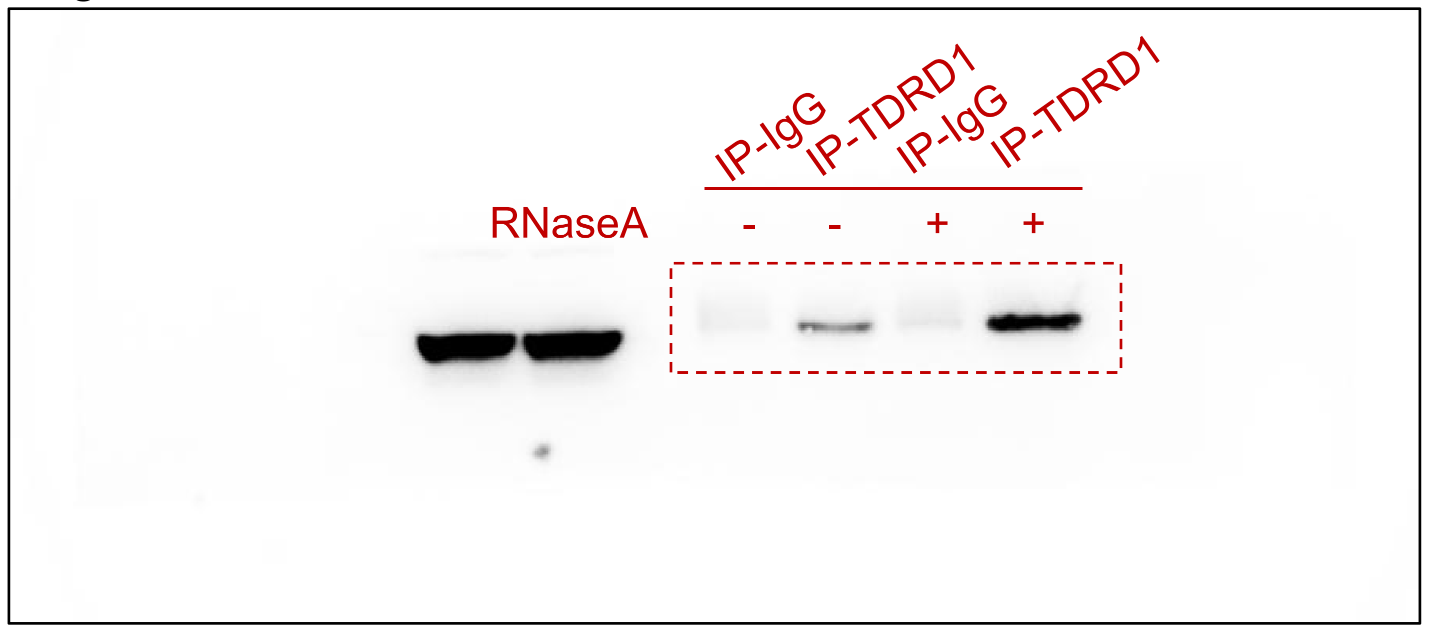

ASZ1

Figure 2I Input anti–β-actin

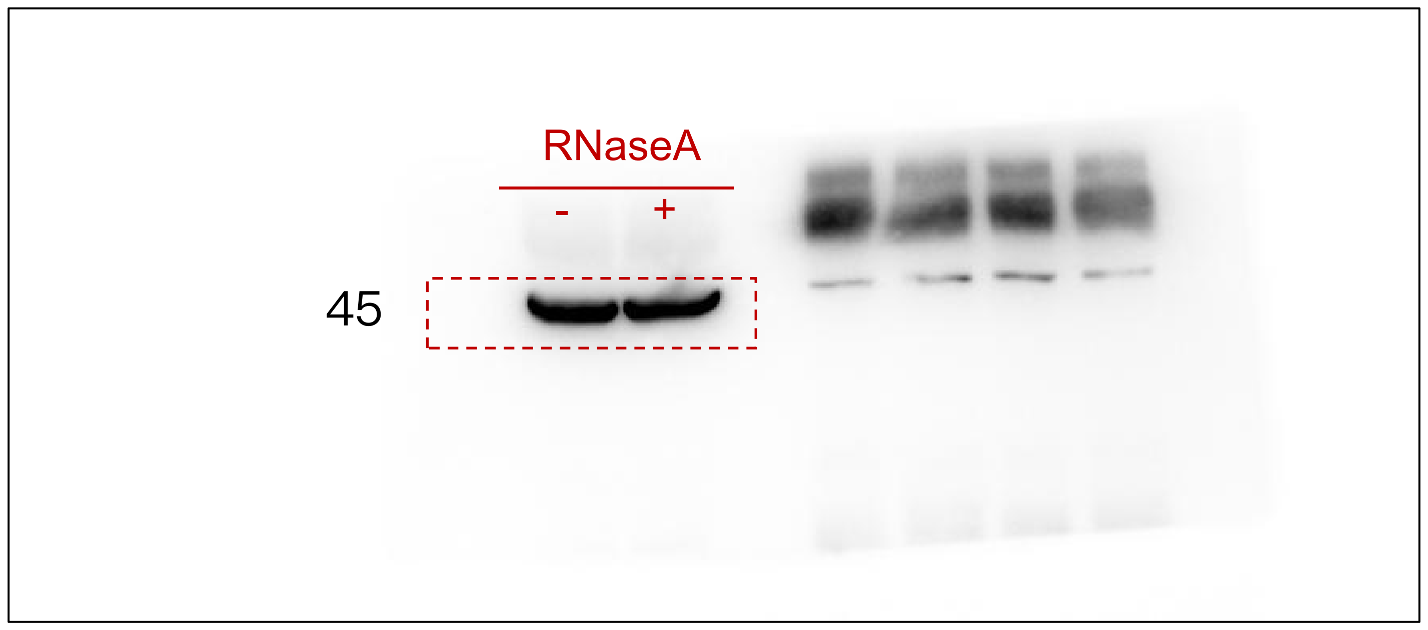

β-actin
