# Supplementary material for: PIWI proteins tether the piRNA biogenesis machinery to mitochondria during mammalian spermatogenesis
Source: EMBO J. 2025 Sep 29;44(22):6397–424. doi: 10.1038/s44318-025-00579-x (PMC12624062; doi:10.1038/s44318-025-00579-x)

Figure 3C Input anti–HA

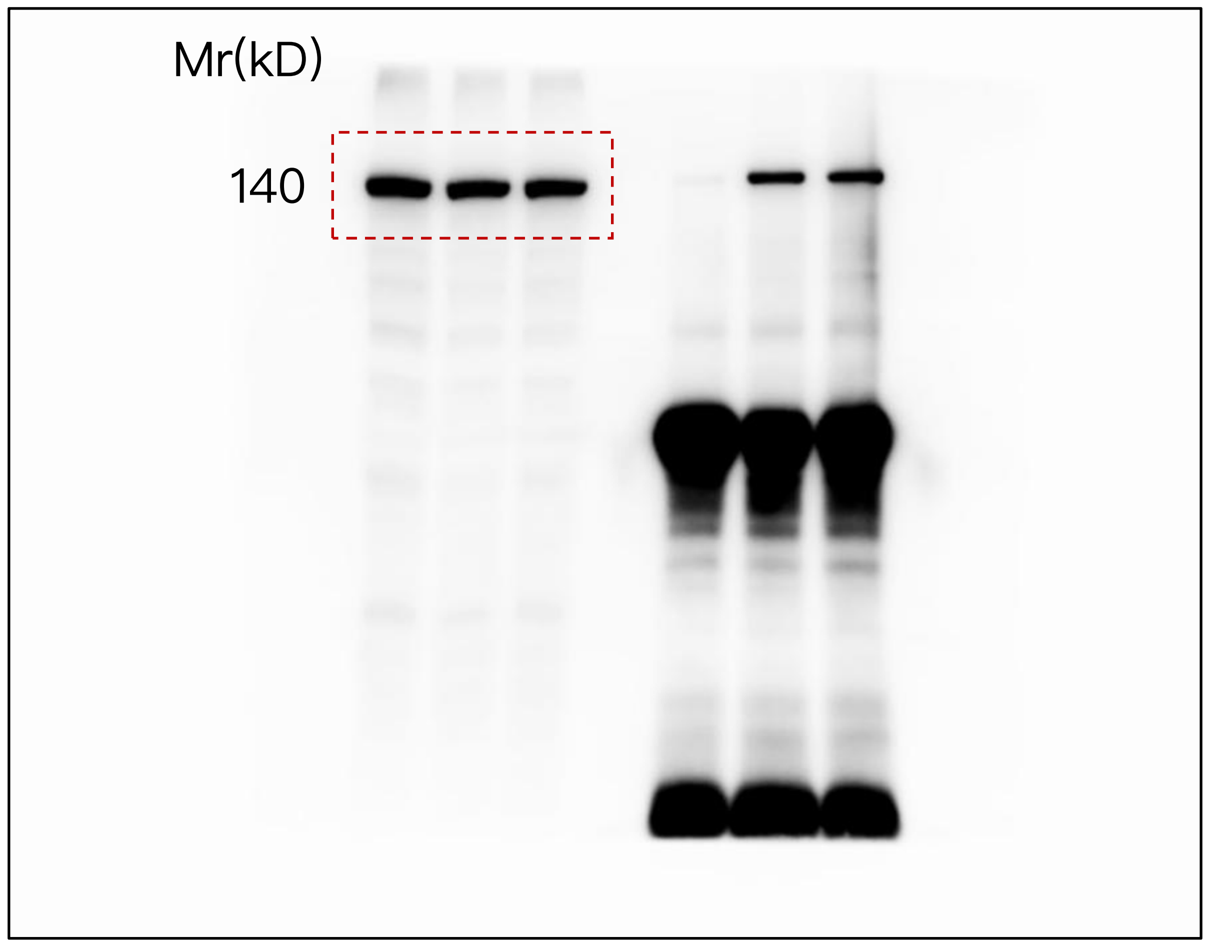

Figure 3C IP anti–HA

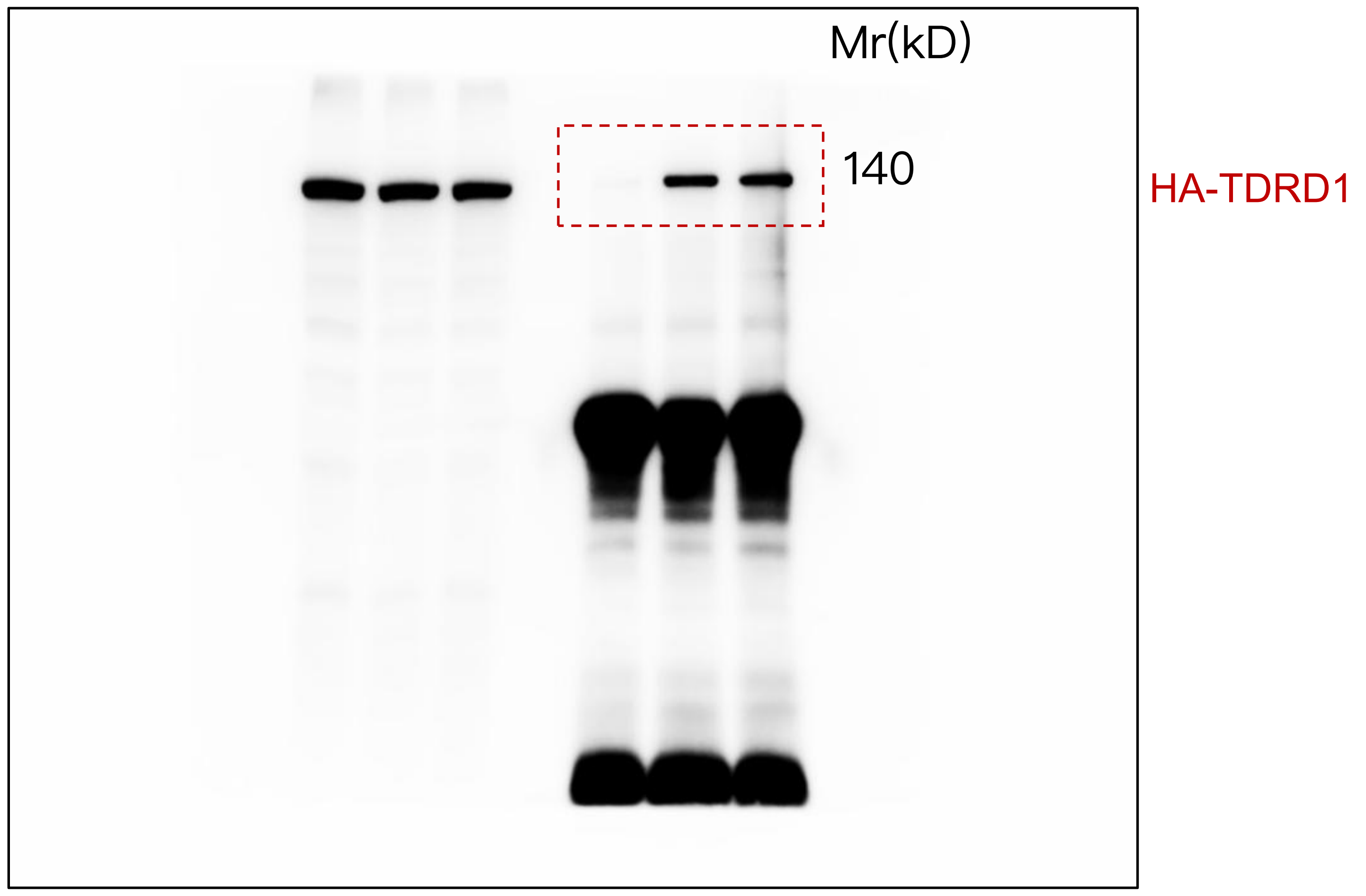

Figure 3C Input anti–Flag

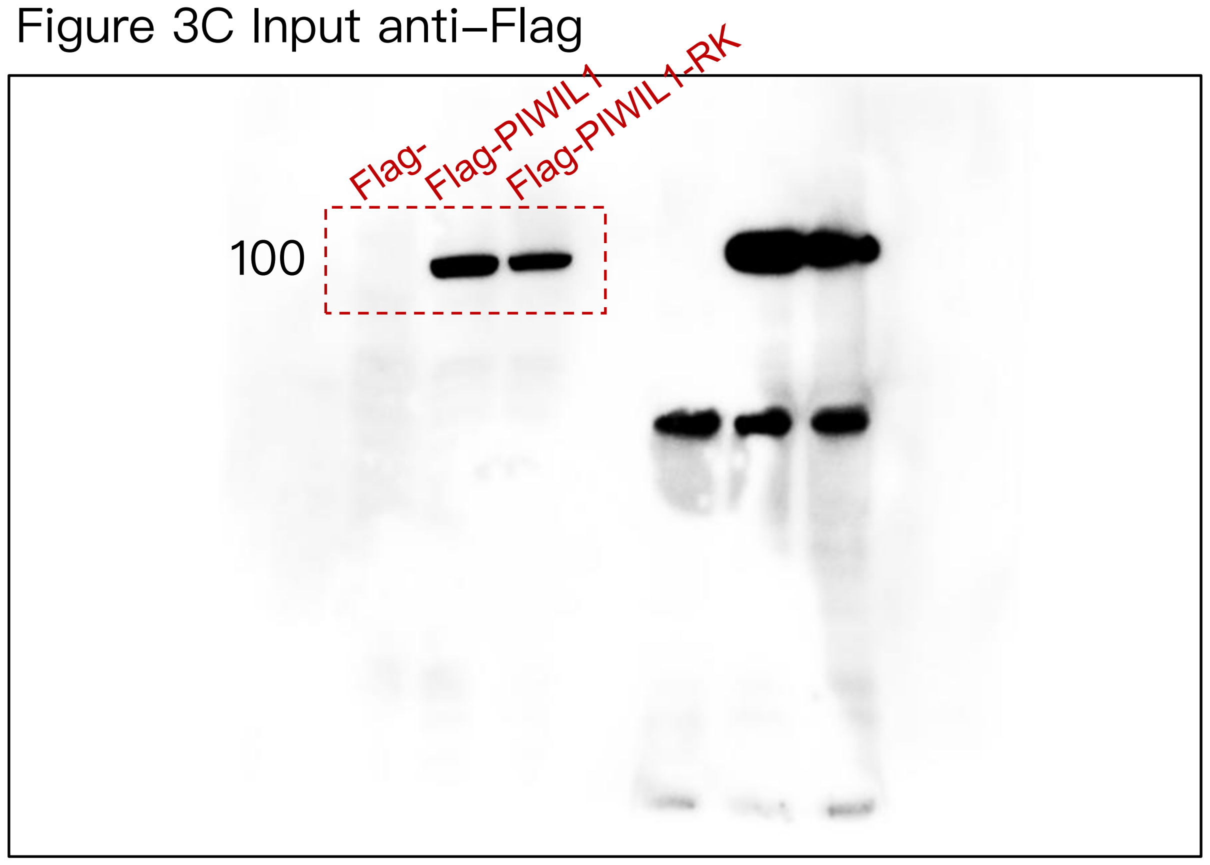

Figure 3C IP anti–Flag

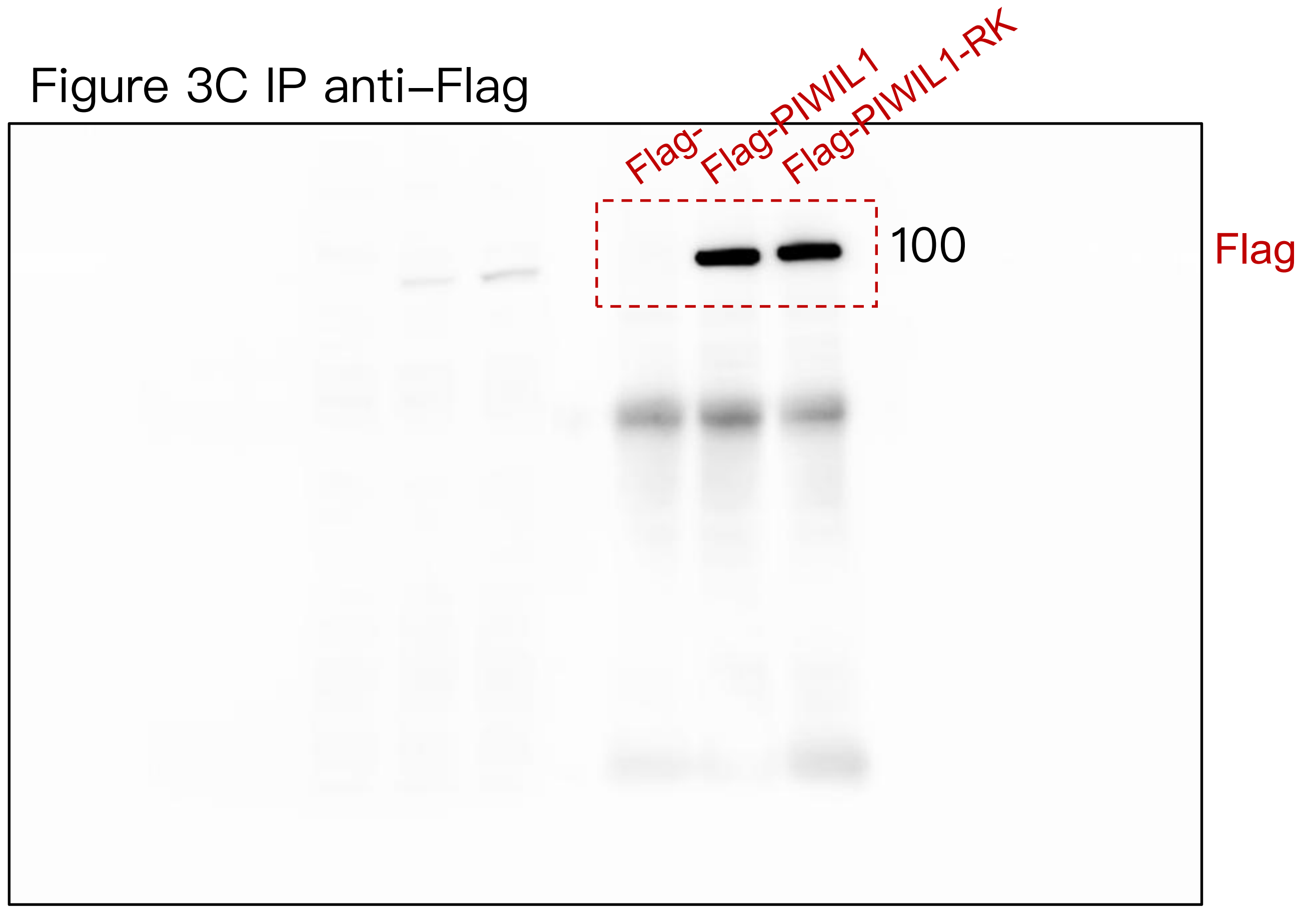

Supplement: Supplementary file 8 — Source data Fig. 3 [file 44318_2025_579_MOESM8_ESM.zip › Figure 3/3C/Figure 3C.pdf]
