# Supplementary material for: PIWI proteins tether the piRNA biogenesis machinery to mitochondria during mammalian spermatogenesis
Source: EMBO J. 2025 Sep 29;44(22):6397–424. doi: 10.1038/s44318-025-00579-x (PMC12624062; doi:10.1038/s44318-025-00579-x)
Supplement: Supplementary file 8 — Source data Fig. 3 [file 44318_2025_579_MOESM8_ESM.zip › Figure 3/3E/Figure 3E.pdf]

Figure 3E Input anti–HA

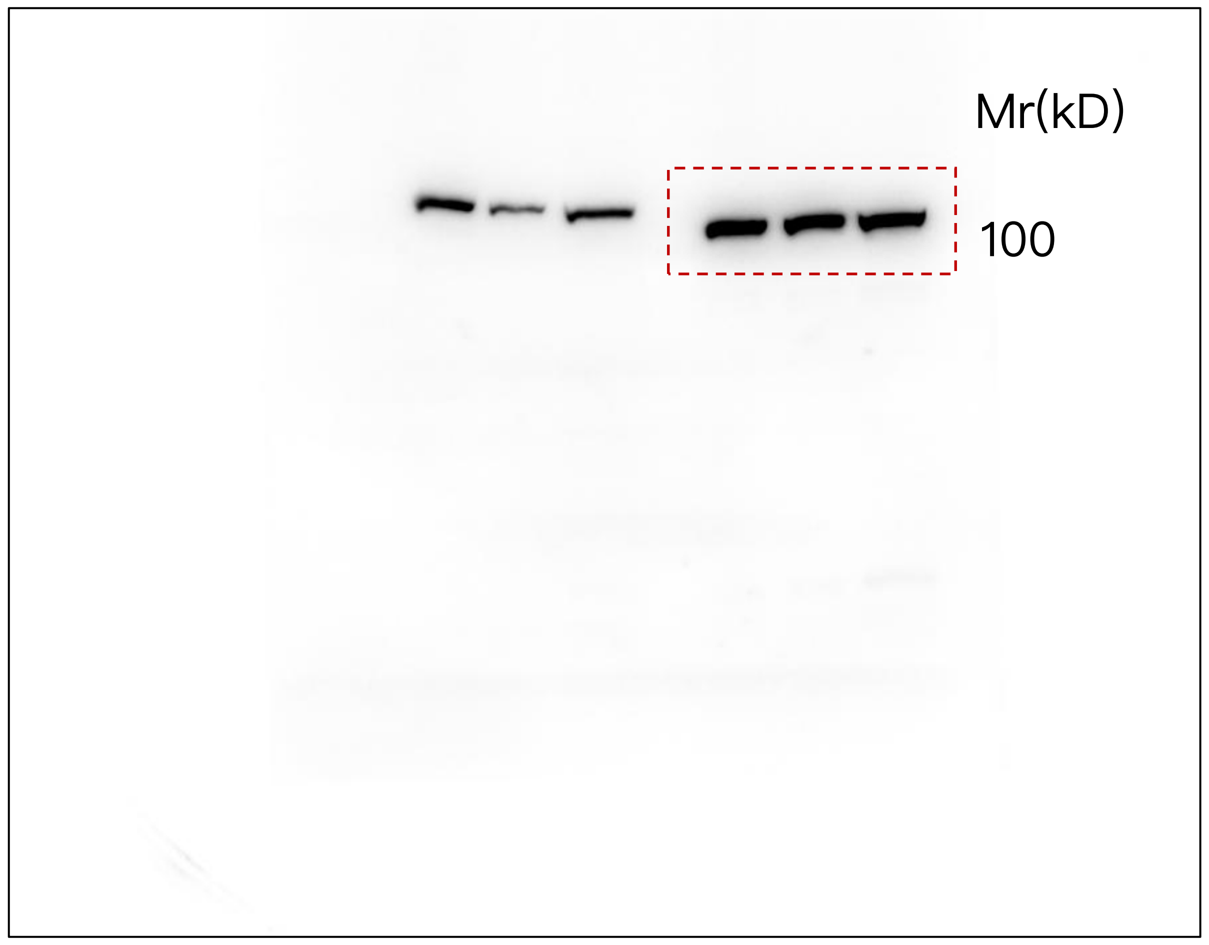

Figure 3E IP anti–HA

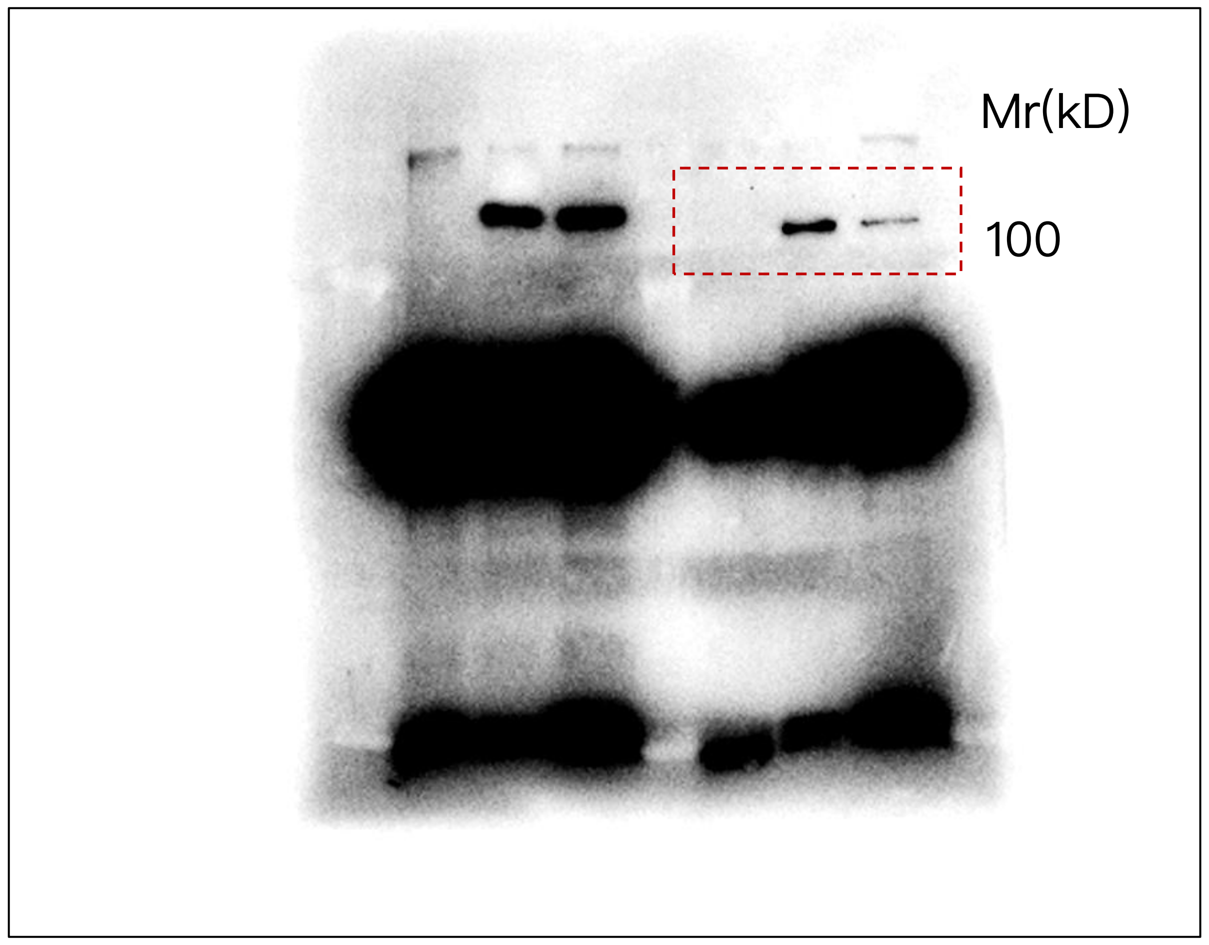

HA-PIWIL1

Figure 3E Input anti–Flag

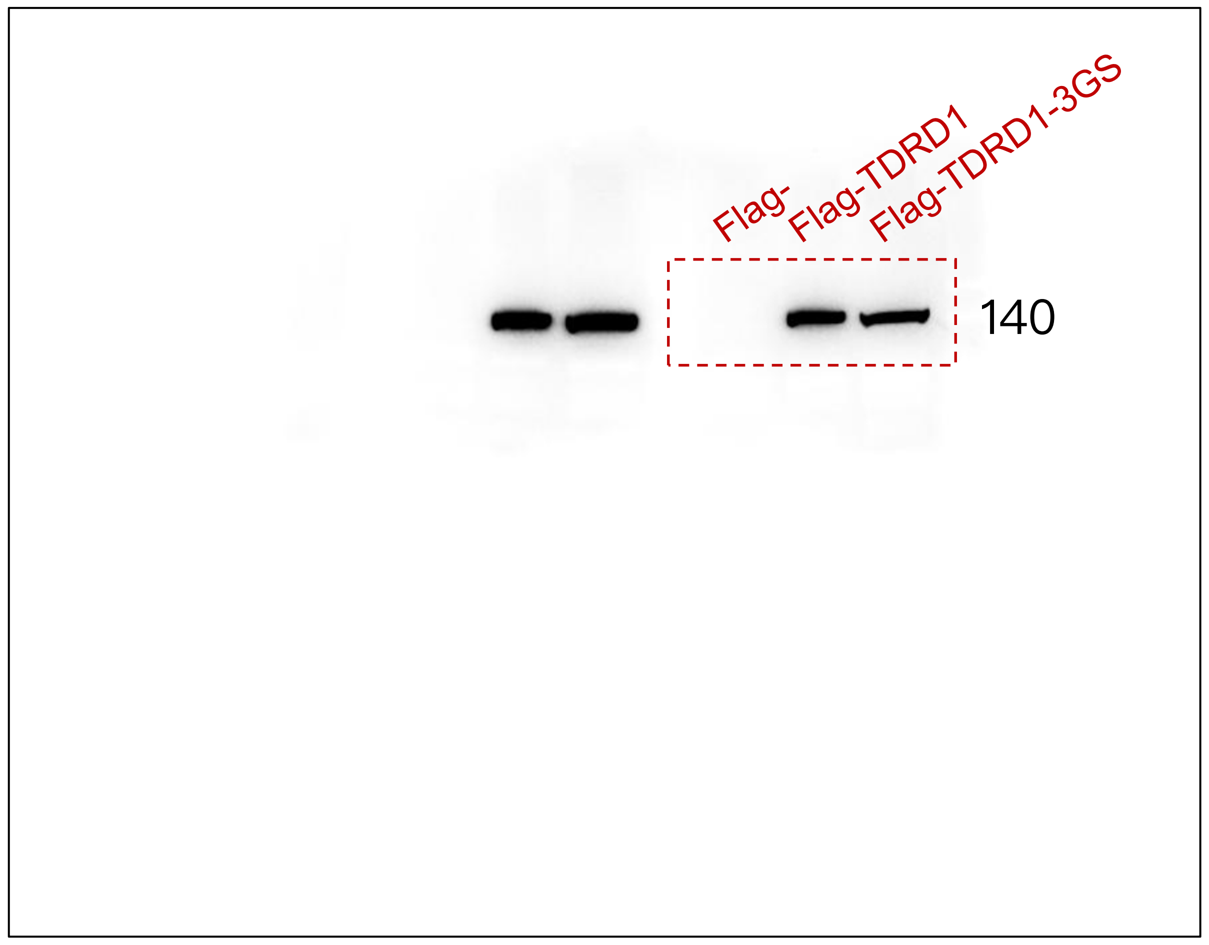

Figure 3E IP anti–Flag

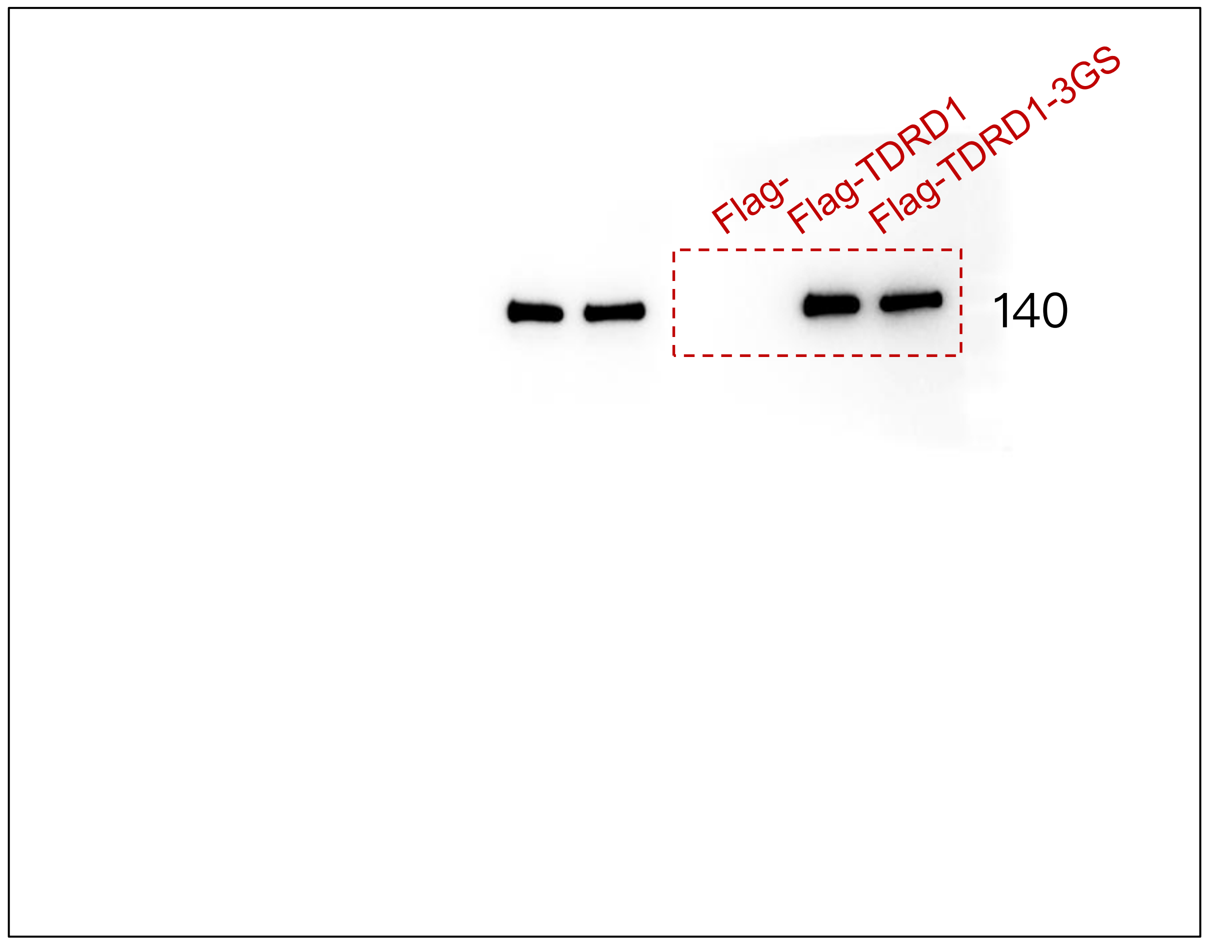

Flag
