# Supplementary material for: PIWI proteins tether the piRNA biogenesis machinery to mitochondria during mammalian spermatogenesis
Source: EMBO J. 2025 Sep 29;44(22):6397–424. doi: 10.1038/s44318-025-00579-x (PMC12624062; doi:10.1038/s44318-025-00579-x)

Figure 6I anti–TDRD1

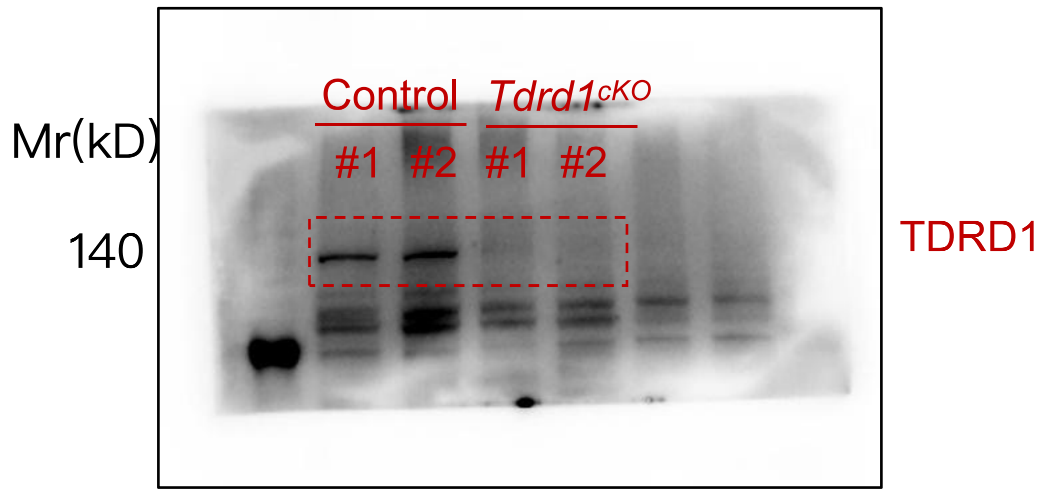

Figure 6I anti–PIWIL2

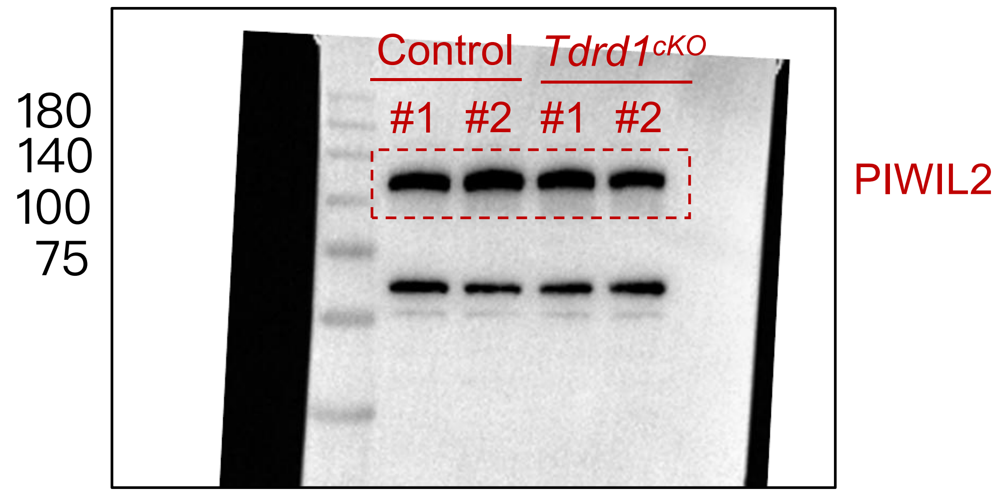

Figure 6I anti–PIWIL1

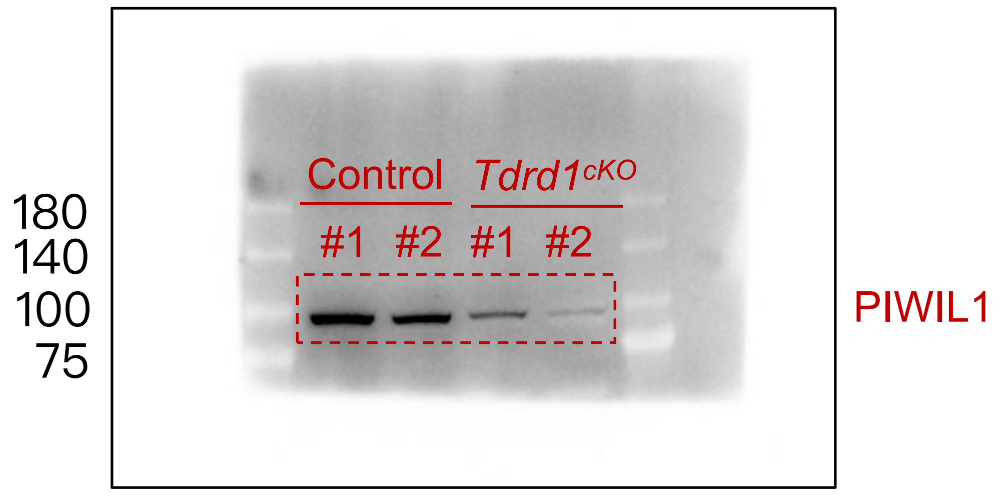

Figure 6I anti–TDRKH

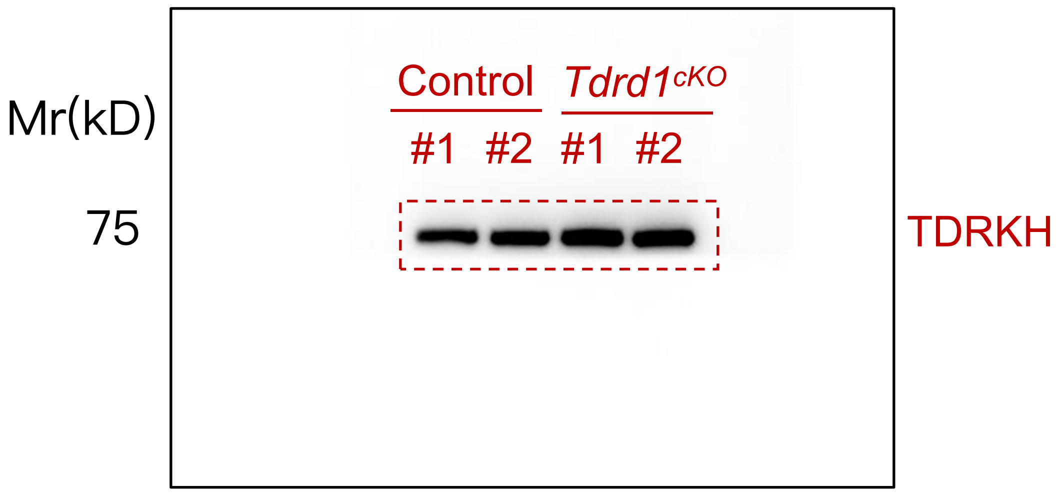

Figure 6I anti–ASZ1

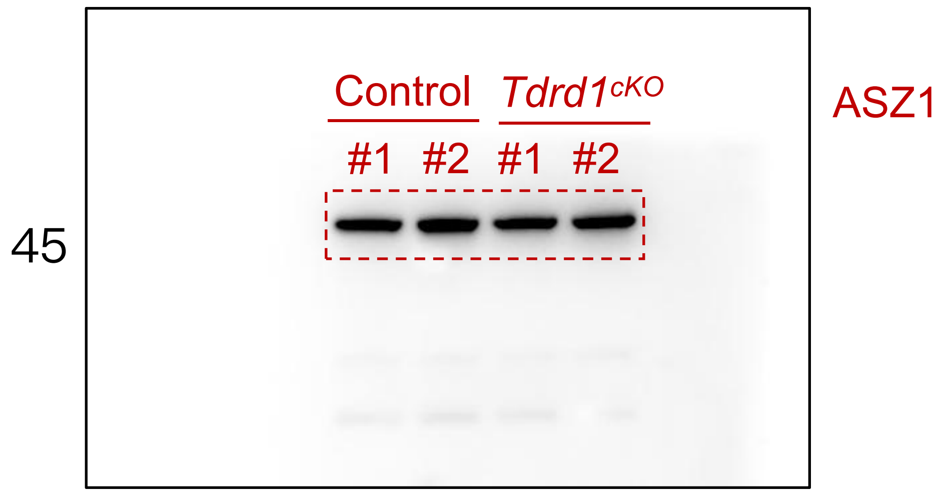

Figure 6I anti–β-actin

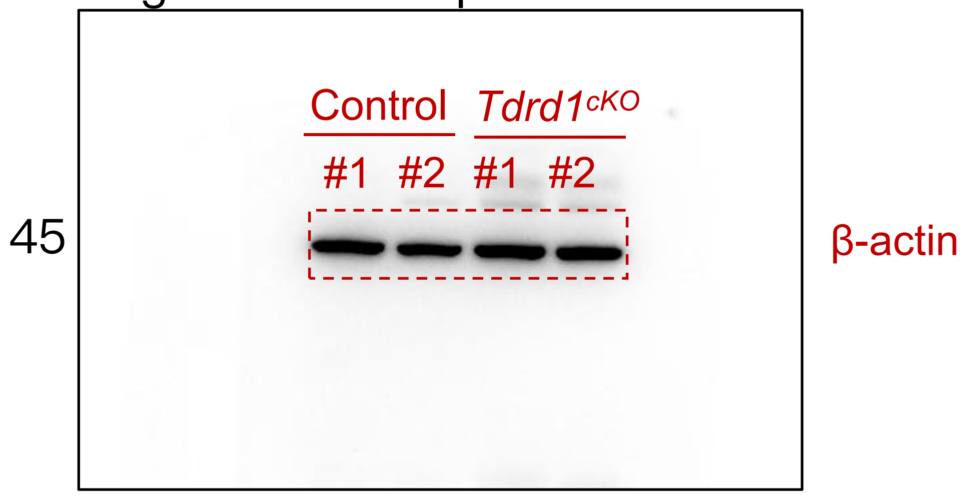

Supplement: Supplementary file 11 — Source data Fig. 6 [file 44318_2025_579_MOESM11_ESM.zip › Figure 6/6I/Figure 6I.pdf]
