# Supplementary material for: PIWI proteins tether the piRNA biogenesis machinery to mitochondria during mammalian spermatogenesis
Source: EMBO J. 2025 Sep 29;44(22):6397–424. doi: 10.1038/s44318-025-00579-x (PMC12624062; doi:10.1038/s44318-025-00579-x)

Figure 7L anti–PIWIL2

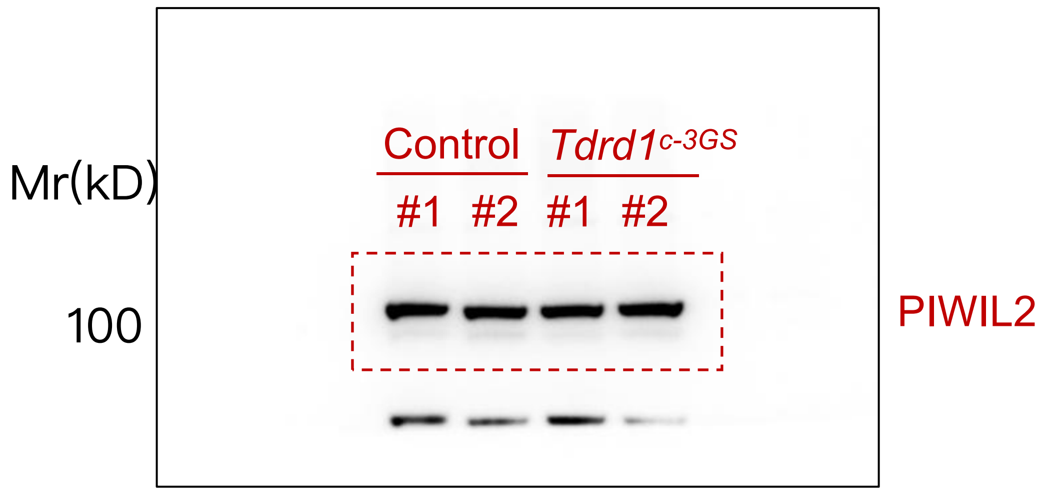

Figure 7L anti–ASZ1

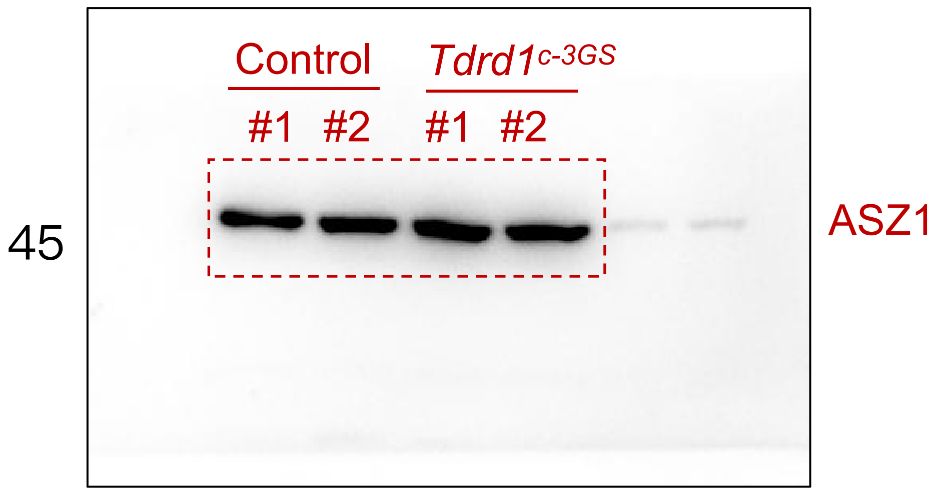

Figure 7L anti–PIWIL1

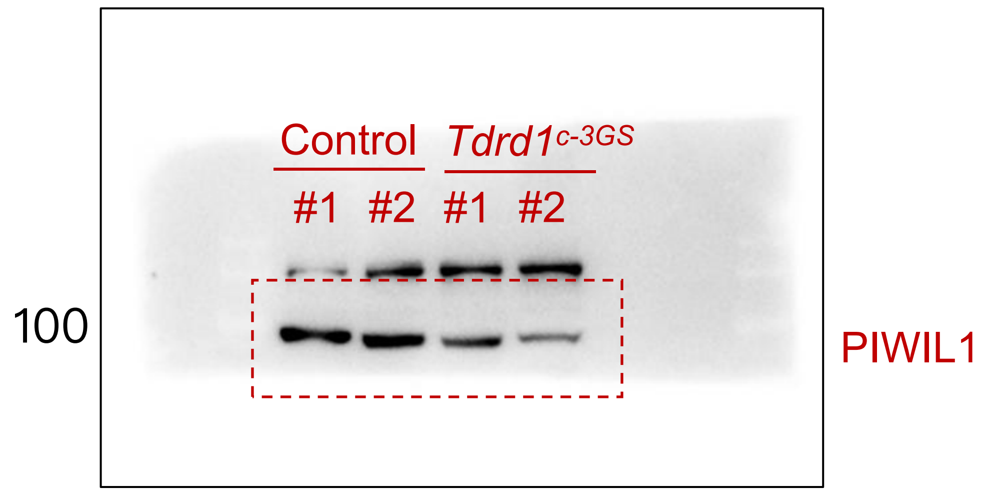

Figure 7L anti–β-actin

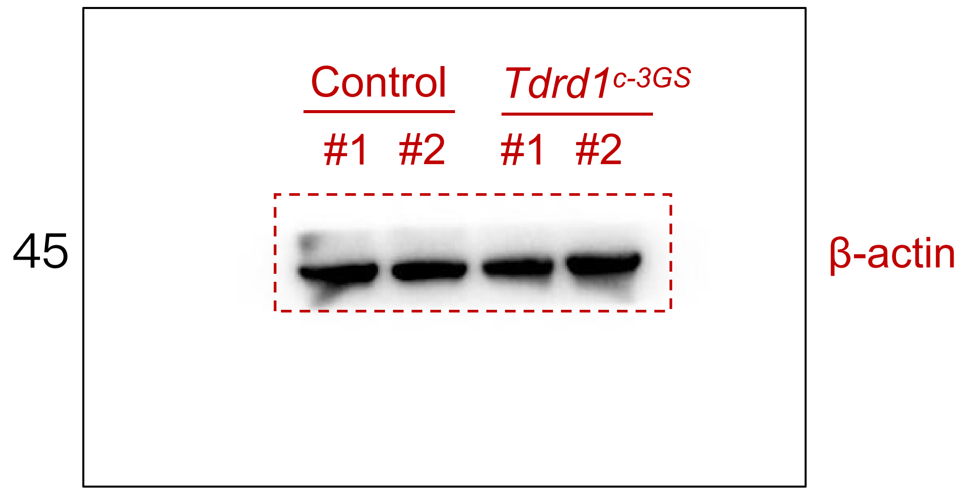

Figure 7L anti–TDRKH

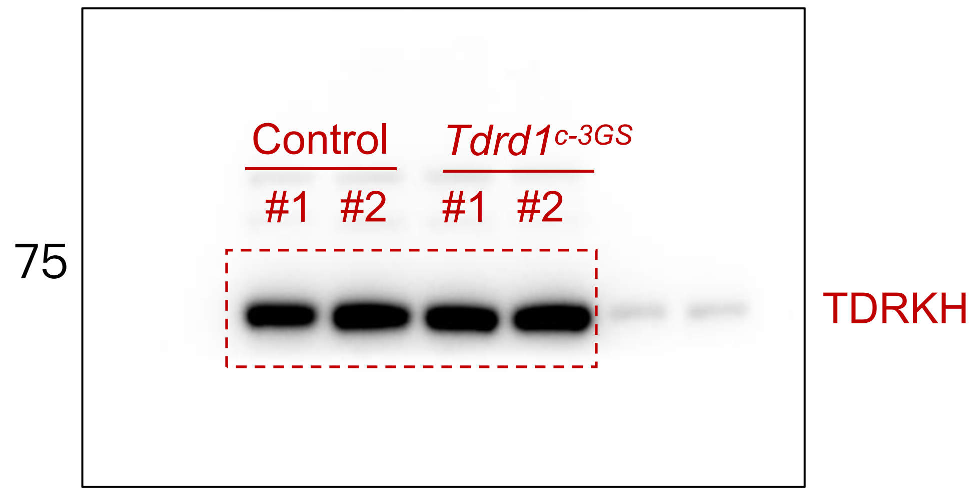

Supplement: Supplementary file 12 — Source data Fig. 7 [file 44318_2025_579_MOESM12_ESM.zip › Figure 7/7L/Figure 7L.pdf]
