# Supplementary material for: PIWI proteins tether the piRNA biogenesis machinery to mitochondria during mammalian spermatogenesis
Source: EMBO J. 2025 Sep 29;44(22):6397–424. doi: 10.1038/s44318-025-00579-x (PMC12624062; doi:10.1038/s44318-025-00579-x)
Supplement: Supplementary file 13 — Expanded View Figures [file 44318_2025_579_MOESM13_ESM.pdf]

Expanded View Figures

Figure EV1. PIWIL2 serves as an adapter to form the ASZ1-PIWIL2-TDRD1 complex.

(**A**) Interaction networks of ASZ1 and PIWIL2 based on IP/MS from P20 testes. The known piRNA-related proteins are highlighted by red circles. (**B**) Co-IP assay in 293T cells transfected with indicated constructs. Flag-tagged and GFP-tagged proteins were detected by WB. (**C**) 293T cells were treated with methyltransferase inhibitor MTA. WB was performed using sDMA (SYM10) or aDMA (ASYM24) antibodies. β-actin served as a control. (**D**, **E**) Co-IP assay in transfected 293T cells with MTA treatment. Flag-tagged and HA-tagged proteins were detected by WB. (**F**) HeLa cells were transfected with indicated constructs. Immunostaining was performed using the HA antibody. Scale bars, 10 µm. Fluorescence intensity through positions denoted by the white lines are shown in right. (**G**) Image of TEM on HeLa cells transfected with GFP-TDRD1, HA-PIWIL2, or RFP-ASZ1, respectively. Scale bars, 1 µm.

© The Author(s)

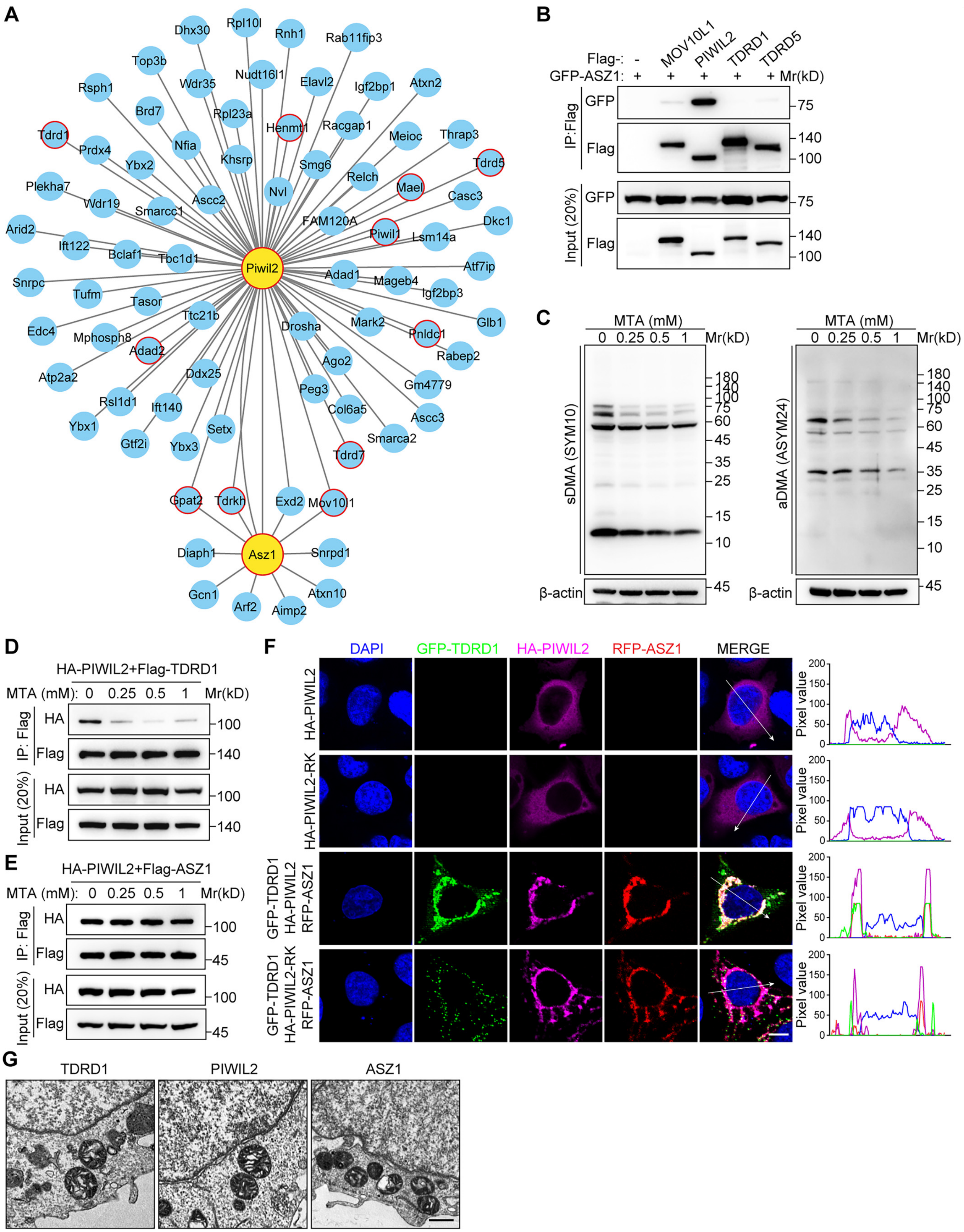
A
Piwil2
Asz1
B
Flag-: - MOV10L1 PIWIL2 TDRD1 TDRD5
GFP-ASZ1: + + + + + Mr(kD)
IP:Flag
Input (20%)
GFP
Flag
C
MTA (mM)
0 0.25 0.5 1 Mr(kD)
sDMA (SYM10)
aDMA (ASYM24)
β-actin
D
HA-PIWIL2+Flag-TDRD1
MTA (mM): 0 0.25 0.5 1 Mr(kD)
IP: Flag
Input (20%)
HA
Flag
E
HA-PIWIL2+Flag-ASZ1
F
DAPI
GFP-TDRD1
HA-PIWIL2
RFP-ASZ1
MERGE
HA-PIWIL2-RK
Pixel value
G
TDRD1
PIWIL2
ASZ1

 © The Author(s)

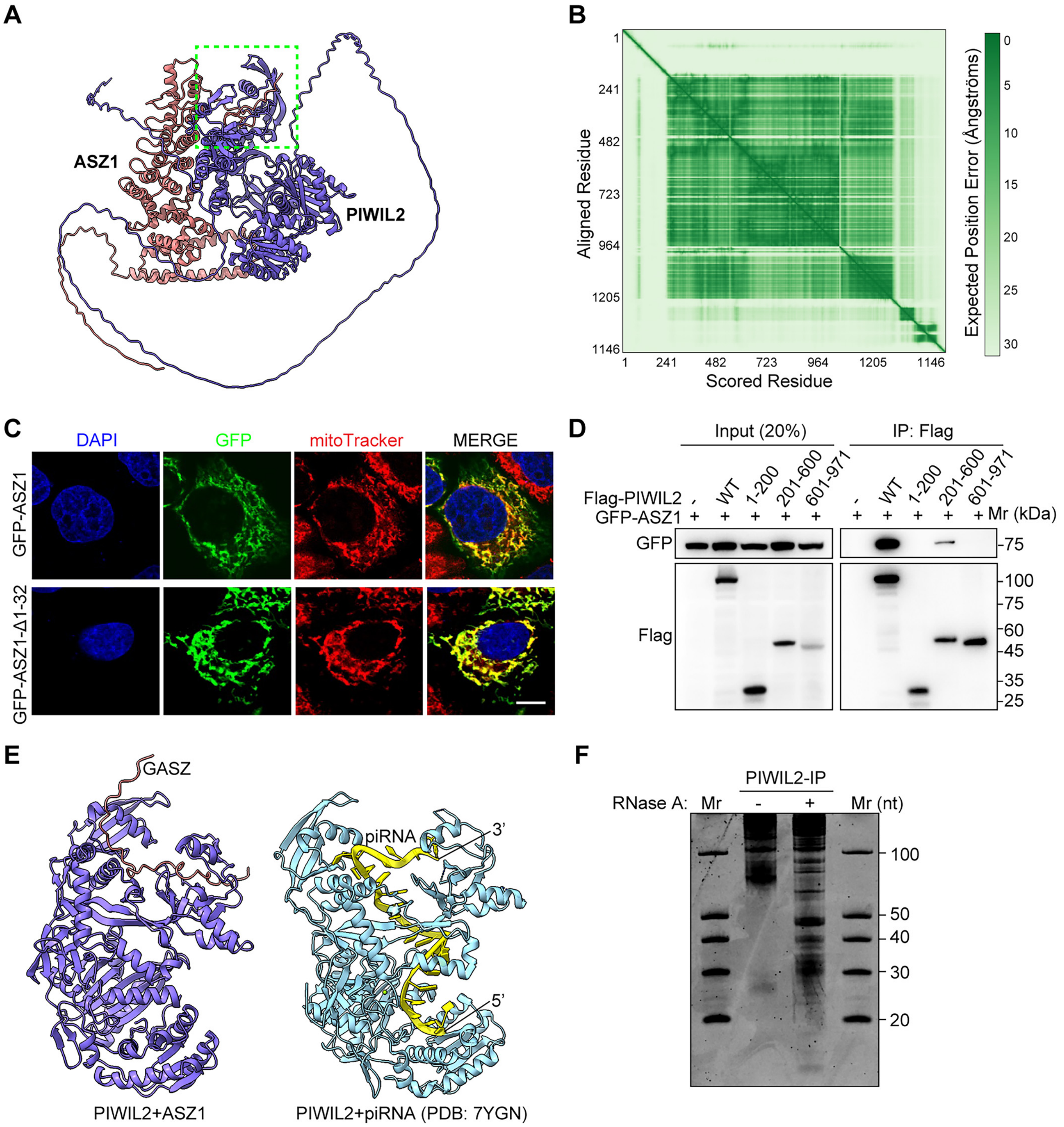

Figure EV2. piRNA loading onto PIWIL2 disrupts ASZ1-PIWIL2 interaction.

(**A**) Interaction structure model of the PIWIL2-ASZ1 complex predicted by AlphaFold. The green box highlights the interaction interface. (**B**) PAE (predicted aligned error) plot showing regions of high confidence (dark green) and low confidence (pale green) for the predicted structure of PIWIL2 and ASZ1 complex. (**C**) HeLa cells were transfected with indicated constructs. Transfected cells were stained with Mitotracker to label mitochondria. Scale bars, 10 µm. (**D**) Co-IP assay in 293T cells transfected with indicated constructs. Flag-tagged and GFP-tagged proteins were detected by WB. (**E**) Left, PIWIL2-ASZ1 structure predicted by AlphaFold; right, PIWIL2-piRNA structure from PDB (7YGN). (**F**) PIWIL2 bound RNAs from adult testes with or without RNase A treatment were detected by Urea-PAGE gel.

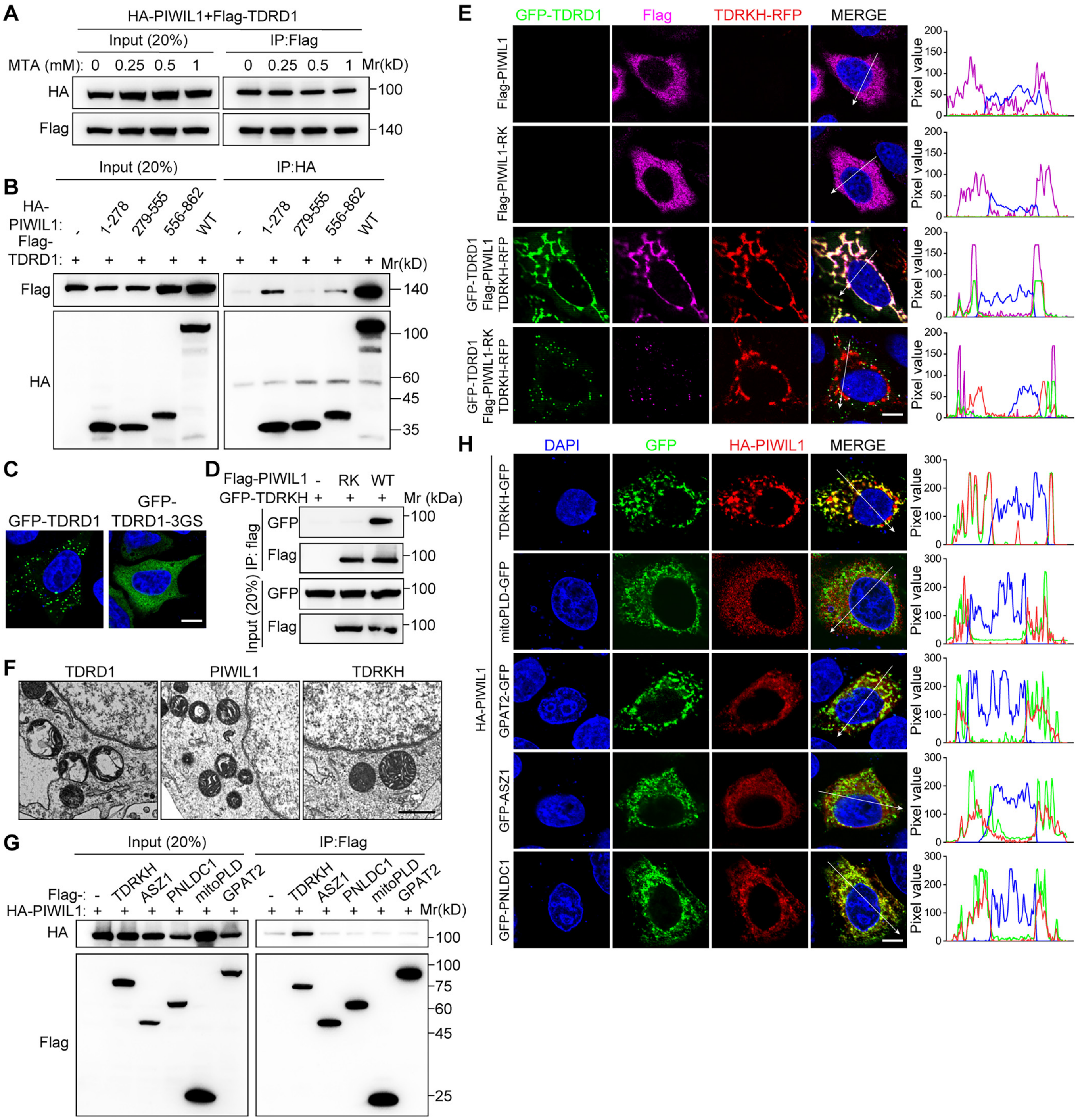

Figure EV3. TDRKH-PIWIL1 complex cooperates with ASZ1-PIWIL2 complex to recruit TDRD1 to mitochondria.

(**A**) Co-IP assay in transfected 293T cells with MTA treatment. Flag-tagged and HA-tagged proteins were detected by WB. (**B**) Co-IP assay in 293T cells transfected with indicated constructs. Flag-tagged and HA-tagged proteins were detected by WB. (**C**) Images of HeLa cells transfected with the indicated plasmids. Scale bars, 10 μm. (**D**) Co-IP assay in 293T cells transfected with indicated constructs. Flag-tagged and GFP-tagged proteins were detected by WB. (**E**) HeLa cells were transfected with indicated constructs. Immunostaining was performed using the Flag antibody. Scale bars, 10 μm. Fluorescence intensity through positions denoted by the white lines are shown in right. (**F**) Image of TEM on HeLa cells transfected with GFP-TDRD1, Flag-PIWIL1, or TDRKH-RFP, respectively. Scale bars, 1 μm. (**G**) Co-IP assay in 293T cells transfected with indicated constructs. Flag-tagged and HA-tagged proteins were detected by WB. (**H**) HeLa cells were transfected with indicated constructs. Immunostaining was performed using the HA antibody. Scale bars, 10 μm. Fluorescence intensity through positions denoted by the white lines are shown in right.

 © The Author(s)

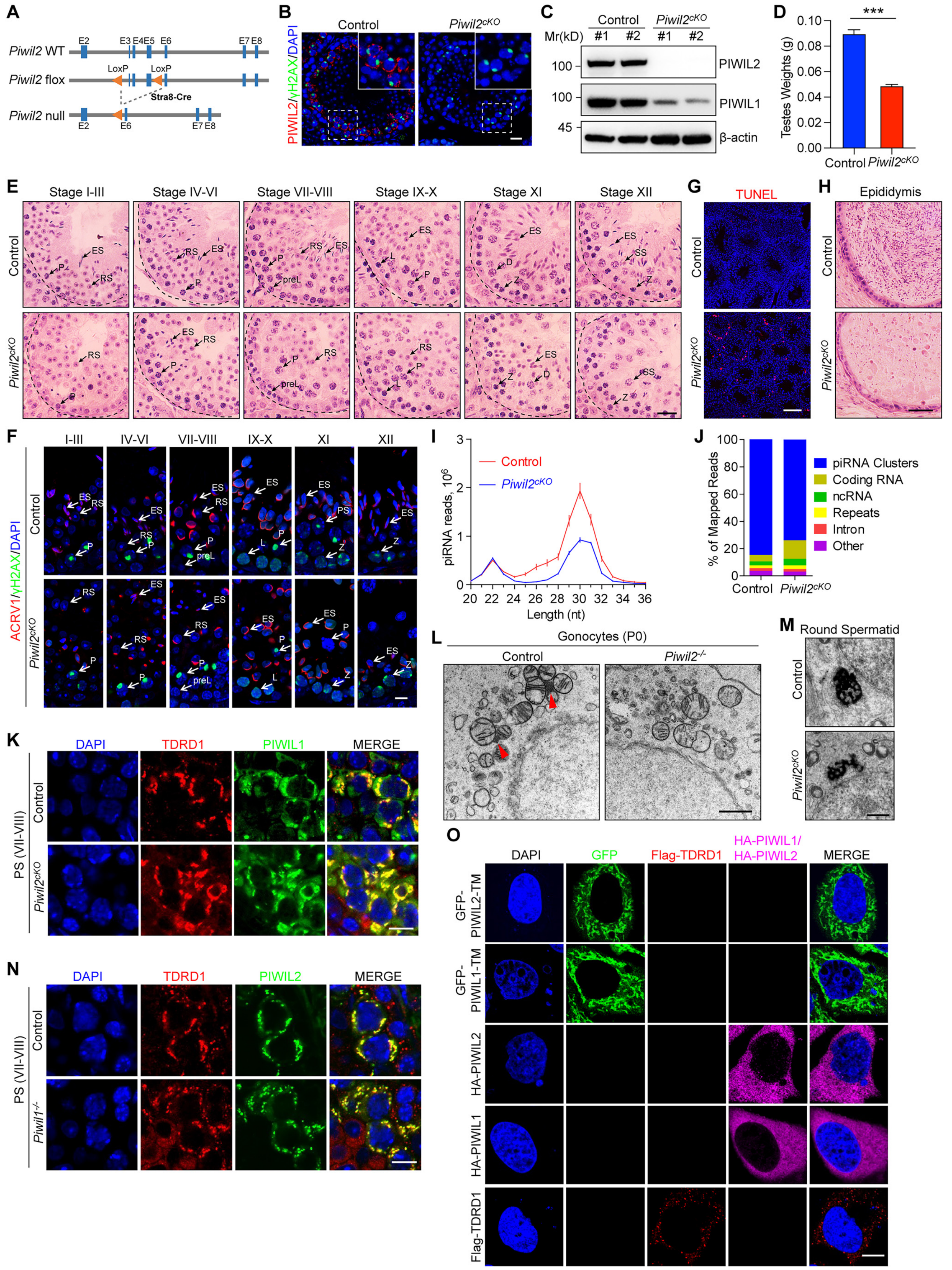
A
Piwil2 WT
E2 E3 E4E5 E6 E7 E8
Piwil2 flox
LoxP LoxP
Stra8-Cre
Piwil2 null
E2 E6 E7 E8
B
Control Piwil2cKO
PIWIL2/γH2AX/DAPI
C
Control Piwil2cKO
Mr(kD) #1 #2 #1 #2
100 PIWIL2
100 PIWIL1
45 β-actin
D
***
Testes Weights (g)
0.10 0.08 0.06 0.04 0.02 0.00
Control Piwil2cKO
E
Stage I-III Stage IV-VI Stage VII-VIII Stage IX-X Stage XI Stage XII
Control
Piwil2cKO
ES RS P preL L D Z SS
G
TUNEL
Control
Piwil2cKO
H
Epididymis
Control
Piwil2cKO
F
I-III IV-VI VII-VIII IX-X XI XII
ACRV1/γH2AX/DAPI
Control
Piwil2cKO
ES RS P preL L Z PS
I
Control
Piwil2cKO
piRNA reads, 10^6
Length (nt)
20 22 24 26 28 30 32 34 36
J
% of Mapped Reads
piRNA Clusters
Coding RNA
ncRNA
Repeats
Intron
Other
Control Piwil2cKO
K
DAPI TDRD1 PIWIL1 MERGE
PS (VII-VIII)
Control
Piwil2cKO
L
Gonocytes (P0)
Control Piwil2-/-
M
Round Spermatid
Control
Piwil2cKO
N
DAPI TDRD1 PIWIL2 MERGE
PS (VII-VIII)
Control
Piwil1-/-
O
DAPI GFP Flag-TDRD1 HA-PIWIL1/ HA-PIWIL2 MERGE
GFP-PIWIL2-TM
GFP-PIWIL1-TM
HA-PIWIL2
HA-PIWIL1
Flag-TDRD1

Figure EV4. PIWIL2 is required for pachytene piRNA biogenesis.

(**A**) A schematic diagram showing the gene targeting strategy for the generation of a *Piwil2* conditional allele. Cre-mediated deletion removed the exon 3–5 of *Piwil2* and generated a protein null allele. (**B**) Co-immunostaining of PIWIL2 and γH2AX on control and *Piwil2*^{cKO} adult testes. Scale bars, 20 μm. (**C**) WB of PIWIL2 and PIWIL1 expression in control and *Piwil2*^{cKO} adult testes. β-actin served as a control. (**D**) The average weight of adult testes ($n = 6$; ***$p < 0.001$). (**E**) H&E staining on adult testes. preL preleptotene, L leptotene, Z zygotene, P pachytene, D diplotene, RS round spermatid, ES elongated spermatid, SS secondary spermatocytes. Scale bars, 20 μm. (**F**) Co-immunostaining of ACRV1 and γH2AX on adult mouse testes. preL preleptotene, L leptotene, Z zygotene, P pachytene, D diplotene, M metaphase, RS round spermatid, ES elongated spermatid; Scale bars, 10 μm. (**G**) TUNEL assays on adult testes. Scale bars, 100 μm. (**H**) H&E staining of the mouse adult epididymis. Scale bars, 50 μm. (**I**) The length distribution of PIWIL1-piRNAs from adult testes. Data were normalized by total reads. $n = 2$. (**J**) Genomic annotation of PIWIL1-piRNAs from control and *Piwil2*^{cKO} adult testes. Data were representative of two biological replicates. (**K**) Co-immunostaining of TDRD1 and PIWIL1 on control and *Piwil2*^{cKO} adult testes. Stage VII–VIII seminiferous tubules were distinguished according to DAPI staining. PS pachytene spermatocytes. Scale bars, 10 μm. (**L**) Images of TEM on gonocytes from mouse P0 testes. IMCs were indicated by red arrowheads. Scale bars, 1 μm. (**M**) Images of TEM on round spermatids from adult testes showing CB structure. Scale bars, 1 μm. (**N**) Co-immunostaining of TDRD1 and PIWIL2 on control and *Piwil1*$^{-/-}$ adult testes. Stage VII–VIII seminiferous tubules were distinguished according to DAPI staining. PS pachytene spermatocytes. Scale bars, 10 μm. (**O**) HeLa cells were transfected with indicated constructs. Immunostaining was performed using Flag, PIWIL1, and PIWIL2 antibodies. Scale bars, 10 μm. Data information: In (**D**, **I**), data were presented as mean ± s.e.m. In (**D**), p values were calculated using Student's t-test (***$p < 0.001$). $p = 3.1469E\text{-}07$ (**D**).

 © The Author(s)

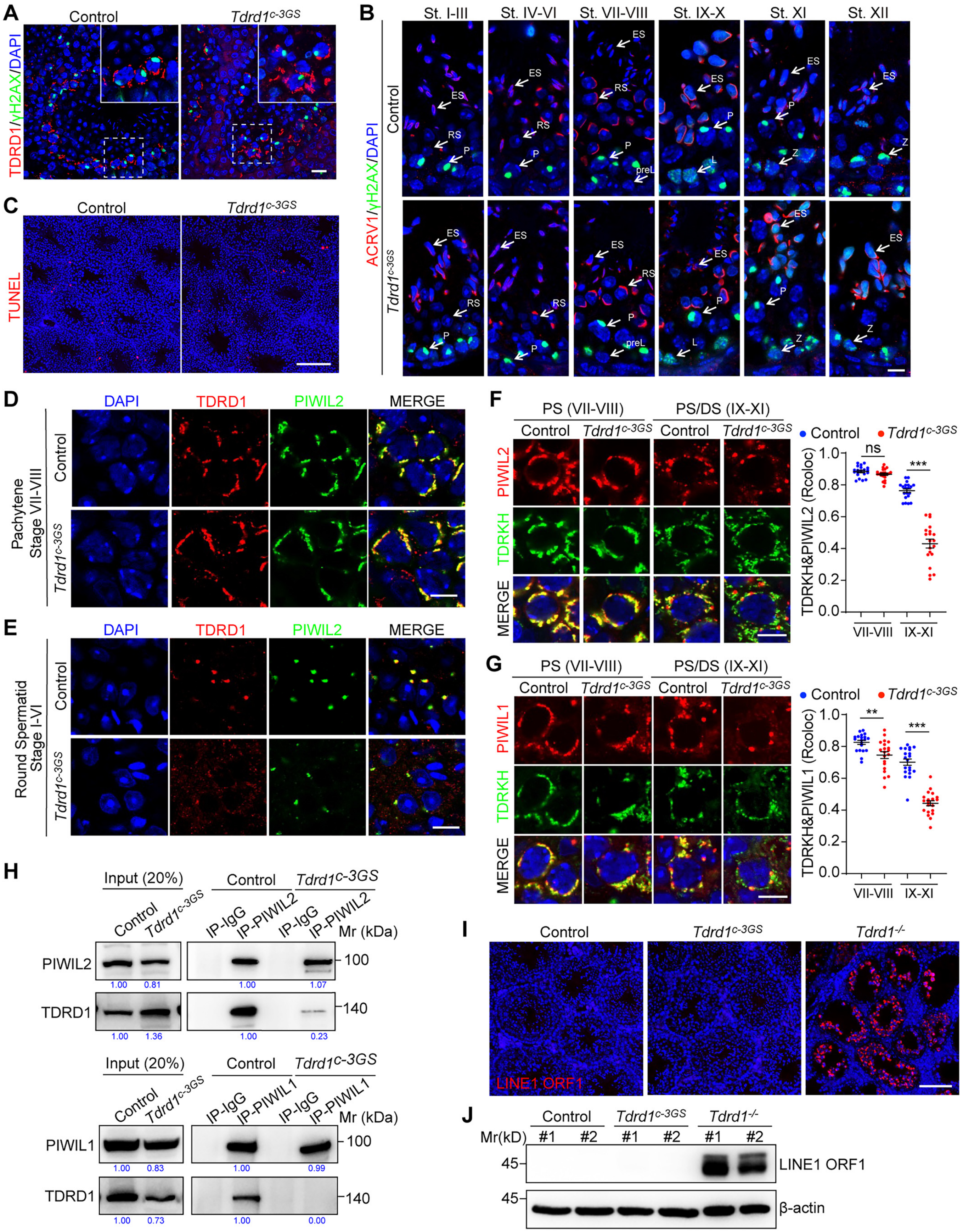
A
Control
Tdrd1c-3GS
TDRD1/γH2AX/DAPI
B
St. I-III
St. IV-VI
St. VII-VIII
St. IX-X
St. XI
St. XII
ACRV1/γH2AX/DAPI
Control
Tdrd1c-3GS
ES
RS
P
preL
L
Z
C
Control
Tdrd1c-3GS
TUNEL
D
DAPI
TDRD1
PIWIL2
MERGE
Pachytene Stage VII-VIII
Control
Tdrd1c-3GS
E
DAPI
TDRD1
PIWIL2
MERGE
Round Spermatid Stage I-VI
Control
Tdrd1c-3GS
F
PS (VII-VIII)
PS/DS (IX-XI)
Control
Tdrd1c-3GS
Control
Tdrd1c-3GS
PIWIL2
TDRKH
MERGE
Control
Tdrd1c-3GS
ns
***
TDRKH&PIWIL2 (Rcoloc)
1.0
0.8
0.6
0.4
0.2
0.0
VII-VIII
IX-XI
G
PS (VII-VIII)
PS/DS (IX-XI)
Control
Tdrd1c-3GS
Control
Tdrd1c-3GS
PIWIL1
TDRKH
MERGE
Control
Tdrd1c-3GS
**
***
TDRKH&PIWIL1 (Rcoloc)
1.0
0.8
0.6
0.4
0.2
0.0
VII-VIII
IX-XI
H
Input (20%)
Control
Tdrd1c-3GS
Control
Tdrd1c-3GS
IP-IgG
IP-PIWIL2
IP-IgG
IP-PIWIL2
Mr (kDa)
PIWIL2
100
1.00
0.81
1.00
1.07
TDRD1
140
1.00
1.36
1.00
0.23
Input (20%)
Control
Tdrd1c-3GS
Control
Tdrd1c-3GS
IP-IgG
IP-PIWIL1
IP-IgG
IP-PIWIL1
Mr (kDa)
PIWIL1
100
1.00
0.83
1.00
0.99
TDRD1
140
1.00
0.73
1.00
0.00
I
Control
Tdrd1c-3GS
Tdrd1-/-
LINE1 ORF1
J
Control
Tdrd1c-3GS
Tdrd1-/-
Mr(kD)
#1
#2
#1
#2
#1
#2
45
LINE1 ORF1
45
β-actin

Figure EV5. TDRD1 phase separation is required for IMC assembly, piRNA biogenesis, and spermiogenesis in adult testes.

(**A**) Co-immunostaining of TDRD1 and γH2AX on control and *Tdrd1*$^{c\text{-}3GS}$ adult testes. Scale bars, 20 µm. (**B**) Co-immunostaining of ACRV1 and γH2AX on adult mouse testes. preL preleptotene, L leptotene, Z zygotene, P pachytene, D diplotene, RS round spermatid, ES elongated spermatid. Scale bars, 10 µm. (**C**) TUNEL assays on adult testes. Scale bars, 200 µm. (**D**, **E**) Co-immunostaining of TDRD1 and PIWIL2 on pachytene spermatocytes (**D**) and round spermatids (**E**) from adult testes. Scale bars, 10 µm. (**F**, **G**) Co-immunostaining of PIWIL2-TDRKH (**F**) or PIWIL1-TDRKH (**G**) on adult testes. PS pachytene spermatocytes, DS diplotene spermatocytes. Scale bars, 10 µm. Quantification of the colocalization ratio is shown on the right. ($n = 20$; $^{**}p < 0.01$; $^{***}p < 0.001$; ns not significant). The developmental stages of germ cells were distinguished according to DAPI staining. (**H**) Co-IP assay of PIWIL2 and PIWIL1 in control and *Tdrd1*$^{c\text{-}3GS}$ adult testes. PIWIL2, PIWIL1, and TDRD1 protein levels were detected by WB. (**I**) Immunostaining of LINE1 ORF1 on adult testes. Scale bars, 50 µm. (**J**) WB of LINE1 ORF1 in adult testes. β-actin served as a control. Data information: In (**F**, **G**), data were presented as mean ± s.e.m and *p* values were calculated using Student's *t*-test ($^{**}p < 0.01$; $^{***}p < 0.001$; ns not significant). *p* values from left to right (**F**): $p = 0.1052$, $p = 2.1886E\text{-}13$; *p* values from left to right (**G**): $p = 0.0026$, $p = 2.6065E\text{-}12$.

© The Author(s)
